# Supplementary material for: Multimorbidity, health service use, and health insurance by socioeconomic groups in 31 countries: A multi-cohort study
Source: PLoS Med. 2026 May 5;23(5):e1005087. doi: 10.1371/journal.pmed.1005087 (PMC13160435; doi:10.1371/journal.pmed.1005087)
Supplement: S1 Appendix — Table A: The definition of physical, psychological and cognitive disorder. Table B: The definition of healthcare use. Table C: The definition of health insurance. Table D: The definition of lifestyles. Table E: Basic demographics by study. Table F: Basic demographics by country. Table G: Marginal predicted counts. Table H: The statistical test results of multimorbidity*SES. Table I: The association between multimorbidity and outpatient by country. Table J: The association between multimorbidity and number of outpatient visits by country. Table K: The association between multimorbidity and inpatient by country. Table L: The association between multimorbidity and number of inpatient admissions by country. Table M: The meta-analysis details. Table N: The statistical test results of multimorbidity*health insurance. Table O: Association between each disorder domain and outpatient care by country. Table P: Association between each disorder domain and inpatient care by country. Table Q: Association between each disorder domain and number of outpatient visits by country. Table R: Association between each disorder domain and number of inpatient admissions by country. Table S: Variance components and ICC from mixed models. Table T: Changes in between-country heterogeneity (I2) (outpatient). Table U: Changes in between-country heterogeneity (I2) (inpatient). Table V: Changes in between-country heterogeneity (I2) (number of outpatient visits). Table W: Changes in between-country heterogeneity (I2) (number of inpatient admissions). Table X: Model fit comparison across models. Table Y: Predictors of attrition (logistic regression models). Fig A: Directed acyclic graph. Fig B: Prevalence of physical, psychological, and cognitive multimorbidity by country. Fig C: The OR and IRR differences between individuals with and without health insurance (grouped by multimorbidity and SES). Fig D: The association between healthcare utilisation and multimorbidity (specific cohorts and poisson m [file pmed.1005087.s001.docx]

**Appendix Tables**

Table A. The definition of physical, psychological and cognitive disorder.

|  |  | HRS (2008–2020) | SHARE (2008-2019) | KLOSA (2006-2020) | CHARLS (2011-2018) | MHAS (2001-2018) | JSTAR (2007-2011) |
| --- | --- | --- | --- | --- | --- | --- | --- |
| Physical disorder | Yes | hypertension, diabetes, stroke, heart problem, arthritis, lung disease, cancer | | | | | |
|  | No | Did not report above mentioned physical chronic condition. | | | | | |
| Psychological disorder | Yes | A CESD-8 score of greater than 3 ^1^ | EURO-D score ≥4 ^2^ | CES-D10 score >=10 ^3^ | Total CES-D score>=10 ^4,5^ | MHAS screening questionnaire total score of ≥5 points ^6^ | CES-D10 score >=10 ^7^ |
|  | No | <4 | <4 | <10 | <10 | <5 | <10 |
| Cognitive disorder | Yes | The Harmonized Cognitive Assessment Protocol (HCAP) of 10 or below ^8^ | Scored 1.5 SD below the age graded mean on both the SHARE memory and verbal fluency tasks ^9^ | K-MMSE≤23 ^10^ | A TICS score of 11 or less ^11^ | CCCE (Cross-Cultural Cognitive Examination) 1.5 SD below the mean of sum score | Scored 1.5 SD below the age graded mean on both the word recall ^12^ |
|  | No | >11 | >11 | >11 | Otherwise | >23 | >11 |

Table B. The definition of healthcare utilisation.

| Dataset | Outpatient | Number of outpatient visits | Inpatient | Number of inpatient admissions |
| --- | --- | --- | --- | --- |
| **HRS** | Whether the Respondent reports any doctor visit since the last interview, or the last 2 years for new interviewees ^1^. | The times has respondent seen or talked to a medical doctor about health, including emergency room or clinic visits since last interview or last two years before death. | whether the respondent reports any overnight hospital stay since the last interview, or the last 2 years for new interviewees ^2^. | The number of stays in a hospital between the last core interview and death. |
| **CHARLS** | Whether the respondent reported visiting a public hospital, private hospital, public health center, clinic, or health worker’s or doctor’s practice, or receiving a visit from a health worker or doctor for outpatient care in the past month ^3^. | The number of outpatient visits to the specified medical facilities in the past month. | Whether the respondent reported receiving any inpatient care in the past year. | The number of inpatient care episodes in the past year. |
| **JSTAR** | Whether the respondent reported any utilisation of medical centers in the previous year. | The number of visits to medical centers and primary care facilities reported per month. | Whether the respondent reported any overnight hospital stay in the previous year. | The number of overnight hospital stays reported. |
| **KLoSA** | Whether the respondent reported visiting a public health clinic or consulting a medical doctor since the previous interview (typically 2 years prior) ^4^. | The total number of visits to public health clinics and medical doctors since the last interview. | Whether the respondent reported being hospitalized in a hospital, nursing home, convalescent home, or other long-term care facility since the previous interview ^5^. | The number of hospitalizations in a hospital, nursing home, convalescent home, or long-term care facility reported since the previous interview. |
| **MAHS** | Whether the respondent reported at least one doctor visit in the past 12 months | The number of doctor visits reported in the past 12 months. | Whether the respondent reported at least one overnight hospital stay in the past 12 months. | - |
| **SHARE** | Whether the respondent reported any doctor visit in the past 12 months. | The number of doctor visits reported in the past 12 months. | Whether the respondent reported any overnight hospital stay in the past 12 months. | The number of hospital stays reported in the past 12 months. |
| Note: ^1 4^ The unit of measurement for outpatient care in HRS and KLoSA is two years. ^2 5^ The unit of measurement for inpatient care in HRS and KLoSA is two years. ^3^ The unit of measurement for outpatient care in CHARLS is one month. | | | | |

Table C. The definition of health insurance.

| **Dataset** | **Health insurance** |
| --- | --- |
| **HRS** | Whether the respondent is covered by government health insurance Medicare, Medicaid, private health insurance or other health insurance. |
| **CHARLS** | Whether the respondent is covered by any public health insurance program, private health insurance program, or any other health insurance program. |
| **JSTAR** | **-** |
| **KLoSA** | Whether the respondent is covered by a government health insurance program or private health insurance to compensate for hospital expenses. |
| **MAHS** | Whether the respondent is covered by a government health insurance program, private medical health insurance, or health insurance as a current or former worker. |
| **SHARE** | Whether the respondent is covered by a government health insurance program, such as the National Health Insurance System. |
| *Note:* for JSTAR, the health insurance data is not available. | |

Table D. The definition of lifestyles.

| **Dataset** | Alcohol use | Tobacco use | Physical exercise |
| --- | --- | --- | --- |
| **HRS** | Whether the respondent has ever consumed alcoholic beverages. | Whether the respondent currently smokes. | The frequency of moderately energetic physical activity (hardly ever or never = 0). |
| **SHARE** | Whether the respondent consumes alcohol weekly or has had an alcoholic drink in the last 7 days, depending on the wave. | Whether the respondent currently smokes cigarettes, pipes, or cigars. | The frequency of vigorous and moderately energetic physical activity (hardly ever or never = 0). |
| **KLoSA** | Whether the respondent currently drinks alcoholic beverages. | Whether the respondent currently smokes. | Whether the respondent exercises more than once a week. |
| **MHAS** | Whether the respondent has ever consumed alcoholic beverages. | Whether the respondent currently smokes. | Whether the respondent has engaged in hard physical work three or more times a week on average over the past two years. |
| **CHARLS** | Whether the respondent has consumed alcoholic beverages in the past. | Whether the respondent currently smokes, only asked if they report ever smoking. | Whether the respondent engages in moderately energetic physical activity for at least 10 minutes every week. |
| **JSTAR** | whether the respondent has had an alcoholic drink during the last 6 months | whether the respondent reports ever having smoked | the number of minutes of exercise per day on weekdays |

Table E. Basic demographics by study.

| Variable | HRS | CHARLS | JSTAR | KLoSA | MHAS | SHARE |
| --- | --- | --- | --- | --- | --- | --- |
| Gender, n (%) |  |  |  |  |  |  |
| Male | 53609 (41.78) | 30673 (49.04) | 6378 (49.41) | 25876 (42.93) | 32204 (44.41) | 136173 (43.75) |
| Female | 74695 (58.22) | 31874 (50.96) | 6530 (50.59) | 34397 (57.07) | 40313 (55.59) | 175099 (56.25) |
| Missing values, n (%) | 0 (0) | 0 (0) | 0 (0) | 0 (0) | 0 (0) | 0 (0) |
| Age, mean (SD) | 67.79 (10.92) | 62.8 (9.01) | 64.31 (7.24) | 67.11 (10.31) | 64.86 (10.13) | 67.66 (9.96) |
| Missing values, n (%) | 0 (0) | 0 (0) | 664 (5.14) | 0 (0) | 1280 (1.77) | 20 (.01) |
| Education attainment, n (%) |  |  |  |  |  |  |
| Middle school or below | 30267 (23.59) | 55002 (87.98) | 3784 (29.47) | 9559 (15.86) | 62589 (87.13) | 122466 (39.34) |
| High school | 36020 (28.08) | 4814 (7.7) | 5541 (43.16) | 16593 (27.53) | 2044 (2.85) | 106282 (34.14) |
| College or above | 61993 (48.33) | 2703 (4.32) | 3514 (27.37) | 34119 (56.61) | 7203 (10.03) | 82524 (26.51) |
| Missing values, n (%) | 24 (.02) | 0 (0) | 69 (.53) | 2 (0) | 681 (.94) | 0 (0) |
| Marital Status |  |  |  |  |  |  |
| Not married | 55519 (43.32) | 9556 (15.29) | 2642 (20.48) | 14605 (24.23) | 27558 (38.67) | 101838 (32.75) |
| Married | 72647 (56.68) | 52922 (84.71) | 10258 (79.52) | 45667 (75.77) | 43710 (61.33) | 209128 (67.25) |
| Missing values, n (%) | 123 (.1) | 0 (0) | 8 (.06) | 1 (0) | 1249 (1.72) | 306 (.1) |
| Work status, n (%) |  |  |  |  |  |  |
| No | 79766 (62.44) | 24492 (40.01) | 5987 (46.92) | 36908 (61.23) | 42752 (60.21) | 214974 (70.01) |
| Yes | 47987 (37.56) | 36717 (59.99) | 6772 (53.08) | 23365 (38.77) | 28252 (39.79) | 92089 (29.99) |
| Missing values, n (%) | 545 (.42) | 0 (0) | 149 (1.15) | 0 (0) | 1513 (2.09) | 4209 (1.35) |
| Household Wealth |  |  |  |  |  |  |
| Q1 | 31641 (24.66) | 10439 (26.51) | 2049 (25.2) | 15881 (26.71) | 18054 (24.94) | 62297 (25.02) |
| Q2 | 31829 (24.81) | 10195 (25.89) | 2037 (25.06) | 14053 (23.63) | 18040 (24.92) | 62312 (25.03) |
| Q3 | 32281 (25.16) | 9503 (24.13) | 2027 (24.93) | 14831 (24.94) | 18132 (25.05) | 62256 (25.01) |
| Q4 | 32553 (25.37) | 9245 (23.48) | 2017 (24.81) | 14700 (24.72) | 18154 (25.08) | 62107 (24.95) |
| Missing values, n (%) | 0 (0) | 23173 (37.04) | 4778 (37.02) | 808 (1.34) | 137 (.19) | 62300 (20.01) |
| Alcohol use, n (%) |  |  |  |  |  |  |
| No | 58194 (45.38) | 34115 (55.01) | 5169 (55.71) | 39804 (66.04) | 52763 (74.07) | 127519 (51.45) |
| Yes | 70031 (54.62) | 27898 (44.99) | 4109 (44.29) | 20468 (33.96) | 18475 (25.93) | 120333 (48.55) |
| Missing values, n (%) | 66 (.05) | 542 (.87) | 3630 (28.12) | 1 (0) | 1279 (1.76) | 63420 (20.37) |
| Tobacco use, n (%) |  |  |  |  |  |  |
| No | 110269 (86.5) | 42944 (73.44) | 9513 (80.26) | 51785 (85.92) | 61563 (86.46) | 156513 (82.82) |
| Yes | 17212 (13.5) | 15530 (26.56) | 2339 (19.74) | 8486 (14.08) | 9638 (13.54) | 32459 (17.18) |
| Missing values, n (%) | 805 (.63) | 4081 (6.52) | 1056 (8.18) | 2 (0) | 1316 (1.81) | 63269 (20.33) |
| Physical exercise, n (%) |  |  |  |  |  |  |
| No | 29825 (23.3) | 17436 (49.57) | 692 (5.36) | 38974 (64.66) | 41888 (63.87) | 34131 (13.77) |
| Yes | 98180 (76.7) | 17740 (50.43) | 12216 (94.64) | 21299 (35.34) | 23695 (36.13) | 213733 (86.23) |
| Missing values, n (%) | 287 (.22) | 27379 (43.77) | 0 (0) | 0 (0) | 6934 (9.56) | 63408 (20.37) |
| BMI, n (%) |  |  |  |  |  |  |
| Underweight | 441 (1.02) | 2413 (7.03) | 634 (5.06) | 2308 (3.92) | 85 (1.36) | 3752 (1.24) |
| Normal weight | 8802 (20.32) | 13760 (40.1) | 8833 (70.52) | 25631 (43.52) | 1655 (26.57) | 105133 (34.88) |
| Overweight | 34081 (78.67) | 18140 (52.87) | 3059 (24.42) | 30959 (52.56) | 4490 (72.07) | 192523 (63.87) |
| Missing values, n (%) | 81595 (63.6) | 28242 (45.15) | 382 (2.96) | 1375 (2.28) | 66287 (91.41) | 9517 (3.06) |
| Multimorbidity, n (%) |  |  |  |  |  |  |
| None | 16568 (12.91) | 23839 (38.11) | 5095 (40.45) | 6858 (12.75) | 13940 (19.56) | 53553 (21.51) |
| Phy | 84601 (65.94) | 7071 (11.3) | 4818 (38.25) | 6417 (11.93) | 22296 (31.28) | 122032 (49) |
| Psy | 2137 (1.67) | 14700 (23.5) | 1101 (8.74) | 11244 (20.91) | 4753 (6.67) | 10433 (4.19) |
| Cog | 83 (.06) | 4588 (7.33) | 95 (.75) | 794 (1.48) | 2657 (3.73) | 1028 (.41) |
| Phy + Psy | 22994 (17.92) | 6538 (10.45) | 1295 (10.28) | 14124 (26.26) | 15061 (21.13) | 53398 (21.44) |
| Phy + Cog | 1312 (1.02) | 1205 (1.93) | 111 (.88) | 1638 (3.05) | 5296 (7.43) | 3264 (1.31) |
| Psy + Cog | 31 (.02) | 3250 (5.2) | 27 (.21) | 3235 (6.01) | 1284 (1.8) | 606 (.24) |
| Phy + Psy + Cog | 578 (.45) | 1364 (2.18) | 54 (.43) | 9475 (17.62) | 5984 (8.4) | 4708 (1.89) |
| Outpatient, n (%) |  |  |  |  |  |  |
| No | 12187 (9.56) | 50252 (81.15) | 3345 (30.08) | 10313 (20.31) | 20192 (28.43) | 33680 (10.98) |
| Yes | 115314 (90.44) | 11671 (18.85) | 7775 (69.92) | 40471 (79.69) | 50836 (71.57) | 273142 (89.02) |
| Missing values, n (%) | 0 (0) | 632 (1.01) | 273 (2.11) | 8569 (14.22) | 1489 (2.05) | 4450 (1.43) |
| Inpatient, n (%) |  |  |  |  |  |  |
| No | 94459 (74.18) | 52810 (85.15) | 10093 (90.61) | 44784 (88.03) | 62414 (87.75) | 262035 (84.54) |
| Yes | 32871 (25.82) | 9212 (14.85) | 1046 (9.39) | 6090 (11.97) | 8713 (12.25) | 47925 (15.46) |
| Missing values, n (%) | 0 (0) | 533 (.85) | 254 (1.97) | 8479 (14.07) | 1390 (1.92) | 1312 (.42) |
| Number of outpatient visits, mean (SD) | 3 (1, 5) | 0 (0, 0) | 0 (0, 2) | 4 (1, 8.5) | 3 (0, 11) | 4 (2, 8) |
| Missing values, n (%) | 9647 (7.52) | 1106 (1.77) | 0 (0) | 9489 (15.74) | 1489 (2.05) | 4450 (1.43) |
| Number of inpatient admissions, mean (SD) | 0 (0, 1) | 0 (0, 0) | 0 (0, 0) | 0 (0, 0) | - | 0 (0, 0) |
| Missing values, n (%) | 0 (0) | 559 (.89) | 1792 (13.88) | 8479 (14.07) | - | 1464 (.47) |
| Follow-up waves |  |  |  |  |  |  |
| 1 | 63 (0.15) | 958 (3.77) |  |  | 41 (0.15) | 26 (0.02) |
| 2 | 65 (0.15) | 3807 (14.99) | 1 (0.01) |  | 216 (0.81) | 90 (0.06) |
| >=3 | 42263 (99.7) | 20627 (81.23) | 7119 (99.99) | 11174 (100) | 26557 (99.04) | 139504 (99.92) |
| *Note:* Cog: cognitive disorder; Psy: psychological disorder; Phy: physical condition; Psy + Cog: psychological-cognitive multimorbidity; Phy + Cog: physical-cognitive multimorbidity; Phy + Psy: physical- psychological multimorbidity; Phy + Psy + Cog: physical-psychological-cognitive multimorbidity. HRS: Health and Retirement Study; CHARLS: China Health and Retirement Longitudinal Study; JSTAR: Japanese Study of Aging and Retirement; KLoSA: Korean Longitudinal Study of Aging; MHAS: Mexican Health and Aging Study; SHARE: Survey of Health, Ageing and Retirement in Europe. | | | | | | |

Table F. Basic demographics by country.

| Variable | United States | China | Japan | South Korea | Mexico | Austria | Germany | Sweden | Netherlands | Spain |
| --- | --- | --- | --- | --- | --- | --- | --- | --- | --- | --- |
| Gender, n (%) |  |  |  |  |  |  |  |  |  |  |
| Male | 53609 (41.78) | 30673 (49.04) | 6378 (49.41) | 25876 (42.93) | 32204 (44.41) | 7189 (41.85) | 8616 (47.37) | 7343 (46.22) | 3959 (44.99) | 10176 (45.13) |
| Female | 74695 (58.22) | 31874 (50.96) | 6530 (50.59) | 34397 (57.07) | 40313 (55.59) | 9987 (58.15) | 9574 (52.63) | 8543 (53.78) | 4840 (55.01) | 12370 (54.87) |
| Missing values, n (%) | 0 (0) | 0 (0) | 0 (0) | 0 (0) | 0 (0) | 0 (0) | 0 (0) | 0 (0) | 0 (0) | 0 (0) |
| Age, mean (SD) | 67.79 (10.92) | 62.8 (9.01) | 64.31 (7.24) | 67.11 (10.31) | 64.86 (10.13) | 68.15 (9.76) | 66.66 (9.68) | 70.25 (9.18) | 66.92 (9.58) | 69.64 (10.71) |
| Missing values, n (%) | 0 (0) | 0 (0) | 664 (5.14) | 0 (0) | 1280 (1.77) | 0 (0) | 0 (0) | 1 (.01) | 0 (0) | 0 (0) |
| Education attainment, n (%) |  |  |  |  |  |  |  |  |  |  |
| Middle school or below | 30267 (23.59) | 55002 (87.98) | 3784 (29.47) | 9559 (15.86) | 62589 (87.13) | 4175 (24.31) | 2142 (11.78) | 5789 (36.44) | 3998 (45.44) | 17970 (79.7) |
| High school | 36020 (28.08) | 4814 (7.7) | 5541 (43.16) | 16593 (27.53) | 2044 (2.85) | 7830 (45.59) | 9651 (53.06) | 3674 (23.13) | 2273 (25.83) | 1983 (8.8) |
| College or above | 61993 (48.33) | 2703 (4.32) | 3514 (27.37) | 34119 (56.61) | 7203 (10.03) | 5171 (30.11) | 6397 (35.17) | 6423 (40.43) | 2528 (28.73) | 2593 (11.5) |
| Missing values, n (%) | 24 (.02) | 0 (0) | 69 (.53) | 2 (0) | 681 (.94) | 0 (0) | 0 (0) | 0 (0) | 0 (0) | 0 (0) |
| Marital Status |  |  |  |  |  |  |  |  |  |  |
| Not married | 55519 (43.32) | 9556 (15.29) | 2642 (20.48) | 14605 (24.23) | 27558 (38.67) | 6748 (39.36) | 5073 (27.91) | 5379 (33.91) | 2483 (28.23) | 6001 (26.71) |
| Married | 72647 (56.68) | 52922 (84.71) | 10258 (79.52) | 45667 (75.77) | 43710 (61.33) | 10397 (60.64) | 13105 (72.09) | 10484 (66.09) | 6312 (71.77) | 16469 (73.29) |
| Missing values, n (%) | 123 (.1) | 0 (0) | 8 (.06) | 1 (0) | 1249 (1.72) | 31 (.18) | 12 (.07) | 23 (.14) | 4 (.05) | 76 (.34) |
| Work status, n (%) |  |  |  |  |  |  |  |  |  |  |
| No | 79766 (62.44) | 24492 (40.01) | 5987 (46.92) | 36908 (61.23) | 42752 (60.21) | 13081 (77.51) | 10847 (60.35) | 9503 (60.74) | 5479 (63.29) | 17367 (78.06) |
| Yes | 47987 (37.56) | 36717 (59.99) | 6772 (53.08) | 23365 (38.77) | 28252 (39.79) | 3795 (22.49) | 7127 (39.65) | 6143 (39.26) | 3178 (36.71) | 4881 (21.94) |
| Missing values, n (%) | 545 (.42) | 0 (0) | 149 (1.15) | 0 (0) | 1513 (2.09) | 300 (1.75) | 216 (1.19) | 240 (1.51) | 142 (1.61) | 298 (1.32) |
| Household Wealth |  |  |  |  |  |  |  |  |  |  |
| Q1 | 31641 (24.66) | 10439 (26.51) | 2049 (25.2) | 15881 (26.71) | 18054 (24.94) | 3633 (25.04) | 3810 (25) | 3440 (24.97) | 2207 (25.08) | 4788 (24.98) |
| Q2 | 31829 (24.81) | 10195 (25.89) | 2037 (25.06) | 14053 (23.63) | 18040 (24.92) | 3647 (25.13) | 3802 (24.94) | 3441 (24.98) | 2196 (24.96) | 4806 (25.07) |
| Q3 | 32281 (25.16) | 9503 (24.13) | 2027 (24.93) | 14831 (24.94) | 18132 (25.05) | 3633 (25.04) | 3815 (25.03) | 3445 (25.01) | 2202 (25.03) | 4789 (24.98) |
| Q4 | 32553 (25.37) | 9245 (23.48) | 2017 (24.81) | 14700 (24.72) | 18154 (25.08) | 3597 (24.79) | 3815 (25.03) | 3449 (25.04) | 2194 (24.93) | 4785 (24.96) |
| Missing values, n (%) | 0 (0) | 23173 (37.04) | 4778 (37.02) | 808 (1.34) | 137 (.19) | 2666 (15.52) | 2948 (16.21) | 2111 (13.29) | 0 (0) | 3378 (14.98) |
| Alcohol use, n (%) |  |  |  |  |  |  |  |  |  |  |
| No | 58194 (45.38) | 34115 (55.01) | 5169 (55.71) | 39804 (66.04) | 52763 (74.07) | 6707 (46.58) | 6616 (43.54) | 5525 (40.25) | 3089 (35.3) | 12328 (64.54) |
| Yes | 70031 (54.62) | 27898 (44.99) | 4109 (44.29) | 20468 (33.96) | 18475 (25.93) | 7693 (53.42) | 8580 (56.46) | 8201 (59.75) | 5661 (64.7) | 6774 (35.46) |
| Missing values, n (%) | 66 (.05) | 542 (.87) | 3630 (28.12) | 1 (0) | 1279 (1.76) | 2776 (16.16) | 2994 (16.46) | 2160 (13.6) | 49 (.56) | 3444 (15.28) |
| Tobacco use, n (%) |  |  |  |  |  |  |  |  |  |  |
| No | 110269 (86.5) | 42944 (73.44) | 9513 (80.26) | 51785 (85.92) | 61563 (86.46) | 9018 (81.48) | 8774 (82.02) | 8467 (88.62) | 7352 (84) | 11779 (87.4) |
| Yes | 17212 (13.5) | 15530 (26.56) | 2339 (19.74) | 8486 (14.08) | 9638 (13.54) | 2050 (18.52) | 1923 (17.98) | 1087 (11.38) | 1400 (16) | 1698 (12.6) |
| Missing values, n (%) | 805 (.63) | 4081 (6.52) | 1056 (8.18) | 2 (0) | 1316 (1.81) | 2772 (16.14) | 2988 (16.43) | 2149 (13.53) | 47 (.53) | 3432 (15.22) |
| Physical exercise, n (%) |  |  |  |  |  |  |  |  |  |  |
| No | 29825 (23.3) | 17436 (49.57) | 692 (5.36) | 38974 (64.66) | 41888 (63.87) | 1643 (11.41) | 1412 (9.29) | 871 (6.35) | 809 (9.24) | 3847 (20.14) |
| Yes | 98180 (76.7) | 17740 (50.43) | 12216 (94.64) | 21299 (35.34) | 23695 (36.13) | 12759 (88.59) | 13783 (90.71) | 12856 (93.65) | 7944 (90.76) | 15255 (79.86) |
| Missing values, n (%) | 287 (.22) | 27379 (43.77) | 0 (0) | 0 (0) | 6934 (9.56) | 2774 (16.15) | 2995 (16.47) | 2159 (13.59) | 46 (.52) | 3444 (15.28) |
| BMI, n (%) |  |  |  |  |  |  |  |  |  |  |
| Underweight | 441 (1.02) | 2413 (7.03) | 634 (5.06) | 2308 (3.92) | 85 (1.36) | 244 (1.45) | 175 (.98) | 179 (1.15) | 76 (.89) | 197 (.95) |
| Normal weight | 8802 (20.32) | 13760 (40.1) | 8833 (70.52) | 25631 (43.52) | 1655 (26.57) | 6318 (37.56) | 6373 (35.55) | 6725 (43.33) | 3559 (41.55) | 6679 (32.15) |
| Overweight | 34081 (78.67) | 18140 (52.87) | 3059 (24.42) | 30959 (52.56) | 4490 (72.07) | 10259 (60.99) | 11379 (63.47) | 8617 (55.52) | 4930 (57.56) | 13900 (66.9) |
| Missing values, n (%) | 81595 (63.6) | 28242 (45.15) | 382 (2.96) | 1375 (2.28) | 66287 (91.41) | 325 (1.89) | 251 (1.38) | 361 (2.27) | 222 (2.52) | 1721 (7.63) |
| Multimorbidity, n (%) |  |  |  |  |  |  |  |  |  |  |
| None | 16568 (12.91) | 23839 (38.11) | 5095 (40.45) | 6858 (12.75) | 13940 (19.56) | 3786 (26.09) | 3054 (20.04) | 3343 (24.27) | 2659 (30.22) | 3755 (19.59) |
| Phy | 84601 (65.94) | 7071 (11.3) | 4818 (38.25) | 6417 (11.93) | 22296 (31.28) | 7571 (52.18) | 8347 (54.76) | 7667 (55.66) | 4422 (50.26) | 8583 (44.78) |
| Psy | 2137 (1.67) | 14700 (23.5) | 1101 (8.74) | 11244 (20.91) | 4753 (6.67) | 551 (3.8) | 578 (3.79) | 548 (3.98) | 411 (4.67) | 631 (3.29) |
| Cog | 83 (.06) | 4588 (7.33) | 95 (.75) | 794 (1.48) | 2657 (3.73) | 40 (.28) | 27 (.18) | 27 (.2) | 27 (.31) | 174 (.91) |
| Phy + Psy | 22994 (17.92) | 6538 (10.45) | 1295 (10.28) | 14124 (26.26) | 15061 (21.13) | 2342 (16.14) | 2998 (19.67) | 2036 (14.78) | 1145 (13.01) | 4328 (22.58) |
| Phy + Cog | 1312 (1.02) | 1205 (1.93) | 111 (.88) | 1638 (3.05) | 5296 (7.43) | 92 (.63) | 111 (.73) | 83 (.6) | 76 (.86) | 626 (3.27) |
| Psy + Cog | 31 (.02) | 3250 (5.2) | 27 (.21) | 3235 (6.01) | 1284 (1.8) | 21 (.14) | 19 (.12) | 7 (.05) | 12 (.14) | 96 (.5) |
| Phy + Psy + Cog | 578 (.45) | 1364 (2.18) | 54 (.43) | 9475 (17.62) | 5984 (8.4) | 107 (.74) | 108 (.71) | 64 (.46) | 47 (.53) | 975 (5.09) |
| Outpatient, n (%) |  |  |  |  |  |  |  |  |  |  |
| No | 12187 (9.56) | 50252 (81.15) | 3345 (30.08) | 10313 (20.31) | 20192 (28.43) | 1471 (8.66) | 1008 (5.57) | 2386 (15.23) | 1228 (14.09) | 1943 (8.76) |
| Yes | 115314 (90.44) | 11671 (18.85) | 7775 (69.92) | 40471 (79.69) | 50836 (71.57) | 15508 (91.34) | 17083 (94.43) | 13279 (84.77) | 7488 (85.91) | 20234 (91.24) |
| Missing values, n (%) | 0 (0) | 632 (1.01) | 273 (2.11) | 8569 (14.22) | 1489 (2.05) | 197 (1.15) | 99 (.54) | 221 (1.39) | 83 (.94) | 369 (1.64) |
| Inpatient, n (%) |  |  |  |  |  |  |  |  |  |  |
| No | 94459 (74.18) | 52810 (85.15) | 10093 (90.61) | 44784 (88.03) | 62414 (87.75) | 13011 (76.11) | 14345 (79.11) | 13576 (85.67) | 7732 (88.22) | 19686 (87.65) |
| Yes | 32871 (25.82) | 9212 (14.85) | 1046 (9.39) | 6090 (11.97) | 8713 (12.25) | 4085 (23.89) | 3788 (20.89) | 2271 (14.33) | 1032 (11.78) | 2774 (12.35) |
| Missing values, n (%) | 0 (0) | 533 (.85) | 254 (1.97) | 8479 (14.07) | 1390 (1.92) | 80 (.47) | 57 (.31) | 39 (.25) | 35 (.4) | 86 (.38) |
| Number of outpatient visits, mean (SD) | 4.97 (10.46) | 5.11 (17.35) | 10.39 (26.75) | 6.53 (10.97) | 5.3 (7.87) | 7.97 (10.88) | 8.45 (11.07) | 4.61 (8.49) | 6.07 (10.04) | 6.16 (8.33) |
| Missing values, n (%) | 9647 (7.52) | 1106 (1.77) | 0 (0) | 9489 (15.74) | 1489 (2.05) | 197 (1.15) | 99 (.54) | 221 (1.39) | 83 (.94) | 369 (1.64) |
| Number of inpatient admissions, mean (SD) | .5 (1.6) | .46 (1.5) | .27 (1.2) | .15 (.5) | - | .8 (1.97) | .67 (1.77) | .48 (1.64) | .37 (1.38) | .43 (1.63) |
| Missing values, n (%) | 0 (0) | 559 (.89) | 1792 (13.88) | 8479 (14.07) | - | 88 (.51) | 60 (.33) | 49 (.31) | 35 (.4) | 98 (.43) |

Table F. Basic demographics by country (Continued).

| Variable | Italy | France | Denmark | Greece | Switzerland | Belgium | Israel | Czech Republic | Poland | Luxembourg | Hungary |
| --- | --- | --- | --- | --- | --- | --- | --- | --- | --- | --- | --- |
| Gender, n (%) |  |  |  |  |  |  |  |  |  |  |  |
| Male | 9095 (45.41) | 8448 (42.9) | 7089 (46.28) | 4684 (43.25) | 6238 (45.53) | 10443 (44.95) | 3269 (42.9) | 9240 (40.93) | 4540 (44.41) | 2449 (46.09) | 2223 (41.94) |
| Female | 10933 (54.59) | 11244 (57.1) | 8227 (53.72) | 6147 (56.75) | 7463 (54.47) | 12791 (55.05) | 4351 (57.1) | 13337 (59.07) | 5684 (55.59) | 2864 (53.91) | 3077 (58.06) |
| Missing values, n (%) | 0 (0) | 0 (0) | 0 (0) | 0 (0) | 0 (0) | 0 (0) | 0 (0) | 0 (0) | 0 (0) | 0 (0) | 0 (0) |
| Age, mean (SD) | 67.64 (9.82) | 67.79 (10.6) | 66.06 (10.06) | 68.41 (9.9) | 67.73 (9.88) | 66.48 (10.57) | 69.7 (9.89) | 67.81 (9.04) | 66.5 (9.67) | 65.71 (9.44) | 66.67 (9.09) |
| Missing values, n (%) | 4 (.02) | 0 (0) | 0 (0) | 1 (.01) | 0 (0) | 0 (0) | 1 (.01) | 5 (.02) | 0 (0) | 1 (.02) | 1 (.02) |
| Education attainment, n (%) |  |  |  |  |  |  |  |  |  |  |  |
| Middle school or below | 13987 (69.84) | 8297 (42.13) | 2717 (17.74) | 5658 (52.24) | 2737 (19.98) | 9150 (39.38) | 2573 (33.77) | 9207 (40.78) | 3351 (32.78) | 2328 (43.82) | 1580 (29.81) |
| High school | 3897 (19.46) | 7037 (35.74) | 5950 (38.85) | 2788 (25.74) | 6362 (46.43) | 6161 (26.52) | 1896 (24.88) | 9762 (43.24) | 5270 (51.55) | 1827 (34.39) | 2499 (47.15) |
| College or above | 2144 (10.71) | 4358 (22.13) | 6649 (43.41) | 2385 (22.02) | 4602 (33.59) | 7923 (34.1) | 3151 (41.35) | 3608 (15.98) | 1603 (15.68) | 1158 (21.8) | 1221 (23.04) |
| Missing values, n (%) | 0 (0) | 0 (0) | 0 (0) | 0 (0) | 0 (0) | 0 (0) | 0 (0) | 0 (0) | 0 (0) | 0 (0) | 0 (0) |
| Marital Status |  |  |  |  |  |  |  |  |  |  |  |
| Not married | 4841 (24.19) | 7514 (38.23) | 4867 (31.78) | 2989 (27.6) | 4574 (33.4) | 8749 (37.67) | 2199 (29.03) | 8360 (37.07) | 3016 (29.51) | 1328 (25) | 1938 (36.57) |
| Married | 15172 (75.81) | 12141 (61.77) | 10449 (68.22) | 7842 (72.4) | 9121 (66.6) | 14476 (62.33) | 5376 (70.97) | 14194 (62.93) | 7204 (70.49) | 3985 (75) | 3362 (63.43) |
| Missing values, n (%) | 15 (.07) | 37 (.19) | 0 (0) | 0 (0) | 6 (.04) | 9 (.04) | 45 (.59) | 23 (.1) | 4 (.04) | 0 (0) | 0 (0) |
| Work status, n (%) |  |  |  |  |  |  |  |  |  |  |  |
| No | 15252 (76.68) | 13891 (71.88) | 7792 (51.68) | 8297 (76.9) | 7502 (55.65) | 15625 (69.05) | 4801 (64.35) | 15947 (71.79) | 7589 (74.69) | 3907 (75.15) | 4140 (78.93) |
| Yes | 4639 (23.32) | 5434 (28.12) | 7284 (48.32) | 2493 (23.1) | 5978 (44.35) | 7004 (30.95) | 2660 (35.65) | 6266 (28.21) | 2571 (25.31) | 1292 (24.85) | 1105 (21.07) |
| Missing values, n (%) | 137 (.68) | 367 (1.86) | 240 (1.57) | 41 (.38) | 221 (1.61) | 605 (2.6) | 159 (2.09) | 364 (1.61) | 64 (.63) | 114 (2.15) | 55 (1.04) |
| Household Wealth |  |  |  |  |  |  |  |  |  |  |  |
| Q1 | 4271 (24.99) | 4338 (24.8) | 3354 (25.05) | 2442 (25.17) | 3019 (25.02) | 4982 (24.96) | 1377 (25) | 4837 (25.04) | 1693 (25.15) | 1011 (24.78) | 948 (25.15) |
| Q2 | 4289 (25.1) | 4380 (25.04) | 3347 (25) | 2417 (24.91) | 3019 (25.02) | 4996 (25.03) | 1370 (24.88) | 4849 (25.1) | 1681 (24.97) | 1024 (25.1) | 943 (25.01) |
| Q3 | 4263 (24.95) | 4391 (25.1) | 3352 (25.03) | 2429 (25.03) | 3010 (24.94) | 5006 (25.08) | 1376 (24.99) | 4814 (24.92) | 1682 (24.99) | 1026 (25.15) | 943 (25.01) |
| Q4 | 4265 (24.96) | 4383 (25.06) | 3337 (24.92) | 2415 (24.89) | 3019 (25.02) | 4976 (24.93) | 1384 (25.13) | 4818 (24.94) | 1675 (24.88) | 1019 (24.98) | 936 (24.83) |
| Missing values, n (%) | 2940 (14.68) | 2200 (11.17) | 1926 (12.58) | 1128 (10.41) | 1634 (11.93) | 3274 (14.09) | 2113 (27.73) | 3259 (14.44) | 3493 (34.16) | 1233 (23.21) | 1530 (28.87) |
| Alcohol use, n (%) |  |  |  |  |  |  |  |  |  |  |  |
| No | 9989 (58.58) | 6612 (38.02) | 3461 (25.95) | 5071 (52.35) | 3742 (31.16) | 7164 (36.17) | 4655 (85.12) | 10836 (56.37) | 5084 (75.74) | 1446 (35.55) | 2728 (73.1) |
| Yes | 7064 (41.42) | 10777 (61.98) | 9878 (74.05) | 4616 (47.65) | 8268 (68.84) | 12641 (63.83) | 814 (14.88) | 8387 (43.63) | 1628 (24.26) | 2621 (64.45) | 1004 (26.9) |
| Missing values, n (%) | 2975 (14.85) | 2303 (11.7) | 1977 (12.91) | 1144 (10.56) | 1691 (12.34) | 3429 (14.76) | 2151 (28.23) | 3354 (14.86) | 3512 (34.35) | 1246 (23.45) | 1568 (29.58) |
| Tobacco use, n (%) |  |  |  |  |  |  |  |  |  |  |  |
| No | 10735 (84.61) | 11522 (84.97) | 7560 (80.2) | 5500 (77.05) | 7329 (80.92) | 12078 (82.17) | 3118 (87.22) | 11255 (78.3) | 3804 (76.77) | 2533 (84.91) | 2946 (78.9) |
| Yes | 1952 (15.39) | 2038 (15.03) | 1866 (19.8) | 1638 (22.95) | 1728 (19.08) | 2620 (17.83) | 457 (12.78) | 3120 (21.7) | 1151 (23.23) | 450 (15.09) | 788 (21.1) |
| Missing values, n (%) | 2970 (14.83) | 2293 (11.64) | 1973 (12.88) | 1145 (10.57) | 1686 (12.31) | 3416 (14.7) | 2144 (28.14) | 3346 (14.82) | 3504 (34.27) | 1238 (23.3) | 1566 (29.55) |
| Physical exercise, n (%) |  |  |  |  |  |  |  |  |  |  |  |
| No | 4238 (24.85) | 2329 (13.4) | 958 (7.18) | 987 (10.19) | 867 (7.22) | 2770 (13.99) | 1468 (26.91) | 2330 (12.12) | 1421 (21.17) | 406 (9.96) | 650 (17.41) |
| Yes | 12815 (75.15) | 15058 (86.6) | 12378 (92.82) | 8701 (89.81) | 11143 (92.78) | 17034 (86.01) | 3987 (73.09) | 16898 (87.88) | 5290 (78.83) | 3669 (90.04) | 3084 (82.59) |
| Missing values, n (%) | 2975 (14.85) | 2305 (11.71) | 1980 (12.93) | 1143 (10.55) | 1691 (12.34) | 3430 (14.76) | 2165 (28.41) | 3349 (14.83) | 3513 (34.36) | 1238 (23.3) | 1566 (29.55) |
| BMI, n (%) |  |  |  |  |  |  |  |  |  |  |  |
| Underweight | 259 (1.32) | 397 (2.07) | 293 (1.96) | 45 (.42) | 303 (2.25) | 433 (1.91) | 69 (1.01) | 147 (.67) | 104 (1.06) | 88 (1.7) | 64 (1.24) |
| Normal weight | 7798 (39.62) | 7755 (40.46) | 6418 (42.84) | 3053 (28.67) | 6189 (45.92) | 8755 (38.71) | 2409 (35.25) | 5549 (25.35) | 2851 (29.16) | 1865 (35.93) | 1525 (29.46) |
| Overweight | 11625 (59.06) | 11015 (57.47) | 8272 (55.21) | 7549 (70.9) | 6987 (51.84) | 13428 (59.37) | 4356 (63.74) | 16191 (73.98) | 6821 (69.77) | 3237 (62.37) | 3587 (69.3) |
| Missing values, n (%) | 337 (1.68) | 503 (2.55) | 325 (2.12) | 182 (1.68) | 214 (1.56) | 602 (2.59) | 753 (9.88) | 648 (2.87) | 446 (4.36) | 120 (2.26) | 108 (2.04) |
| Multimorbidity, n (%) |  |  |  |  |  |  |  |  |  |  |  |
| None | 3389 (19.83) | 3277 (18.68) | 3324 (24.82) | 2321 (23.92) | 3729 (30.9) | 4090 (20.49) | 1252 (22.73) | 3320 (17.19) | 886 (13.16) | 884 (21.67) | 653 (17.32) |
| Phy | 7353 (43.03) | 7988 (45.54) | 7676 (57.33) | 4420 (45.55) | 6102 (50.57) | 9702 (48.61) | 2538 (46.09) | 11110 (57.51) | 3028 (44.99) | 1962 (48.09) | 1717 (45.54) |
| Psy | 756 (4.42) | 1030 (5.87) | 397 (2.96) | 455 (4.69) | 506 (4.19) | 955 (4.78) | 197 (3.58) | 531 (2.75) | 262 (3.89) | 183 (4.49) | 135 (3.58) |
| Cog | 105 (.61) | 73 (.42) | 15 (.11) | 101 (1.04) | 25 (.21) | 68 (.34) | 21 (.38) | 29 (.15) | 37 (.55) | 21 (.51) | 15 (.4) |
| Phy + Psy | 4271 (24.99) | 4619 (26.33) | 1839 (13.73) | 1735 (17.88) | 1606 (13.31) | 4718 (23.64) | 1229 (22.32) | 3942 (20.41) | 2113 (31.39) | 931 (22.82) | 1081 (28.67) |
| Phy + Cog | 392 (2.29) | 237 (1.35) | 83 (.62) | 271 (2.79) | 53 (.44) | 199 (1) | 86 (1.56) | 172 (.89) | 140 (2.08) | 49 (1.2) | 43 (1.14) |
| Psy + Cog | 85 (.5) | 49 (.28) | 6 (.04) | 67 (.69) | <5 (-) | 33 (.17) | 12 (.22) | 16 (.08) | 21 (.31) | 10 (.25) | 11 (.29) |
| Phy + Psy + Cog | 738 (4.32) | 268 (1.53) | 50 (.37) | 333 (3.43) | 42 (.35) | 195 (.98) | 172 (3.12) | 198 (1.02) | 244 (3.63) | 40 (.98) | 115 (3.05) |
| Outpatient, n (%) |  |  |  |  |  |  |  |  |  |  |  |
| No | 2233 (11.27) | 1127 (5.8) | 1954 (12.89) | 2037 (19.13) | 1768 (13) | 1374 (5.97) | 688 (9.9) | 1262 (5.67) | 1393 (13.74) | 194 (3.7) | 592 (11.37) |
| Yes | 17576 (88.73) | 18296 (94.2) | 13204 (87.11) | 8613 (80.87) | 11832 (87) | 21623 (94.03) | 6261 (90.1) | 21012 (94.33) | 8744 (86.26) | 5045 (96.3) | 4614 (88.63) |
| Missing values, n (%) | 219 (1.09) | 269 (1.37) | 158 (1.03) | 181 (1.67) | 101 (.74) | 237 (1.02) | 671 (8.81) | 303 (1.34) | 87 (.85) | 74 (1.39) | 94 (1.77) |
| Inpatient, n (%) |  |  |  |  |  |  |  |  |  |  |  |
| No | 17606 (88.19) | 16594 (84.95) | 13306 (87.18) | 10000 (92.61) | 11494 (84.13) | 19148 (82.73) | 6331 (83.79) | 18312 (81.41) | 8303 (81.58) | 4343 (81.93) | 4370 (83.05) |
| Yes | 2357 (11.81) | 2940 (15.05) | 1957 (12.82) | 798 (7.39) | 2168 (15.87) | 3998 (17.27) | 1225 (16.21) | 4182 (18.59) | 1875 (18.42) | 958 (18.07) | 892 (16.95) |
| Missing values, n (%) | 65 (.32) | 158 (.8) | 53 (.35) | 33 (.3) | 39 (.28) | 88 (.38) | 64 (.84) | 83 (.37) | 46 (.45) | 12 (.23) | 38 (.72) |
| Number of outpatient visits, mean (SD) | 8.56 (11.48) | 6.38 (7.46) | 5.19 (8.22) | 4.89 (6.82) | 5.37 (8.8) | 8.54 (11.58) | 7.56 (11.48) | 7.96 (9.49) | 7.02 (7.23) | 8.93 (12.48) | 8.53 (10.47) |
| Missing values, n (%) | 219 (1.09) | 269 (1.37) | 158 (1.03) | 181 (1.67) | 101 (.74) | 237 (1.02) | 671 (8.81) | 303 (1.34) | 87 (.85) | 74 (1.39) | 94 (1.77) |
| Number of inpatient admissions, mean (SD) | .4 (1.51) | .64 (2.31) | .43 (1.5) | .28 (1.41) | .61 (2.1) | .55 (1.65) | .56 (1.75) | .67 (2.07) | .64 (1.89) | .69 (2.23) | .79 (2.69) |
| Missing values, n (%) | 69 (.34) | 175 (.89) | 57 (.37) | 37 (.34) | 42 (.31) | 106 (.46) | 78 (1.02) | 91 (.4) | 48 (.47) | 17 (.32) | 41 (.77) |

Table F. Basic demographics by country (Continued).

| Variable | Portugal | Slovenia | Estonia | Croatia | Lithuania | Bulgaria | Cyprus | Finland | Latvia | Romania | Slovakia |
| --- | --- | --- | --- | --- | --- | --- | --- | --- | --- | --- | --- |
| Gender, n (%) |  |  |  |  |  |  |  |  |  |  |  |
| Male | 2169 (44.4) | 6836 (42.8) | 10252 (39.37) | 2671 (44.47) | 1247 (36.58) | 1190 (41.9) | 702 (40.39) | 1453 (46.5) | 915 (37.04) | 1437 (43.27) | 1386 (46.45) |
| Female | 2716 (55.6) | 9135 (57.2) | 15791 (60.63) | 3335 (55.53) | 2162 (63.42) | 1650 (58.1) | 1036 (59.61) | 1672 (53.5) | 1555 (62.96) | 1884 (56.73) | 1598 (53.55) |
| Missing values, n (%) | 0 (0) | 0 (0) | 0 (0) | 0 (0) | 0 (0) | 0 (0) | 0 (0) | 0 (0) | 0 (0) | 0 (0) | 0 (0) |
| Age, mean (SD) | 66.96 (9.35) | 67.71 (9.81) | 68.24 (10.13) | 66.18 (9.14) | 67.19 (10.5) | 67.11 (9.49) | 70.23 (10.43) | 66.89 (9.56) | 67.41 (10.2) | 66.11 (9.33) | 62.54 (8.13) |
| Missing values, n (%) | 0 (0) | 0 (0) | 0 (0) | 0 (0) | 0 (0) | 4 (.14) | 0 (0) | 0 (0) | 1 (.04) | 0 (0) | 1 (.03) |
| Education attainment, n (%) |  |  |  |  |  |  |  |  |  |  |  |
| Middle school or below | 3911 (80.06) | 5387 (33.73) | 7145 (27.44) | 3535 (58.86) | 669 (19.62) | 900 (31.69) | 951 (54.72) | 897 (28.7) | 447 (18.1) | 1637 (49.29) | 327 (10.96) |
| High school | 444 (9.09) | 7455 (46.68) | 8726 (33.51) | 1497 (24.93) | 1253 (36.76) | 1434 (50.49) | 487 (28.02) | 883 (28.26) | 862 (34.9) | 1247 (37.55) | 2359 (79.05) |
| College or above | 530 (10.85) | 3129 (19.59) | 10172 (39.06) | 974 (16.22) | 1487 (43.62) | 506 (17.82) | 300 (17.26) | 1345 (43.04) | 1161 (47) | 437 (13.16) | 298 (9.99) |
| Missing values, n (%) | 0 (0) | 0 (0) | 0 (0) | 0 (0) | 0 (0) | 0 (0) | 0 (0) | 0 (0) | 0 (0) | 0 (0) | 0 (0) |
| Marital Status |  |  |  |  |  |  |  |  |  |  |  |
| Not married | 1226 (25.1) | 4801 (30.07) | 10959 (42.09) | 1524 (25.39) | 1516 (44.47) | 1025 (36.12) | 443 (25.49) | 984 (31.5) | 1142 (46.23) | 1003 (30.2) | 730 (24.46) |
| Married | 3658 (74.9) | 11166 (69.93) | 15075 (57.91) | 4479 (74.61) | 1893 (55.53) | 1813 (63.88) | 1295 (74.51) | 2140 (68.5) | 1328 (53.77) | 2318 (69.8) | 2254 (75.54) |
| Missing values, n (%) | 1 (.02) | 4 (.03) | 9 (.03) | 3 (.05) | 0 (0) | 2 (.07) | 0 (0) | 1 (.03) | 0 (0) | 0 (0) | 0 (0) |
| Work status, n (%) |  |  |  |  |  |  |  |  |  |  |  |
| No | 3663 (76.36) | 13169 (83.21) | 16588 (64.26) | 4818 (80.74) | 2318 (68.32) | 2085 (73.83) | 1397 (81.51) | 2046 (66.04) | 1734 (70.52) | 2796 (84.42) | 1696 (56.97) |
| Yes | 1134 (23.64) | 2658 (16.79) | 9226 (35.74) | 1149 (19.26) | 1075 (31.68) | 739 (26.17) | 317 (18.49) | 1052 (33.96) | 725 (29.48) | 516 (15.58) | 1281 (43.03) |
| Missing values, n (%) | 88 (1.8) | 144 (.9) | 229 (.88) | 39 (.65) | 16 (.47) | 16 (.56) | 24 (1.38) | 27 (.86) | 11 (.45) | 9 (.27) | 7 (.23) |
| Household Wealth |  |  |  |  |  |  |  |  |  |  |  |
| Q1 | 901 (24.97) | 3080 (25.05) | 5275 (25.14) | 908 (25.01) | 355 (24.96) | 243 (27.06) | 135 (25.09) | 288 (24.85) | 197 (25.22) | 313 (24.72) | 250 (25.35) |
| Q2 | 905 (25.08) | 3066 (24.94) | 5268 (25.1) | 910 (25.07) | 358 (25.18) | 207 (23.05) | 134 (24.91) | 291 (25.11) | 195 (24.97) | 327 (25.83) | 245 (24.85) |
| Q3 | 903 (25.02) | 3079 (25.04) | 5232 (24.93) | 903 (24.88) | 356 (25.04) | 226 (25.17) | 136 (25.28) | 290 (25.02) | 197 (25.22) | 309 (24.41) | 247 (25.05) |
| Q4 | 900 (24.94) | 3070 (24.97) | 5209 (24.82) | 909 (25.04) | 353 (24.82) | 222 (24.72) | 133 (24.72) | 290 (25.02) | 192 (24.58) | 317 (25.04) | 244 (24.75) |
| Missing values, n (%) | 1276 (26.12) | 3676 (23.02) | 5059 (19.43) | 2376 (39.56) | 1987 (58.29) | 1942 (68.38) | 1200 (69.04) | 1966 (62.91) | 1689 (68.38) | 2055 (61.88) | 1998 (66.96) |
| Alcohol use, n (%) |  |  |  |  |  |  |  |  |  |  |  |
| No | 1857 (51.84) | 7055 (57.57) | 16068 (76.89) | 2296 (63.48) | 1023 (71.99) | 602 (67.04) | 349 (65.23) | 599 (51.86) | 577 (74.07) | 891 (70.49) | 578 (58.68) |
| Yes | 1725 (48.16) | 5200 (42.43) | 4830 (23.11) | 1321 (36.52) | 398 (28.01) | 296 (32.96) | 186 (34.77) | 556 (48.14) | 202 (25.93) | 373 (29.51) | 407 (41.32) |
| Missing values, n (%) | 1303 (26.67) | 3716 (23.27) | 5145 (19.76) | 2389 (39.78) | 1988 (58.32) | 1942 (68.38) | 1203 (69.22) | 1970 (63.04) | 1691 (68.46) | 2057 (61.94) | 1999 (66.99) |
| Tobacco use, n (%) |  |  |  |  |  |  |  |  |  |  |  |
| No | 1890 (89.74) | 8320 (86.38) | 13014 (81.12) | 2819 (77.96) | 1202 (84.59) | 740 (82.77) | 466 (87.1) | 1040 (89.97) | 665 (85.15) | 1074 (84.97) | 814 (82.64) |
| Yes | 216 (10.26) | 1312 (13.62) | 3029 (18.88) | 797 (22.04) | 219 (15.41) | 154 (17.23) | 69 (12.9) | 116 (10.03) | 116 (14.85) | 190 (15.03) | 171 (17.36) |
| Missing values, n (%) | 1297 (26.55) | 3712 (23.24) | 5106 (19.61) | 2390 (39.79) | 1988 (58.32) | 1946 (68.52) | 1203 (69.22) | 1969 (63.01) | 1689 (68.38) | 2057 (61.94) | 1999 (66.99) |
| Physical exercise, n (%) |  |  |  |  |  |  |  |  |  |  |  |
| No | 907 (25.32) | 1424 (11.62) | 2994 (14.32) | 382 (10.56) | 264 (18.58) | 192 (21.4) | 226 (42.16) | 64 (5.55) | 111 (14.23) | 206 (16.3) | 91 (9.24) |
| Yes | 2675 (74.68) | 10829 (88.38) | 17919 (85.68) | 3235 (89.44) | 1157 (81.42) | 705 (78.6) | 310 (57.84) | 1090 (94.45) | 669 (85.77) | 1058 (83.7) | 894 (90.76) |
| Missing values, n (%) | 1303 (26.67) | 3718 (23.28) | 5130 (19.7) | 2389 (39.78) | 1988 (58.32) | 1943 (68.42) | 1202 (69.16) | 1971 (63.07) | 1690 (68.42) | 2057 (61.94) | 1999 (66.99) |
| BMI, n (%) |  |  |  |  |  |  |  |  |  |  |  |
| Underweight | 51 (1.1) | 122 (.79) | 279 (1.1) | 39 (.67) | 26 (.77) | 35 (1.29) | 12 (.78) | 21 (.69) | 20 (.83) | 43 (1.33) | 22 (.75) |
| Normal weight | 1500 (32.43) | 4516 (29.14) | 7636 (30.15) | 1626 (27.74) | 920 (27.38) | 836 (30.77) | 492 (32.14) | 1027 (33.53) | 617 (25.63) | 897 (27.8) | 858 (29.26) |
| Overweight | 3074 (66.46) | 10859 (70.07) | 17411 (68.75) | 4196 (71.59) | 2414 (71.85) | 1846 (67.94) | 1027 (67.08) | 2015 (65.79) | 1770 (73.54) | 2287 (70.87) | 2052 (69.99) |
| Missing values, n (%) | 250 (5.12) | 442 (2.77) | 682 (2.62) | 145 (2.41) | 49 (1.44) | 123 (4.33) | 206 (11.85) | 62 (1.98) | 63 (2.55) | 93 (2.8) | 52 (1.74) |
| Multimorbidity, n (%) |  |  |  |  |  |  |  |  |  |  |  |
| None | 661 (18.32) | 3060 (24.89) | 3539 (16.87) | 877 (24.16) | 255 (17.93) | 155 (17.26) | 91 (16.91) | 223 (19.24) | 140 (17.93) | 246 (19.43) | 411 (41.68) |
| Phy | 1269 (35.16) | 6006 (48.85) | 9440 (44.99) | 1591 (43.83) | 589 (41.42) | 0 (0) | 282 (52.42) | 616 (53.15) | 367 (46.99) | 492 (38.86) | 334 (33.87) |
| Psy | 232 (6.43) | 596 (4.85) | 980 (4.67) | 176 (4.85) | 55 (3.87) | 482 (53.67) | 8 (1.49) | 58 (5) | 25 (3.2) | 60 (4.74) | 52 (5.27) |
| Cog | 53 (1.47) | 44 (.36) | 51 (.24) | 24 (.66) | 6 (.42) | 19 (2.12) | 8 (1.49) | <5 (-) | <5 (-) | 20 (1.58) | <5 (-) |
| Phy + Psy | 1039 (28.79) | 2326 (18.92) | 6398 (30.49) | 843 (23.22) | 463 (32.56) | 192 (21.38) | 86 (15.99) | 250 (21.57) | 212 (27.14) | 335 (26.46) | 160 (16.23) |
| Phy + Cog | 105 (2.91) | 102 (.83) | 174 (.83) | 36 (.99) | 17 (1.2) | 17 (1.89) | 34 (6.32) | 6 (.52) | 12 (1.54) | 26 (2.05) | <5 (-) |
| Psy + Cog | 29 (.8) | 20 (.16) | 45 (.21) | 11 (.3) | 6 (.42) | <5 (-) | <5 (-) | <5 (-) | <5 (-) | 15 (1.18) | <5 (-) |
| Phy + Psy + Cog | 221 (6.12) | 141 (1.15) | 357 (1.7) | 72 (1.98) | 31 (2.18) | 28 (3.12) | 27 (5.02) | <5 (-) | 19 (2.43) | 72 (5.69) | 21 (2.13) |
| Outpatient, n (%) |  |  |  |  |  |  |  |  |  |  |  |
| No | 397 (8.42) | 1967 (12.47) | 3722 (14.54) | 965 (16.22) | 482 (14.23) | 524 (18.71) | 359 (20.92) | 479 (15.45) | 413 (16.87) | 946 (28.61) | 408 (13.9) |
| Yes | 4317 (91.58) | 13801 (87.53) | 21882 (85.46) | 4984 (83.78) | 2906 (85.77) | 2277 (81.29) | 1357 (79.08) | 2621 (84.55) | 2035 (83.13) | 2360 (71.39) | 2527 (86.1) |
| Missing values, n (%) | 171 (3.5) | 203 (1.27) | 439 (1.69) | 57 (.95) | 21 (.62) | 39 (1.37) | 22 (1.27) | 25 (.8) | 22 (.89) | 15 (.45) | 49 (1.64) |
| Inpatient, n (%) |  |  |  |  |  |  |  |  |  |  |  |
| No | 4264 (88.06) | 13527 (85.11) | 22036 (85.05) | 5194 (86.73) | 2757 (81.14) | 2448 (86.53) | 1526 (88.67) | 2711 (86.97) | 2132 (86.42) | 2764 (83.4) | 2731 (91.64) |
| Yes | 578 (11.94) | 2367 (14.89) | 3872 (14.95) | 795 (13.27) | 641 (18.86) | 381 (13.47) | 195 (11.33) | 406 (13.03) | 335 (13.58) | 550 (16.6) | 249 (8.36) |
| Missing values, n (%) | 43 (.88) | 77 (.48) | 135 (.52) | 17 (.28) | 11 (.32) | 11 (.39) | 17 (.98) | 8 (.26) | 3 (.12) | 7 (.21) | 4 (.13) |
| Number of outpatient visits, mean (SD) | 5.4 (8.97) | 5.54 (8.7) | 5.62 (7.96) | 7.99 (10.87) | 5.04 (6.38) | 5.29 (5.75) | 5.28 (9.94) | 4.02 (6.98) | 4.67 (6.78) | 4.83 (6.83) | 5.36 (7.41) |
| Missing values, n (%) | 171 (3.5) | 203 (1.27) | 439 (1.69) | 57 (.95) | 21 (.62) | 39 (1.37) | 22 (1.27) | 25 (.8) | 22 (.89) | 15 (.45) | 49 (1.64) |
| Number of inpatient admissions, mean (SD) | .32 (1.09) | .62 (2.28) | .51 (1.7) | .57 (2.25) | .89 (2.87) | .49 (1.65) | .39 (1.53) | .48 (1.81) | .58 (2.17) | .67 (2.16) | .34 (1.61) |
| Missing values, n (%) | 50 (1.02) | 85 (.53) | 151 (.58) | 18 (.3) | 12 (.35) | 11 (.39) | 17 (.98) | 8 (.26) | 4 (.16) | 8 (.24) | 4 (.13) |
| *Note:* Cog: cognitive disorder; Psy: psychological disorder; Phy: physical condition; Psy + Cog: psychological-cognitive multimorbidity; Phy + Cog: physical-cognitive multimorbidity; Phy + Psy: physical- psychological multimorbidity; Phy + Psy + Cog: physical-psychological-cognitive multimorbidity. | | | | | | | | | | | |

Table G. Marginal predicted counts.

| Multimorbidity | Marginal effects (95% CI) |
| --- | --- |
| Phy | 0.69(0.62, 0.76) |
| Psy | 0.33(0.28, 0.39) |
| Cog | -0.07(-0.23, 0.09) |
| Phy + Psy | 0.93(0.87, 1.00) |
| Phy + Cog | 0.66(0.57, 0.75) |
| Psy + Cog | 0.43(0.31, 0.55) |
| Phy + Psy + Cog | 0.83(0.76, 0.90) |
| Phy | 0.86(0.80, 0.92) |
| Psy | 0.59(0.49, 0.70) |
| Cog | 0.37(0.10, 0.63) |
| Phy + Psy | 1.36(1.29, 1.44) |
| Phy + Cog | 0.96(0.84, 1.09) |
| Psy + Cog | 0.81(0.58, 1.04) |
| Phy + Psy + Cog | 1.40(1.28, 1.51) |

Table H. The statistical test results of multimorbidity*SES.

| Outcome | Interaction term | | Measure | Value | P |
| --- | --- | --- | --- | --- | --- |
| Outpatient | Household wealth * | Phy | OR | 1.04 | 0.008 |
|  |  | Psy |  | 0.98 | 0.185 |
|  |  | Cog |  | 0.95 | 0.249 |
|  |  | Phy + Psy |  | 0.97 | 0.092 |
|  |  | Phy + Cog |  | 0.93 | 0.068 |
|  |  | Psy + Cog |  | 0.95 | 0.141 |
|  |  | Phy + Psy + Cog |  | 1.01 | 0.660 |
| Inpatient |  | Phy |  | 0.98 | 0.175 |
|  |  | Psy |  | 0.94 | 0.025 |
|  |  | Cog |  | 1.01 | 0.917 |
|  |  | Phy + Psy |  | 0.97 | 0.085 |
|  |  | Phy + Cog |  | 1.03 | 0.516 |
|  |  | Psy + Cog |  | 0.95 | 0.311 |
|  |  | Phy + Psy + Cog |  | 1.02 | 0.504 |
| Number of outpatient visits |  | Phy | IRR | 0.98 | 0.000 |
|  |  | Psy |  | 0.96 | 0.000 |
|  |  | Cog |  | 0.99 | 0.732 |
|  |  | Phy + Psy |  | 0.96 | 0.000 |
|  |  | Phy + Cog |  | 0.98 | 0.196 |
|  |  | Psy + Cog |  | 0.93 | 0.000 |
|  |  | Phy + Psy + Cog |  | 0.99 | 0.410 |
| Number of inpatient admissions |  | Phy |  | 0.98 | 0.136 |
|  |  | Psy |  | 0.93 | 0.007 |
|  |  | Cog |  | 1.01 | 0.852 |
|  |  | Phy + Psy |  | 0.99 | 0.588 |
|  |  | Phy + Cog |  | 1.05 | 0.288 |
|  |  | Psy + Cog |  | 0.91 | 0.107 |
|  |  | Phy + Psy + Cog |  | 1.00 | 0.981 |
| *Note:* Cog: cognitive disorder; Psy: psychological disorder; Phy: physical condition; Psy + Cog: psychological-cognitive multimorbidity; Phy + Cog: physical-cognitive multimorbidity; Phy + Psy: physical- psychological multimorbidity; Phy + Psy + Cog: physical-psychological-cognitive multimorbidity. | | | | | |

Table I. The association between multimorbidity and outpatient by country.

| Multimorbidity | United States | | | | China | | | | Japan | | | | South Korea | | | |
| --- | --- | --- | --- | --- | --- | --- | --- | --- | --- | --- | --- | --- | --- | --- | --- | --- |
|  | Odds ratio (95% CI) | P | Sigma | Rho | Odds ratio (95% CI) | P | Sigma | Rho | Odds ratio (95% CI) | P | Sigma | Rho | Odds ratio (95% CI) | P | Sigma | Rho |
| None (Ref) |  |  | 1.64 | 0.45 |  |  | 0.93 | 0.21 |  |  | 1.03 | 0.24 |  |  | 1.08 | 0.26 |
| Phy | 5.91 (5.17 to 6.75) | <0.001 |  |  | 1.90 (1.53 to 2.36) | <0.001 |  |  | 6.97 (4.49 to 10.82) | <0.001 |  |  | 3.55 (3.09 to 4.08) | <0.001 |  |  |
| Psy | 0.92 (0.68 to 1.23) | 0.560 |  |  | 1.99 (1.67 to 2.36) | <0.001 |  |  | 1.60 (1.16 to 2.21) | 0.004 |  |  | 0.98 (0.88 to 1.08) | 0.654 |  |  |
| Cog | 0.46 (0.10 to 2.12) | 0.320 |  |  | 0.93 (0.73 to 1.18) | 0.541 |  |  | 0.51 (0.25 to 1.04) | 0.064 |  |  | 0.95 (0.76 to 1.18) | 0.641 |  |  |
| Phy + Psy | 7.49 (6.19 to 9.07) | <0.001 |  |  | 4.05 (3.21 to 5.10) | <0.001 |  |  | 8.27 (4.64 to 14.74) | <0.001 |  |  | 2.37 (2.12 to 2.65) | <0.001 |  |  |
| Phy + Cog | 2.24 (1.49 to 3.37) | <0.001 |  |  | 1.94 (1.31 to 2.89) | 0.001 |  |  | 13.89 (3.92 to 49.23) | <0.001 |  |  | 3.38 (2.68 to 4.27) | <0.001 |  |  |
| Psy + Cog | 0.18 (0.02 to 1.57) | 0.122 |  |  | 2.17 (1.71 to 2.75) | <0.001 |  |  | 2.82 (0.41 to 19.22) | 0.291 |  |  | 1.08 (0.93 to 1.25) | 0.329 |  |  |
| Phy + Psy + Cog | 3.25 (1.74 to 6.09) | <0.001 |  |  | 2.75 (1.93 to 3.92) | <0.001 |  |  | 26.37 (2.70 to 257.34) | 0.005 |  |  | 2.76 (2.41 to 3.15) | <0.001 |  |  |

Table I. The association between multimorbidity and outpatient by country (continued).

| Multimorbidity | Mexico | | | | Austria | | | | Germany | | | | Sweden | | | |
| --- | --- | --- | --- | --- | --- | --- | --- | --- | --- | --- | --- | --- | --- | --- | --- | --- |
|  | Odds ratio (95% CI) | P | Sigma | Rho | Odds ratio (95% CI) | P | Sigma | Rho | Odds ratio (95% CI) | P | Sigma | Rho | Odds ratio (95% CI) | P | Sigma | Rho |
| None (Ref) |  |  | 1.02 | 0.24 |  |  | 1.41 | 0.38 |  |  | 1.88 | 0.52 |  |  | 1.28 | 0.33 |
| Phy | 3.87 (3.13 to 4.77) | <0.001 |  |  | 3.03 (2.47 to 3.71) | <0.001 |  |  | 7.42 (5.36 to 10.28) | <0.001 |  |  | 5.16 (4.28 to 6.21) | <0.001 |  |  |
| Psy | 1.36 (1.03 to 1.81) | 0.033 |  |  | 1.99 (1.33 to 2.99) | 0.001 |  |  | 2.53 (1.48 to 4.31) | 0.001 |  |  | 2.21 (1.59 to 3.09) | <0.001 |  |  |
| Cog | 0.64 (0.47 to 0.86) | 0.003 |  |  | 0.47 (0.16 to 1.44) | 0.188 |  |  | 0.34 (0.07 to 1.69) | 0.186 |  |  | 0.39 (0.13 to 1.16) | 0.091 |  |  |
| Phy + Psy | 3.77 (2.97 to 4.78) | <0.001 |  |  | 5.44 (3.92 to 7.55) | <0.001 |  |  | 11.22 (7.22 to 17.44) | <0.001 |  |  | 7.31 (5.51 to 9.69) | <0.001 |  |  |
| Phy + Cog | 2.74 (2.04 to 3.68) | <0.001 |  |  | 2.34 (0.81 to 6.75) | 0.115 |  |  | 2.79 (0.88 to 8.82) | 0.081 |  |  | 5.64 (1.71 to 18.63) | 0.005 |  |  |
| Psy + Cog | 0.87 (0.53 to 1.43) | 0.578 |  |  | 0.84 (0.17 to 4.12) | 0.826 |  |  | 0.14 (0.02 to 0.99) | 0.049 |  |  |  |  |  |  |
| Phy + Psy + Cog | 3.57 (2.64 to 4.81) | <0.001 |  |  | 1.82 (0.72 to 4.60) | 0.206 |  |  | 8.24 (1.45 to 46.90) | 0.017 |  |  | 5.62 (1.48 to 21.39) | 0.011 |  |  |

Table I. The association between multimorbidity and outpatient by country (continued).

| Multimorbidity | Netherlands | | | | Spain | | | | Italy | | | | France | | | |
| --- | --- | --- | --- | --- | --- | --- | --- | --- | --- | --- | --- | --- | --- | --- | --- | --- |
|  | Odds ratio (95% CI) | P | Sigma | Rho | Odds ratio (95% CI) | P | Sigma | Rho | Odds ratio (95% CI) | P | Sigma | Rho | Odds ratio (95% CI) | P | Sigma | Rho |
| None (Ref) |  |  | 1.33 | 0.35 |  |  | 1.25 | 0.32 |  |  | 1.24 | 0.32 |  |  | 1.83 | 0.50 |
| Phy | 5.63 (4.58 to 6.93) | <0.001 |  |  | 4.69 (3.81 to 5.78) | <0.001 |  |  | 3.70 (3.05 to 4.49) | <0.001 |  |  | 9.27 (6.90 to 12.46) | <0.001 |  |  |
| Psy | 2.25 (1.58 to 3.19) | <0.001 |  |  | 1.89 (1.32 to 2.70) | <0.001 |  |  | 1.70 (1.28 to 2.26) | <0.001 |  |  | 1.77 (1.24 to 2.53) | 0.002 |  |  |
| Cog | 2.47 (0.66 to 9.19) | 0.178 |  |  | 0.59 (0.36 to 0.97) | 0.039 |  |  | 0.73 (0.38 to 1.38) | 0.331 |  |  | 0.77 (0.27 to 2.14) | 0.611 |  |  |
| Phy + Psy | 11.37 (7.91 to 16.34) | <0.001 |  |  | 9.83 (7.12 to 13.57) | <0.001 |  |  | 6.11 (4.75 to 7.86) | <0.001 |  |  | 13.66 (9.21 to 20.26) | <0.001 |  |  |
| Phy + Cog | 11.56 (3.09 to 43.23) | <0.001 |  |  | 4.09 (2.39 to 6.99) | <0.001 |  |  | 3.07 (1.84 to 5.12) | <0.001 |  |  | 4.39 (1.63 to 11.78) | 0.003 |  |  |
| Psy + Cog | 1.84 (0.25 to 13.69) | 0.553 |  |  | 1.69 (0.70 to 4.08) | 0.239 |  |  | 1.98 (0.81 to 4.84) | 0.137 |  |  | 0.62 (0.19 to 2.05) | 0.438 |  |  |
| Phy + Psy + Cog | 21.43 (2.33 to 196.93) | 0.007 |  |  | 9.82 (5.20 to 18.54) | <0.001 |  |  | 4.30 (2.75 to 6.71) | <0.001 |  |  | 9.55 (2.32 to 39.43) | 0.002 |  |  |

Table I. The association between multimorbidity and outpatient by country (continued).

| Multimorbidity | Denmark | | | | Greece | | | | Switzerland | | | | Belgium | | | |
| --- | --- | --- | --- | --- | --- | --- | --- | --- | --- | --- | --- | --- | --- | --- | --- | --- |
|  | Odds ratio (95% CI) | P | Sigma | Rho | Odds ratio (95% CI) | P | Sigma | Rho | Odds ratio (95% CI) | P | Sigma | Rho | Odds ratio (95% CI) | P | Sigma | Rho |
| None (Ref) |  |  | 1.31 | 0.34 |  |  | 1.49 | 0.40 |  |  | 1.48 | 0.40 |  |  | 1.94 | 0.53 |
| Phy | 5.51 (4.48 to 6.76) | <0.001 |  |  | 6.59 (5.05 to 8.60) | <0.001 |  |  | 4.90 (3.97 to 6.06) | <0.001 |  |  | 6.55 (5.01 to 8.55) | <0.001 |  |  |
| Psy | 2.89 (1.87 to 4.47) | <0.001 |  |  | 1.27 (0.90 to 1.79) | 0.178 |  |  | 2.16 (1.48 to 3.14) | <0.001 |  |  | 2.08 (1.43 to 3.01) | <0.001 |  |  |
| Cog | 0.83 (0.06 to 10.88) | 0.890 |  |  | 1.08 (0.55 to 2.11) | 0.830 |  |  | 0.59 (0.18 to 1.90) | 0.376 |  |  | 1.91 (0.51 to 7.15) | 0.338 |  |  |
| Phy + Psy | 11.16 (7.68 to 16.22) | <0.001 |  |  | 5.65 (4.06 to 7.87) | <0.001 |  |  | 10.51 (7.20 to 15.34) | <0.001 |  |  | 10.44 (7.23 to 15.09) | <0.001 |  |  |
| Phy + Cog | 4.73 (1.20 to 18.68) | 0.027 |  |  | 3.53 (1.93 to 6.47) | <0.001 |  |  | 17.90 (2.28 to 140.65) | 0.006 |  |  | 2.84 (0.96 to 8.41) | 0.059 |  |  |
| Psy + Cog |  |  |  |  | 4.69 (1.12 to 19.61) | 0.034 |  |  |  |  |  |  | 1.38 (0.25 to 7.71) | 0.712 |  |  |
| Phy + Psy + Cog | 3.03 (0.52 to 17.72) | 0.219 |  |  | 8.11 (3.77 to 17.44) | <0.001 |  |  | 17.61 (2.16 to 143.74) | 0.007 |  |  | 6.55 (1.56 to 27.54) | 0.010 |  |  |

Table I. The association between multimorbidity and outpatient by country (continued).

| Multimorbidity | Israel | | | | Czech Republic | | | | Poland | | | | Luxembourg | | | |
| --- | --- | --- | --- | --- | --- | --- | --- | --- | --- | --- | --- | --- | --- | --- | --- | --- |
|  | Odds ratio (95% CI) | P | Sigma | Rho | Odds ratio (95% CI) | P | Sigma | Rho | Odds ratio (95% CI) | P | Sigma | Rho | Odds ratio (95% CI) | P | Sigma | Rho |
| None (Ref) |  |  | 1.43 | 0.38 |  |  | 1.48 | 0.40 |  |  | 1.59 | 0.43 |  |  | 1.44 | 0.39 |
| Phy | 4.23 (2.72 to 6.57) | <0.001 |  |  | 7.49 (5.86 to 9.57) | <0.001 |  |  | 11.83 (7.75 to 18.07) | <0.001 |  |  | 5.29 (2.78 to 10.09) | <0.001 |  |  |
| Psy | 6.58 (2.13 to 20.25) | 0.001 |  |  | 1.18 (0.80 to 1.73) | 0.407 |  |  | 1.41 (0.85 to 2.37) | 0.185 |  |  | 1.99 (0.78 to 5.10) | 0.152 |  |  |
| Cog | 0.28 (0.05 to 1.57) | 0.148 |  |  | 1.12 (0.28 to 4.43) | 0.870 |  |  | 0.49 (0.12 to 1.96) | 0.317 |  |  | 0.65 (0.08 to 5.33) | 0.688 |  |  |
| Phy + Psy | 6.19 (3.38 to 11.36) | <0.001 |  |  | 6.86 (4.99 to 9.42) | <0.001 |  |  | 14.37 (9.08 to 22.73) | <0.001 |  |  | 14.78 (5.49 to 39.78) | <0.001 |  |  |
| Phy + Cog | 1.64 (0.50 to 5.44) | 0.417 |  |  | 5.39 (1.70 to 17.07) | 0.004 |  |  | 11.41 (4.12 to 31.55) | <0.001 |  |  | 2.29 (0.42 to 12.48) | 0.338 |  |  |
| Psy + Cog | 0.34 (0.03 to 4.41) | 0.409 |  |  | 0.38 (0.08 to 1.77) | 0.216 |  |  | 5.25 (0.90 to 30.63) | 0.065 |  |  |  |  |  |  |
| Phy + Psy + Cog | 4.57 (1.16 to 17.92) | 0.029 |  |  | 10.42 (2.94 to 36.91) | <0.001 |  |  | 7.61 (3.57 to 16.22) | <0.001 |  |  | 5.12 (0.52 to 50.34) | 0.162 |  |  |

Table I. The association between multimorbidity and outpatient by country (continued).

| Multimorbidity | Hungary | | | | Portugal | | | | Slovenia | | | | Estonia | | | |
| --- | --- | --- | --- | --- | --- | --- | --- | --- | --- | --- | --- | --- | --- | --- | --- | --- |
|  | Odds ratio (95% CI) | P | Sigma | Rho | Odds ratio (95% CI) | P | Sigma | Rho | Odds ratio (95% CI) | P | Sigma | Rho | Odds ratio (95% CI) | P | Sigma | Rho |
| None (Ref) |  |  | 1.78 | 0.49 |  |  | 0.02 | 0.00 |  |  | 1.40 | 0.37 |  |  | 1.50 | 0.41 |
| Phy | 11.23 (6.31 to 19.98) | <0.001 |  |  | 2.60 (1.76 to 3.84) | <0.001 |  |  | 6.41 (5.16 to 7.98) | <0.001 |  |  | 6.10 (5.10 to 7.30) | <0.001 |  |  |
| Psy | 2.49 (1.25 to 4.98) | 0.010 |  |  | 1.36 (0.79 to 2.34) | 0.273 |  |  | 1.59 (1.16 to 2.18) | 0.004 |  |  | 1.41 (1.11 to 1.79) | 0.005 |  |  |
| Cog | 0.43 (0.08 to 2.30) | 0.324 |  |  | 2.66 (0.93 to 7.64) | 0.069 |  |  | 0.30 (0.11 to 0.84) | 0.022 |  |  | 0.24 (0.10 to 0.59) | 0.002 |  |  |
| Phy + Psy | 17.82 (8.81 to 36.03) | <0.001 |  |  | 5.56 (3.24 to 9.55) | <0.001 |  |  | 8.32 (6.12 to 11.31) | <0.001 |  |  | 9.75 (7.93 to 11.99) | <0.001 |  |  |
| Phy + Cog | 16.08 (3.30 to 78.32) | 0.001 |  |  | 1.57 (0.68 to 3.62) | 0.286 |  |  | 6.19 (2.22 to 17.27) | <0.001 |  |  | 2.70 (1.45 to 5.02) | 0.002 |  |  |
| Psy + Cog | 15.88 (0.73 to 346.11) | 0.079 |  |  | 4.86 (0.60 to 39.63) | 0.140 |  |  | 1.84 (0.45 to 7.51) | 0.396 |  |  | 1.43 (0.54 to 3.76) | 0.472 |  |  |
| Phy + Psy + Cog | 19.36 (5.75 to 65.19) | <0.001 |  |  | 3.61 (1.47 to 8.87) | 0.005 |  |  | 2.31 (1.10 to 4.82) | 0.026 |  |  | 7.67 (4.39 to 13.41) | <0.001 |  |  |

Table I. The association between multimorbidity and outpatient by country (continued).

| Multimorbidity | Croatia | | | | Lithuania | | | | Bulgaria | | | | Cyprus | | | |
| --- | --- | --- | --- | --- | --- | --- | --- | --- | --- | --- | --- | --- | --- | --- | --- | --- |
|  | Odds ratio (95% CI) | P | Sigma | Rho | Odds ratio (95% CI) | P | Sigma | Rho | Odds ratio (95% CI) | P | Sigma | Rho | Odds ratio (95% CI) | P | Sigma | Rho |
| None (Ref) |  |  | 1.07 | 0.26 |  |  | 0.23 | 0.02 |  |  | 0.01 | 0.00 |  |  | 2.67 | 0.68 |
| Phy | 6.30 (4.49 to 8.85) | <0.001 |  |  | 4.54 (0.03 to 660.43) | 0.552 |  |  | 6.42 (3.69 to 11.17) | <0.001 |  |  | 1.30 (0.34 to 4.96) | 0.702 |  |  |
| Psy | 1.81 (1.14 to 2.86) | 0.011 |  |  | 1.95 (0.09 to 40.91) | 0.666 |  |  | 4.08 (1.05 to 15.84) | 0.042 |  |  | 1.24 (0.03 to 53.60) | 0.911 |  |  |
| Cog | 0.88 (0.28 to 2.79) | 0.834 |  |  | 0.10 (0.00 to 75227.25) | 0.735 |  |  | 14.88 (5.35 to 41.36) | <0.001 |  |  | 0.03 (0.00 to 19.82) | 0.280 |  |  |
| Phy + Psy | 9.38 (5.95 to 14.78) | <0.001 |  |  | 6.44 (0.03 to 1631.44) | 0.510 |  |  |  |  |  |  | 7.34 (0.21 to 256.97) | 0.272 |  |  |
| Phy + Cog | 2.28 (0.77 to 6.76) | 0.136 |  |  | 1.29 (0.15 to 11.46) | 0.819 |  |  | 2.35 (0.35 to 15.53) | 0.376 |  |  | 3.32 (0.14 to 76.93) | 0.454 |  |  |
| Psy + Cog | 1.19 (0.17 to 8.24) | 0.862 |  |  |  |  |  |  | 8.04 (1.57 to 41.25) | 0.012 |  |  |  |  |  |  |
| Phy + Psy + Cog | 6.55 (2.27 to 18.95) | 0.001 |  |  | 11.07 (0.01 to 13684.47) | 0.508 |  |  | 1.01 (0.98 to 1.05) | 0.460 |  |  | 0.16 (0.00 to 6.18) | 0.324 |  |  |

Table I. The association between multimorbidity and outpatient by country (continued).

| Multimorbidity | Finland | | | | Latvia | | | | Romania | | | | Slovakia | | | |
| --- | --- | --- | --- | --- | --- | --- | --- | --- | --- | --- | --- | --- | --- | --- | --- | --- |
|  | Odds ratio (95% CI) | P | Sigma | Rho | Odds ratio (95% CI) | P | Sigma | Rho | Odds ratio (95% CI) | P | Sigma | Rho | Odds ratio (95% CI) | P | Sigma | Rho |
| None (Ref) |  |  | 2.28 | 0.61 |  |  | 2.52 | 0.66 |  |  | 0.01 | 0.00 |  |  | 0.01 | 0.00 |
| Phy | 3.07 (1.54 to 6.10) | 0.001 |  |  | 10.40 (3.93 to 27.57) | <0.001 |  |  | 6.35 (4.30 to 9.38) | <0.001 |  |  | 3.85 (1.80 to 8.24) | 0.001 |  |  |
| Psy | 1.24 (0.37 to 4.12) | 0.728 |  |  | 0.42 (0.06 to 2.92) | 0.380 |  |  | 1.30 (0.72 to 2.33) | 0.381 |  |  | 2.25 (0.85 to 5.99) | 0.103 |  |  |
| Cog |  |  |  |  | 0.12 (0.00 to 4.60) | 0.254 |  |  | 2.85 (1.05 to 7.77) | 0.040 |  |  |  |  |  |  |
| Phy + Psy | 9.35 (3.69 to 23.73) | <0.001 |  |  | 14.74 (4.11 to 52.85) | <0.001 |  |  | 11.23 (6.77 to 18.63) | <0.001 |  |  | 5.57 (1.71 to 18.10) | 0.004 |  |  |
| Phy + Cog | 3.15 (0.15 to 67.97) | 0.464 |  |  | 1.53 (0.11 to 21.19) | 0.752 |  |  | 5.12 (1.51 to 17.33) | 0.009 |  |  | 0.17 (0.01 to 2.99) | 0.228 |  |  |
| Psy + Cog |  |  |  |  |  |  |  |  | 0.41 (0.12 to 1.42) | 0.161 |  |  |  |  |  |  |
| Phy + Psy + Cog | 1.02 (0.97 to 1.06) | 0.475 |  |  | 40.44 (2.57 to 637.23) | 0.009 |  |  | 2.87 (1.45 to 5.68) | 0.002 |  |  | 3.65 (0.56 to 23.67) | 0.174 |  |  |
| *Note:* Cog: cognitive disorder; Psy: psychological disorder; Phy: physical condition; Psy + Cog: psychological-cognitive multimorbidity; Phy + Cog: physical-cognitive multimorbidity; Phy + Psy: physical- psychological multimorbidity; Phy + Psy + Cog: physical-psychological-cognitive multimorbidity. Sigma (σᵤ) denotes the standard deviation of the individual-level random intercept, reflecting unobserved heterogeneity between individuals. Rho (ρ) represents the intra-class correlation coefficient, indicating the proportion of total variance attributable to stable between-individual differences. | | | | | | | | | | | | | | | | |

Table J. The association between multimorbidity and number of outpatient visits by country.

| Multimorbidity | United States | | | China | | | Japan | | | South Korea | | |
| --- | --- | --- | --- | --- | --- | --- | --- | --- | --- | --- | --- | --- |
|  | Incident rate ratio (95% CI) | P | r | Incident rate ratio (95% CI) | P | r | Incident rate ratio (95% CI) | P | r | Incident rate ratio (95% CI) | P | r |
| None (Ref) |  |  | 4.09 (3.97 to 4.22) |  |  | 1.74e+06 (1.57e+06 to 1.94e+06) |  |  | 6.09 (4.28 to 8.67) |  |  | 3.49 (3.34 to 3.64) |
| Phy | 1.89 (1.82 to 1.95) | <0.001 | 4.09 (3.97 to 4.22) | 1.66 (1.41 to 1.95) | <0.001 | 1.74e+06 (1.57e+06 to 1.94e+06) | 3.38 (3.09 to 3.70) | <0.001 | 6.09 (4.28 to 8.67) | 2.21 (2.12 to 2.31) | <0.001 | 3.49 (3.34 to 3.64) |
| Psy | 1.13 (1.02 to 1.25) | 0.018 | 4.09 (3.97 to 4.22) | 1.79 (1.56 to 2.04) | <0.001 | 1.74e+06 (1.57e+06 to 1.94e+06) | 1.47 (1.22 to 1.76) | <0.001 | 6.09 (4.28 to 8.67) | 1.00 (0.96 to 1.04) | 0.931 | 3.49 (3.34 to 3.64) |
| Cog | 0.89 (0.55 to 1.44) | 0.641 | 4.09 (3.97 to 4.22) | 0.91 (0.74 to 1.11) | 0.354 | 1.74e+06 (1.57e+06 to 1.94e+06) | 0.92 (0.56 to 1.50) | 0.730 | 6.09 (4.28 to 8.67) | 1.06 (0.97 to 1.17) | 0.203 | 3.49 (3.34 to 3.64) |
| Phy + Psy | 2.20 (2.11 to 2.29) | <0.001 | 4.09 (3.97 to 4.22) | 3.15 (2.70 to 3.67) | <0.001 | 1.74e+06 (1.57e+06 to 1.94e+06) | 3.75 (3.28 to 4.29) | <0.001 | 6.09 (4.28 to 8.67) | 1.90 (1.82 to 1.98) | <0.001 | 3.49 (3.34 to 3.64) |
| Phy + Cog | 1.47 (1.31 to 1.65) | <0.001 | 4.09 (3.97 to 4.22) | 1.60 (1.17 to 2.19) | 0.003 | 1.74e+06 (1.57e+06 to 1.94e+06) | 4.39 (3.26 to 5.92) | <0.001 | 6.09 (4.28 to 8.67) | 2.04 (1.92 to 2.17) | <0.001 | 3.49 (3.34 to 3.64) |
| Psy + Cog | 0.41 (0.10 to 1.62) | 0.201 | 4.09 (3.97 to 4.22) | 1.92 (1.60 to 2.29) | <0.001 | 1.74e+06 (1.57e+06 to 1.94e+06) | 1.80 (0.58 to 5.54) | 0.307 | 6.09 (4.28 to 8.67) | 1.10 (1.04 to 1.17) | 0.001 | 3.49 (3.34 to 3.64) |
| Phy + Psy + Cog | 1.50 (1.27 to 1.77) | <0.001 | 4.09 (3.97 to 4.22) | 2.32 (1.79 to 3.00) | <0.001 | 1.74e+06 (1.57e+06 to 1.94e+06) | 4.20 (2.68 to 6.59) | <0.001 | 6.09 (4.28 to 8.67) | 1.97 (1.88 to 2.06) | <0.001 | 3.49 (3.34 to 3.64) |

Table J. The association between multimorbidity and number of outpatient visits by country (continued).

| Multimorbidity | Austria | | | Germany | | | Sweden | | | Netherlands | | |
| --- | --- | --- | --- | --- | --- | --- | --- | --- | --- | --- | --- | --- |
|  | Incident rate ratio (95% CI) | P | r | Incident rate ratio (95% CI) | P | r | Incident rate ratio (95% CI) | P | r | Incident rate ratio (95% CI) | P | r |
| None (Ref) |  |  | 3.49 (3.34 to 3.64) |  |  | 3.88 (3.64 to 4.15) |  |  | 3.97 (3.75 to 4.21) |  |  | 4.15 (3.87 to 4.46) |
| Phy | 2.21 (2.12 to 2.31) | <0.001 | 3.49 (3.34 to 3.64) | 1.57 (1.49 to 1.64) | <0.001 | 3.88 (3.64 to 4.15) | 1.71 (1.62 to 1.80) | <0.001 | 3.97 (3.75 to 4.21) | 1.85 (1.74 to 1.96) | <0.001 | 4.15 (3.87 to 4.46) |
| Psy | 1.00 (0.96 to 1.04) | 0.931 | 3.49 (3.34 to 3.64) | 1.42 (1.29 to 1.56) | <0.001 | 3.88 (3.64 to 4.15) | 1.35 (1.23 to 1.48) | <0.001 | 3.97 (3.75 to 4.21) | 1.53 (1.36 to 1.72) | <0.001 | 4.15 (3.87 to 4.46) |
| Cog | 1.06 (0.97 to 1.17) | 0.203 | 3.49 (3.34 to 3.64) | 0.69 (0.47 to 1.02) | 0.065 | 3.88 (3.64 to 4.15) | 0.62 (0.38 to 1.02) | 0.061 | 3.97 (3.75 to 4.21) | 0.63 (0.34 to 1.17) | 0.142 | 4.15 (3.87 to 4.46) |
| Phy + Psy | 1.90 (1.82 to 1.98) | <0.001 | 3.49 (3.34 to 3.64) | 2.02 (1.90 to 2.14) | <0.001 | 3.88 (3.64 to 4.15) | 2.18 (2.05 to 2.31) | <0.001 | 3.97 (3.75 to 4.21) | 2.50 (2.32 to 2.70) | <0.001 | 4.15 (3.87 to 4.46) |
| Phy + Cog | 2.04 (1.92 to 2.17) | <0.001 | 3.49 (3.34 to 3.64) | 1.67 (1.36 to 2.06) | <0.001 | 3.88 (3.64 to 4.15) | 1.95 (1.63 to 2.33) | <0.001 | 3.97 (3.75 to 4.21) | 1.69 (1.28 to 2.22) | <0.001 | 4.15 (3.87 to 4.46) |
| Psy + Cog | 1.10 (1.04 to 1.17) | 0.001 | 3.49 (3.34 to 3.64) | 1.10 (0.71 to 1.71) | 0.657 | 3.88 (3.64 to 4.15) | 0.63 (0.32 to 1.26) | 0.191 | 3.97 (3.75 to 4.21) | 1.42 (0.37 to 5.45) | 0.605 | 4.15 (3.87 to 4.46) |
| Phy + Psy + Cog | 1.97 (1.88 to 2.06) | <0.001 | 3.49 (3.34 to 3.64) | 1.67 (1.37 to 2.03) | <0.001 | 3.88 (3.64 to 4.15) | 2.45 (2.04 to 2.92) | <0.001 | 3.97 (3.75 to 4.21) | 2.23 (1.67 to 2.96) | <0.001 | 4.15 (3.87 to 4.46) |

Table J. The association between multimorbidity and number of outpatient visits by country (continued).

| Multimorbidity | Spain | | | Italy | | | France | | | Denmark | | |
| --- | --- | --- | --- | --- | --- | --- | --- | --- | --- | --- | --- | --- |
|  | Incident rate ratio (95% CI) | P | r | Incident rate ratio (95% CI) | P | r | Incident rate ratio (95% CI) | P | r | Incident rate ratio (95% CI) | P | r |
| None (Ref) |  |  | 3.61 (3.36 to 3.88) |  |  | 5.38 (5.03 to 5.75) |  |  | 4.42 (4.10 to 4.77) |  |  | 7.14 (6.74 to 7.57) |
| Phy | 1.97 (1.86 to 2.09) | <0.001 | 3.61 (3.36 to 3.88) | 1.85 (1.76 to 1.95) | <0.001 | 5.38 (5.03 to 5.75) | 1.77 (1.68 to 1.86) | <0.001 | 4.42 (4.10 to 4.77) | 1.83 (1.75 to 1.91) | <0.001 | 7.14 (6.74 to 7.57) |
| Psy | 1.49 (1.33 to 1.66) | <0.001 | 3.61 (3.36 to 3.88) | 1.43 (1.30 to 1.57) | <0.001 | 5.38 (5.03 to 5.75) | 1.30 (1.19 to 1.42) | <0.001 | 4.42 (4.10 to 4.77) | 1.38 (1.29 to 1.48) | <0.001 | 7.14 (6.74 to 7.57) |
| Cog | 1.38 (0.90 to 2.11) | 0.142 | 3.61 (3.36 to 3.88) | 0.91 (0.75 to 1.11) | 0.344 | 5.38 (5.03 to 5.75) | 0.84 (0.66 to 1.06) | 0.147 | 4.42 (4.10 to 4.77) | 1.18 (0.94 to 1.47) | 0.152 | 7.14 (6.74 to 7.57) |
| Phy + Psy | 2.77 (2.58 to 2.98) | <0.001 | 3.61 (3.36 to 3.88) | 2.40 (2.27 to 2.54) | <0.001 | 5.38 (5.03 to 5.75) | 2.23 (2.11 to 2.36) | <0.001 | 4.42 (4.10 to 4.77) | 2.21 (2.10 to 2.32) | <0.001 | 7.14 (6.74 to 7.57) |
| Phy + Cog | 2.34 (1.87 to 2.94) | <0.001 | 3.61 (3.36 to 3.88) | 1.76 (1.60 to 1.94) | <0.001 | 5.38 (5.03 to 5.75) | 1.74 (1.56 to 1.94) | <0.001 | 4.42 (4.10 to 4.77) | 1.84 (1.62 to 2.08) | <0.001 | 7.14 (6.74 to 7.57) |
| Psy + Cog | 1.92 (0.99 to 3.72) | 0.052 | 3.61 (3.36 to 3.88) | 1.60 (1.30 to 1.97) | <0.001 | 5.38 (5.03 to 5.75) | 1.53 (1.23 to 1.90) | <0.001 | 4.42 (4.10 to 4.77) | 1.58 (1.23 to 2.03) | <0.001 | 7.14 (6.74 to 7.57) |
| Phy + Psy + Cog | 2.82 (2.17 to 3.67) | <0.001 | 3.61 (3.36 to 3.88) | 2.33 (2.14 to 2.52) | <0.001 | 5.38 (5.03 to 5.75) | 2.24 (2.06 to 2.44) | <0.001 | 4.42 (4.10 to 4.77) | 2.34 (2.10 to 2.60) | <0.001 | 7.14 (6.74 to 7.57) |

Table J. The association between multimorbidity and number of outpatient visits by country (continued).

| Multimorbidity | Greece | | | Switzerland | | | Belgium | | | Israel | | |
| --- | --- | --- | --- | --- | --- | --- | --- | --- | --- | --- | --- | --- |
|  | Incident rate ratio (95% CI) | P | r | Incident rate ratio (95% CI) | P | r | Incident rate ratio (95% CI) | P | r | Incident rate ratio (95% CI) | P | r |
| None (Ref) |  |  | 4.00 (3.72 to 4.29) |  |  | 5.37 (4.92 to 5.85) |  |  | 3.57 (3.33 to 3.83) |  |  | 4.39 (4.17 to 4.63) |
| Phy | 1.95 (1.84 to 2.07) | <0.001 | 4.00 (3.72 to 4.29) | 2.21 (2.06 to 2.37) | <0.001 | 5.37 (4.92 to 5.85) | 1.81 (1.71 to 1.91) | <0.001 | 3.57 (3.33 to 3.83) | 1.77 (1.69 to 1.85) | <0.001 | 4.39 (4.17 to 4.63) |
| Psy | 1.67 (1.47 to 1.89) | <0.001 | 4.00 (3.72 to 4.29) | 1.17 (1.03 to 1.33) | 0.015 | 5.37 (4.92 to 5.85) | 1.43 (1.28 to 1.60) | <0.001 | 3.57 (3.33 to 3.83) | 1.45 (1.35 to 1.56) | <0.001 | 4.39 (4.17 to 4.63) |
| Cog | 0.70 (0.30 to 1.62) | 0.404 | 4.00 (3.72 to 4.29) | 1.16 (0.89 to 1.51) | 0.266 | 5.37 (4.92 to 5.85) | 0.83 (0.48 to 1.45) | 0.511 | 3.57 (3.33 to 3.83) | 1.12 (0.85 to 1.47) | 0.417 | 4.39 (4.17 to 4.63) |
| Phy + Psy | 2.71 (2.52 to 2.92) | <0.001 | 4.00 (3.72 to 4.29) | 2.32 (2.14 to 2.52) | <0.001 | 5.37 (4.92 to 5.85) | 2.41 (2.24 to 2.58) | <0.001 | 3.57 (3.33 to 3.83) | 2.16 (2.06 to 2.27) | <0.001 | 4.39 (4.17 to 4.63) |
| Phy + Cog | 2.35 (1.79 to 3.08) | <0.001 | 4.00 (3.72 to 4.29) | 1.86 (1.60 to 2.15) | <0.001 | 5.37 (4.92 to 5.85) | 2.72 (2.00 to 3.69) | <0.001 | 3.57 (3.33 to 3.83) | 1.92 (1.66 to 2.21) | <0.001 | 4.39 (4.17 to 4.63) |
| Psy + Cog | 2.33 (1.07 to 5.05) | 0.032 | 4.00 (3.72 to 4.29) | 1.50 (1.08 to 2.08) | 0.017 | 5.37 (4.92 to 5.85) | 4.33 (2.07 to 9.07) | <0.001 | 3.57 (3.33 to 3.83) | 1.61 (1.12 to 2.33) | 0.011 | 4.39 (4.17 to 4.63) |
| Phy + Psy + Cog | 3.19 (2.25 to 4.51) | <0.001 | 4.00 (3.72 to 4.29) | 2.28 (2.00 to 2.61) | <0.001 | 5.37 (4.92 to 5.85) | 3.13 (2.37 to 4.13) | <0.001 | 3.57 (3.33 to 3.83) | 2.44 (2.13 to 2.80) | <0.001 | 4.39 (4.17 to 4.63) |

Table J. The association between multimorbidity and number of outpatient visits by country (continued).

| Multimorbidity | Czech Republic | | | Poland | | | Luxembourg | | | Hungary | | |
| --- | --- | --- | --- | --- | --- | --- | --- | --- | --- | --- | --- | --- |
|  | Incident rate ratio (95% CI) | P | r | Incident rate ratio (95% CI) | P | r | Incident rate ratio (95% CI) | P | r | Incident rate ratio (95% CI) | P | r |
| None (Ref) |  |  | 3.87 (3.46 to 4.34) |  |  | 5.46 (5.16 to 5.78) |  |  | 8.06 (6.59 to 9.84) |  |  | 8.06 (6.59 to 9.84) |
| Phy | 1.82 (1.65 to 2.00) | <0.001 | 3.87 (3.46 to 4.34) | 1.97 (1.88 to 2.06) | <0.001 | 5.46 (5.16 to 5.78) | 2.85 (2.58 to 3.14) | <0.001 | 8.06 (6.59 to 9.84) | 2.85 (2.58 to 3.14) | <0.001 | 8.06 (6.59 to 9.84) |
| Psy | 1.68 (1.40 to 2.03) | <0.001 | 3.87 (3.46 to 4.34) | 1.39 (1.27 to 1.53) | <0.001 | 5.46 (5.16 to 5.78) | 1.32 (1.11 to 1.58) | 0.002 | 8.06 (6.59 to 9.84) | 1.32 (1.11 to 1.58) | 0.002 | 8.06 (6.59 to 9.84) |
| Cog | 0.59 (0.25 to 1.38) | 0.220 | 3.87 (3.46 to 4.34) | 1.17 (0.79 to 1.75) | 0.435 | 5.46 (5.16 to 5.78) | 0.52 (0.26 to 1.03) | 0.062 | 8.06 (6.59 to 9.84) | 0.52 (0.26 to 1.03) | 0.062 | 8.06 (6.59 to 9.84) |
| Phy + Psy | 2.44 (2.17 to 2.74) | <0.001 | 3.87 (3.46 to 4.34) | 2.36 (2.24 to 2.49) | <0.001 | 5.46 (5.16 to 5.78) | 3.39 (3.06 to 3.76) | <0.001 | 8.06 (6.59 to 9.84) | 3.39 (3.06 to 3.76) | <0.001 | 8.06 (6.59 to 9.84) |
| Phy + Cog | 1.37 (1.01 to 1.84) | 0.040 | 3.87 (3.46 to 4.34) | 2.01 (1.74 to 2.32) | <0.001 | 5.46 (5.16 to 5.78) | 2.69 (2.23 to 3.24) | <0.001 | 8.06 (6.59 to 9.84) | 2.69 (2.23 to 3.24) | <0.001 | 8.06 (6.59 to 9.84) |
| Psy + Cog | 1.25 (0.47 to 3.30) | 0.657 | 3.87 (3.46 to 4.34) | 0.85 (0.50 to 1.43) | 0.535 | 5.46 (5.16 to 5.78) | 1.77 (1.09 to 2.85) | 0.020 | 8.06 (6.59 to 9.84) | 1.77 (1.09 to 2.85) | 0.020 | 8.06 (6.59 to 9.84) |
| Phy + Psy + Cog | 2.13 (1.67 to 2.71) | <0.001 | 3.87 (3.46 to 4.34) | 2.32 (2.03 to 2.65) | <0.001 | 5.46 (5.16 to 5.78) | 2.69 (2.29 to 3.17) | <0.001 | 8.06 (6.59 to 9.84) | 2.69 (2.29 to 3.17) | <0.001 | 8.06 (6.59 to 9.84) |

Table J. The association between multimorbidity and number of outpatient visits by country (continued).

| Multimorbidity | Portugal | | | Slovenia | | | Estonia | | | Croatia | | |
| --- | --- | --- | --- | --- | --- | --- | --- | --- | --- | --- | --- | --- |
|  | Incident rate ratio (95% CI) | P | r | Incident rate ratio (95% CI) | P | r | Incident rate ratio (95% CI) | P | r | Incident rate ratio (95% CI) | P | r |
| None (Ref) |  |  | 4.20 (3.79 to 4.65) |  |  | 7.90 (6.33 to 9.86) |  |  | 4.93 (4.40 to 5.53) |  |  | 4.22 (3.93 to 4.52) |
| Phy | 1.64 (1.50 to 1.80) | <0.001 | 4.20 (3.79 to 4.65) | 2.50 (2.26 to 2.77) | <0.001 | 7.90 (6.33 to 9.86) | 1.59 (1.40 to 1.81) | <0.001 | 4.93 (4.40 to 5.53) | 1.96 (1.85 to 2.07) | <0.001 | 4.22 (3.93 to 4.52) |
| Psy | 1.37 (1.16 to 1.61) | <0.001 | 4.20 (3.79 to 4.65) | 1.52 (1.25 to 1.85) | <0.001 | 7.90 (6.33 to 9.86) | 1.50 (1.23 to 1.82) | <0.001 | 4.93 (4.40 to 5.53) | 1.41 (1.27 to 1.56) | <0.001 | 4.22 (3.93 to 4.52) |
| Cog | 0.88 (0.52 to 1.47) | 0.615 | 4.20 (3.79 to 4.65) | 0.49 (0.22 to 1.08) | 0.077 | 7.90 (6.33 to 9.86) | 1.24 (0.89 to 1.74) | 0.201 | 4.93 (4.40 to 5.53) | 0.56 (0.34 to 0.90) | 0.017 | 4.22 (3.93 to 4.52) |
| Phy + Psy | 2.05 (1.85 to 2.28) | <0.001 | 4.20 (3.79 to 4.65) | 3.22 (2.89 to 3.58) | <0.001 | 7.90 (6.33 to 9.86) | 2.29 (2.00 to 2.63) | <0.001 | 4.93 (4.40 to 5.53) | 2.28 (2.13 to 2.44) | <0.001 | 4.22 (3.93 to 4.52) |
| Phy + Cog | 1.62 (1.20 to 2.19) | 0.002 | 4.20 (3.79 to 4.65) | 2.75 (2.09 to 3.61) | <0.001 | 7.90 (6.33 to 9.86) | 1.67 (1.26 to 2.20) | <0.001 | 4.93 (4.40 to 5.53) | 2.10 (1.71 to 2.57) | <0.001 | 4.22 (3.93 to 4.52) |
| Psy + Cog | 1.78 (0.95 to 3.31) | 0.071 | 4.20 (3.79 to 4.65) | 2.68 (1.56 to 4.60) | <0.001 | 7.90 (6.33 to 9.86) | 1.85 (1.22 to 2.81) | 0.004 | 4.93 (4.40 to 5.53) | 1.35 (0.82 to 2.21) | 0.240 | 4.22 (3.93 to 4.52) |
| Phy + Psy + Cog | 1.67 (1.23 to 2.26) | 0.001 | 4.20 (3.79 to 4.65) | 3.33 (2.80 to 3.95) | <0.001 | 7.90 (6.33 to 9.86) | 2.17 (1.77 to 2.66) | <0.001 | 4.93 (4.40 to 5.53) | 1.90 (1.57 to 2.31) | <0.001 | 4.22 (3.93 to 4.52) |

Table J. The association between multimorbidity and number of outpatient visits by country (continued).

| Multimorbidity | Lithuania | | | Bulgaria | | | Cyprus | | | Finland | | |
| --- | --- | --- | --- | --- | --- | --- | --- | --- | --- | --- | --- | --- |
|  | Incident rate ratio (95% CI) | P | r | Incident rate ratio (95% CI) | P | r | Incident rate ratio (95% CI) | P | r | Incident rate ratio (95% CI) | P | r |
| None (Ref) |  |  | 4.06 (3.83 to 4.30) |  |  | 4.60 (3.84 to 5.51) |  |  | 7.20 (6.09 to 8.51) |  |  | 5.14e+06 (0.00 to .) |
| Phy | 2.18 (2.07 to 2.30) | <0.001 | 4.06 (3.83 to 4.30) | 2.45 (2.22 to 2.69) | <0.001 | 4.60 (3.84 to 5.51) | 2.39 (2.05 to 2.80) | <0.001 | 7.20 (6.09 to 8.51) | 2.49 (2.01 to 3.08) | <0.001 | 5.14e+06 (0.00 to .) |
| Psy | 1.30 (1.19 to 1.42) | <0.001 | 4.06 (3.83 to 4.30) | 1.56 (1.31 to 1.86) | <0.001 | 4.60 (3.84 to 5.51) | 1.55 (1.17 to 2.07) | 0.002 | 7.20 (6.09 to 8.51) | 1.66 (1.02 to 2.71) | 0.042 | 5.14e+06 (0.00 to .) |
| Cog | 0.50 (0.28 to 0.89) | 0.019 | 4.06 (3.83 to 4.30) | 1.00 (0.60 to 1.66) | 1.000 | 4.60 (3.84 to 5.51) | 0.18 (0.03 to 1.26) | 0.084 | 7.20 (6.09 to 8.51) | 3.29 (2.59 to 4.18) | <0.001 | 5.14e+06 (0.00 to .) |
| Phy + Psy | 2.72 (2.57 to 2.88) | <0.001 | 4.06 (3.83 to 4.30) | 3.16 (2.84 to 3.52) | <0.001 | 4.60 (3.84 to 5.51) | 2.94 (2.49 to 3.47) | <0.001 | 7.20 (6.09 to 8.51) | 2.88 (1.85 to 4.47) | <0.001 | 5.14e+06 (0.00 to .) |
| Phy + Cog | 1.92 (1.59 to 2.31) | <0.001 | 4.06 (3.83 to 4.30) | 2.21 (1.57 to 3.13) | <0.001 | 4.60 (3.84 to 5.51) | 1.86 (1.17 to 2.98) | 0.009 | 7.20 (6.09 to 8.51) | 1.37 (0.51 to 3.67) | 0.537 | 5.14e+06 (0.00 to .) |
| Psy + Cog | 1.20 (0.73 to 1.95) | 0.474 | 4.06 (3.83 to 4.30) | 1.56 (0.70 to 3.49) | 0.280 | 4.60 (3.84 to 5.51) | 1.73 (0.71 to 4.19) | 0.227 | 7.20 (6.09 to 8.51) | 2.26 (1.52 to 3.36) | <0.001 | 5.14e+06 (0.00 to .) |
| Phy + Psy + Cog | 2.58 (2.29 to 2.92) | <0.001 | 4.06 (3.83 to 4.30) | 2.29 (1.79 to 2.91) | <0.001 | 4.60 (3.84 to 5.51) | 3.02 (2.13 to 4.27) | <0.001 | 7.20 (6.09 to 8.51) | 1.01 (1.00 to 1.01) | 0.079 | 5.14e+06 (0.00 to .) |

Table J. The association between multimorbidity and number of outpatient visits by country (continued).

| Multimorbidity | Latvia | | | Romania | | | Slovakia | | |
| --- | --- | --- | --- | --- | --- | --- | --- | --- | --- |
|  | Incident rate ratio (95% CI) | P | r | Incident rate ratio (95% CI) | P | r | Incident rate ratio (95% CI) | P | r |
| None (Ref) |  |  | 5.55 (4.29 to 7.18) |  |  | 5.14 (4.18 to 6.33) |  |  | 10.30 (6.36 to 16.67) |
| Phy | 1.56 (1.20 to 2.03) | 0.001 | 5.55 (4.29 to 7.18) | 1.58 (1.33 to 1.89) | <0.001 | 5.14 (4.18 to 6.33) | 2.02 (1.66 to 2.47) | <0.001 | 10.30 (6.36 to 16.67) |
| Psy | 1.13 (0.53 to 2.41) | 0.752 | 5.55 (4.29 to 7.18) | 1.22 (0.88 to 1.67) | 0.231 | 5.14 (4.18 to 6.33) | 1.19 (0.75 to 1.88) | 0.462 | 10.30 (6.36 to 16.67) |
| Cog | 0.58 (0.22 to 1.57) | 0.285 | 5.55 (4.29 to 7.18) | 0.36 (0.04 to 3.62) | 0.388 | 5.14 (4.18 to 6.33) | 1.32 (0.57 to 3.08) | 0.521 | 10.30 (6.36 to 16.67) |
| Phy + Psy | 2.20 (1.60 to 3.03) | <0.001 | 5.55 (4.29 to 7.18) | 2.31 (1.89 to 2.83) | <0.001 | 5.14 (4.18 to 6.33) | 2.42 (1.94 to 3.01) | <0.001 | 10.30 (6.36 to 16.67) |
| Phy + Cog | 1.76 (1.16 to 2.68) | 0.008 | 5.55 (4.29 to 7.18) | 1.28 (0.51 to 3.24) | 0.597 | 5.14 (4.18 to 6.33) | 1.25 (0.69 to 2.28) | 0.461 | 10.30 (6.36 to 16.67) |
| Psy + Cog | 2.16 (0.41 to 11.51) | 0.366 | 5.55 (4.29 to 7.18) | 2.69 (0.97 to 7.46) | 0.057 | 5.14 (4.18 to 6.33) | 0.57 (0.07 to 4.52) | 0.596 | 10.30 (6.36 to 16.67) |
| Phy + Psy + Cog | 1.04 (0.57 to 1.89) | 0.899 | 5.55 (4.29 to 7.18) | 1.00 (0.99 to 1.00) | 0.290 | 5.14 (4.18 to 6.33) | 2.31 (1.49 to 3.57) | <0.001 | 10.30 (6.36 to 16.67) |
| *Note:* Cog: cognitive disorder; Psy: psychological disorder; Phy: physical condition; Psy + Cog: psychological-cognitive multimorbidity; Phy + Cog: physical-cognitive multimorbidity; Phy + Psy: physical- psychological multimorbidity; Phy + Psy + Cog: physical-psychological-cognitive multimorbidity. These are estimated by random effect negative binomial regression model. The dispersion parameter (r) from the negative binomial model was significantly greater than zero across countries, indicating overdispersion and justifying the use of negative binomial rather than Poisson models. | | | | | | | | | |

Table K. The association between multimorbidity and inpatient by country.

| Multimorbidity | United States | | | | China | | | | Japan | | | | South Korea | | | |
| --- | --- | --- | --- | --- | --- | --- | --- | --- | --- | --- | --- | --- | --- | --- | --- | --- |
|  | Odds ratio (95% CI) | P | Sigma | Rho | Odds ratio (95% CI) | P | Sigma | Rho | Odds ratio (95% CI) | P | Sigma | Rho | Odds ratio (95% CI) | P | Sigma | Rho |
| None (Ref) |  |  | 0.96 | 0.22 |  |  | 1.09 | 0.27 |  |  | 1.25 | 0.32 |  |  | 1.02 | 0.24 |
| Phy | 3.68 (3.28 to 4.14) | <0.001 |  |  | 2.77 (2.14 to 3.57) | <0.001 |  |  | 2.45 (1.74 to 3.46) | <0.001 |  |  | 2.49 (2.09 to 2.96) | <0.001 |  |  |
| Psy | 1.71 (1.27 to 2.30) | <0.001 |  |  | 1.93 (1.54 to 2.41) | <0.001 |  |  | 1.49 (0.85 to 2.60) | 0.165 |  |  | 1.10 (0.93 to 1.30) | 0.272 |  |  |
| Cog | 1.26 (0.37 to 4.29) | 0.710 |  |  | 0.93 (0.67 to 1.29) | 0.667 |  |  | 1.34 (0.42 to 4.28) | 0.620 |  |  | 1.07 (0.73 to 1.57) | 0.715 |  |  |
| Phy + Psy | 5.77 (5.05 to 6.60) | <0.001 |  |  | 4.54 (3.43 to 6.01) | <0.001 |  |  | 5.71 (3.28 to 9.96) | <0.001 |  |  | 3.29 (2.81 to 3.85) | <0.001 |  |  |
| Phy + Cog | 3.51 (2.67 to 4.61) | <0.001 |  |  | 2.55 (1.63 to 3.99) | <0.001 |  |  | 4.59 (1.58 to 13.34) | 0.005 |  |  | 2.42 (1.89 to 3.11) | <0.001 |  |  |
| Psy + Cog |  |  |  |  | 2.43 (1.80 to 3.29) | <0.001 |  |  |  |  |  |  | 1.35 (1.08 to 1.69) | 0.009 |  |  |
| Phy + Psy + Cog | 4.33 (2.90 to 6.48) | <0.001 |  |  | 6.59 (4.40 to 9.86) | <0.001 |  |  | 5.64 (1.37 to 23.25) | 0.017 |  |  | 3.66 (3.09 to 4.34) | <0.001 |  |  |

Table K. The association between multimorbidity and inpatient by country (continued).

| Multimorbidity | Mexico | | | | Austria | | | | Germany | | | | Sweden | | | |
| --- | --- | --- | --- | --- | --- | --- | --- | --- | --- | --- | --- | --- | --- | --- | --- | --- |
|  | Odds ratio (95% CI) | P | Sigma | Rho | Odds ratio (95% CI) | P | Sigma | Rho | Odds ratio (95% CI) | P | Sigma | Rho | Odds ratio (95% CI) | P | Sigma | Rho |
| None (Ref) |  |  | 1.14 | 0.28 |  |  | 0.83 | 0.17 |  |  | 0.75 | 0.15 |  |  | 0.65 | 0.11 |
| Phy | 2.54 (1.77 to 3.65) | <0.001 |  |  | 2.09 (1.80 to 2.43) | <0.001 |  |  | 2.44 (2.02 to 2.94) | <0.001 |  |  | 2.39 (1.93 to 2.96) | <0.001 |  |  |
| Psy | 1.56 (0.89 to 2.72) | 0.117 |  |  | 2.28 (1.71 to 3.05) | <0.001 |  |  | 1.68 (1.17 to 2.40) | 0.005 |  |  | 2.26 (1.54 to 3.31) | <0.001 |  |  |
| Cog | 1.39 (0.76 to 2.52) | 0.281 |  |  | 0.48 (0.13 to 1.73) | 0.263 |  |  | 1.41 (0.34 to 5.77) | 0.636 |  |  | 1.61 (0.32 to 8.04) | 0.564 |  |  |
| Phy + Psy | 4.66 (3.18 to 6.83) | <0.001 |  |  | 3.67 (3.06 to 4.41) | <0.001 |  |  | 4.74 (3.85 to 5.83) | <0.001 |  |  | 4.59 (3.58 to 5.88) | <0.001 |  |  |
| Phy + Cog | 2.59 (1.65 to 4.08) | <0.001 |  |  | 1.83 (0.90 to 3.70) | 0.096 |  |  | 3.91 (2.26 to 6.76) | <0.001 |  |  | 2.72 (1.16 to 6.38) | 0.021 |  |  |
| Psy + Cog | 2.94 (1.43 to 6.03) | 0.003 |  |  | 2.06 (0.61 to 7.02) | 0.247 |  |  | 3.01 (0.90 to 10.09) | 0.074 |  |  |  |  |  |  |
| Phy + Psy + Cog | 3.90 (2.51 to 6.05) | <0.001 |  |  | 3.34 (1.88 to 5.94) | <0.001 |  |  | 3.76 (2.11 to 6.70) | <0.001 |  |  | 5.06 (2.38 to 10.74) | <0.001 |  |  |

Table K. The association between multimorbidity and inpatient by country (continued).

| Multimorbidity | Netherlands | | | | Spain | | | | Italy | | | | France | | | |
| --- | --- | --- | --- | --- | --- | --- | --- | --- | --- | --- | --- | --- | --- | --- | --- | --- |
|  | Odds ratio (95% CI) | P | Sigma | Rho | Odds ratio (95% CI) | P | Sigma | Rho | Odds ratio (95% CI) | P | Sigma | Rho | Odds ratio (95% CI) | P | Sigma | Rho |
| None (Ref) |  |  | 0.81 | 0.17 |  |  | 0.98 | 0.23 |  |  | 0.78 | 0.16 |  |  | 0.75 | 0.15 |
| Phy | 3.06 (2.40 to 3.89) | <0.001 |  |  | 2.62 (2.06 to 3.33) | <0.001 |  |  | 2.63 (2.05 to 3.36) | <0.001 |  |  | 2.72 (2.23 to 3.31) | <0.001 |  |  |
| Psy | 2.07 (1.32 to 3.23) | 0.001 |  |  | 1.33 (0.82 to 2.16) | 0.247 |  |  | 2.05 (1.39 to 3.04) | <0.001 |  |  | 2.04 (1.51 to 2.74) | <0.001 |  |  |
| Cog | 0.80 (0.10 to 6.21) | 0.832 |  |  | 1.56 (0.71 to 3.43) | 0.272 |  |  | 0.85 (0.25 to 2.91) | 0.795 |  |  | 2.91 (1.40 to 6.04) | 0.004 |  |  |
| Phy + Psy | 5.87 (4.43 to 7.77) | <0.001 |  |  | 5.52 (4.25 to 7.17) | <0.001 |  |  | 5.98 (4.62 to 7.75) | <0.001 |  |  | 4.56 (3.71 to 5.61) | <0.001 |  |  |
| Phy + Cog | 4.99 (2.42 to 10.28) | <0.001 |  |  | 3.21 (2.13 to 4.84) | <0.001 |  |  | 3.56 (2.32 to 5.47) | <0.001 |  |  | 3.28 (2.01 to 5.36) | <0.001 |  |  |
| Psy + Cog |  |  |  |  | 1.39 (0.49 to 3.92) | 0.537 |  |  | 4.26 (2.05 to 8.88) | <0.001 |  |  | 3.76 (1.67 to 8.46) | 0.001 |  |  |
| Phy + Psy + Cog | 7.91 (3.51 to 17.83) | <0.001 |  |  | 7.61 (5.45 to 10.62) | <0.001 |  |  | 6.33 (4.53 to 8.83) | <0.001 |  |  | 7.05 (4.67 to 10.62) | <0.001 |  |  |

Table K. The association between multimorbidity and inpatient by country (continued).

| Multimorbidity | Denmark | | | | Greece | | | | Switzerland | | | | Belgium | | | |
| --- | --- | --- | --- | --- | --- | --- | --- | --- | --- | --- | --- | --- | --- | --- | --- | --- |
|  | Odds ratio (95% CI) | P | Sigma | Rho | Odds ratio (95% CI) | P | Sigma | Rho | Odds ratio (95% CI) | P | Sigma | Rho | Odds ratio (95% CI) | P | Sigma | Rho |
| None (Ref) |  |  | 0.75 | 0.14 |  |  | 0.96 | 0.22 |  |  | 0.79 | 0.16 |  |  | 0.91 | 0.20 |
| Phy | 2.48 (1.95 to 3.14) | <0.001 |  |  | 2.56 (1.73 to 3.78) | <0.001 |  |  | 2.45 (2.04 to 2.95) | <0.001 |  |  | 1.99 (1.69 to 2.35) | <0.001 |  |  |
| Psy | 3.34 (2.15 to 5.19) | <0.001 |  |  | 1.29 (0.66 to 2.51) | 0.461 |  |  | 1.27 (0.85 to 1.89) | 0.244 |  |  | 2.10 (1.61 to 2.75) | <0.001 |  |  |
| Cog |  |  |  |  | 1.48 (0.40 to 5.51) | 0.555 |  |  | 0.52 (0.08 to 3.46) | 0.496 |  |  | 1.61 (0.66 to 3.92) | 0.296 |  |  |
| Phy + Psy | 5.25 (3.99 to 6.91) | <0.001 |  |  | 6.21 (4.06 to 9.50) | <0.001 |  |  | 4.11 (3.27 to 5.17) | <0.001 |  |  | 3.19 (2.65 to 3.84) | <0.001 |  |  |
| Phy + Cog | 7.30 (3.24 to 16.43) | <0.001 |  |  | 3.24 (1.54 to 6.84) | 0.002 |  |  | 2.08 (0.70 to 6.23) | 0.190 |  |  | 1.50 (0.83 to 2.70) | 0.177 |  |  |
| Psy + Cog | 13.40 (1.55 to 115.55) | 0.018 |  |  | 0.70 (0.08 to 5.92) | 0.742 |  |  | 3.15 (0.20 to 49.04) | 0.413 |  |  | 2.39 (0.79 to 7.26) | 0.123 |  |  |
| Phy + Psy + Cog | 4.76 (1.57 to 14.45) | 0.006 |  |  | 8.28 (4.65 to 14.72) | <0.001 |  |  | 1.55 (0.53 to 4.57) | 0.427 |  |  | 4.24 (2.59 to 6.92) | <0.001 |  |  |

Table K. The association between multimorbidity and inpatient by country (continued).

| Multimorbidity | Israel | | | | Czech Republic | | | | Poland | | | | Luxembourg | | | |
| --- | --- | --- | --- | --- | --- | --- | --- | --- | --- | --- | --- | --- | --- | --- | --- | --- |
|  | Odds ratio (95% CI) | P | Sigma | Rho | Odds ratio (95% CI) | P | Sigma | Rho | Odds ratio (95% CI) | P | Sigma | Rho | Odds ratio (95% CI) | P | Sigma | Rho |
| None (Ref) |  |  | 0.84 | 0.18 |  |  | 0.79 | 0.16 |  |  | 0.71 | 0.13 |  |  | 0.47 | 0.06 |
| Phy | 3.63 (2.23 to 5.89) | <0.001 |  |  | 2.59 (2.15 to 3.11) | <0.001 |  |  | 3.01 (2.09 to 4.34) | <0.001 |  |  | 2.41 (1.73 to 3.36) | <0.001 |  |  |
| Psy | 2.95 (1.32 to 6.59) | 0.008 |  |  | 2.65 (1.90 to 3.70) | <0.001 |  |  | 2.11 (1.20 to 3.70) | 0.009 |  |  | 1.71 (0.94 to 3.14) | 0.080 |  |  |
| Cog |  |  |  |  | 1.18 (0.24 to 5.72) | 0.835 |  |  | 1.60 (0.32 to 7.97) | 0.566 |  |  | 2.39 (0.53 to 10.72) | 0.256 |  |  |
| Phy + Psy | 7.39 (4.28 to 12.76) | <0.001 |  |  | 4.49 (3.67 to 5.49) | <0.001 |  |  | 4.24 (2.91 to 6.19) | <0.001 |  |  | 3.91 (2.71 to 5.64) | <0.001 |  |  |
| Phy + Cog | 3.00 (1.05 to 8.59) | 0.040 |  |  | 3.01 (1.79 to 5.05) | <0.001 |  |  | 2.72 (1.38 to 5.35) | 0.004 |  |  | 2.88 (1.20 to 6.95) | 0.018 |  |  |
| Psy + Cog |  |  |  |  | 0.49 (0.08 to 3.23) | 0.462 |  |  |  |  |  |  | 1.31 (0.13 to 13.66) | 0.820 |  |  |
| Phy + Psy + Cog | 6.50 (2.91 to 14.54) | <0.001 |  |  | 7.80 (5.01 to 12.15) | <0.001 |  |  | 3.67 (2.10 to 6.39) | <0.001 |  |  | 4.16 (1.72 to 10.04) | 0.002 |  |  |

Table K. The association between multimorbidity and inpatient by country (continued).

| Multimorbidity | Hungary | | | | Portugal | | | | Slovenia | | | | Estonia | | | |
| --- | --- | --- | --- | --- | --- | --- | --- | --- | --- | --- | --- | --- | --- | --- | --- | --- |
|  | Odds ratio (95% CI) | P | Sigma | Rho | Odds ratio (95% CI) | P | Sigma | Rho | Odds ratio (95% CI) | P | Sigma | Rho | Odds ratio (95% CI) | P | Sigma | Rho |
| None (Ref) |  |  | 0.76 | 0.15 |  |  | 2.20 | 0.60 |  |  | 0.75 | 0.15 |  |  | 0.82 | 0.17 |
| Phy | 2.93 (2.00 to 4.30) | <0.001 |  |  | 2.99 (1.46 to 6.12) | 0.003 |  |  | 2.45 (2.00 to 2.99) | <0.001 |  |  | 2.49 (2.05 to 3.04) | <0.001 |  |  |
| Psy | 2.48 (1.30 to 4.72) | 0.006 |  |  | 0.80 (0.23 to 2.81) | 0.725 |  |  | 2.31 (1.66 to 3.22) | <0.001 |  |  | 1.83 (1.33 to 2.53) | <0.001 |  |  |
| Cog | 0.92 (0.09 to 8.85) | 0.940 |  |  | 0.76 (0.10 to 5.69) | 0.788 |  |  | 0.33 (0.04 to 2.80) | 0.310 |  |  | 0.83 (0.21 to 3.35) | 0.798 |  |  |
| Phy + Psy | 5.36 (3.56 to 8.07) | <0.001 |  |  | 6.18 (2.94 to 12.99) | <0.001 |  |  | 3.72 (2.96 to 4.67) | <0.001 |  |  | 4.31 (3.51 to 5.29) | <0.001 |  |  |
| Phy + Cog | 2.81 (1.08 to 7.34) | 0.035 |  |  | 2.97 (0.69 to 12.72) | 0.143 |  |  | 1.95 (0.95 to 3.97) | 0.068 |  |  | 3.06 (1.73 to 5.42) | <0.001 |  |  |
| Psy + Cog | 2.05 (0.32 to 12.95) | 0.446 |  |  | 0.95 (0.05 to 17.25) | 0.970 |  |  | 3.98 (1.04 to 15.24) | 0.044 |  |  | 2.77 (0.95 to 8.10) | 0.062 |  |  |
| Phy + Psy + Cog | 3.72 (2.00 to 6.90) | <0.001 |  |  | 8.02 (2.89 to 22.20) | <0.001 |  |  | 2.71 (1.53 to 4.80) | 0.001 |  |  | 4.84 (3.32 to 7.07) | <0.001 |  |  |

Table K. The association between multimorbidity and inpatient by country (continued).

| Multimorbidity | Croatia | | | | Lithuania | | | | | Bulgaria | | | | | Cyprus | | | | |
| --- | --- | --- | --- | --- | --- | --- | --- | --- | --- | --- | --- | --- | --- | --- | --- | --- | --- | --- | --- |
|  | Odds ratio (95% CI) | P | Sigma | Rho | Odds ratio (95% CI) | P | Sigma | Rho | Odds ratio (95% CI) | | P | Sigma | Rho | Odds ratio (95% CI) | | P | Sigma | Rho |  |
| None (Ref) |  |  | 0.64 | 0.11 |  |  | 0.01 | 0.00 |  | |  | 0.01 | 0.00 |  | |  | 0.01 | 0.00 |  |
| Phy | 2.36 (1.63 to 3.43) | <0.001 |  |  | 2.61 (1.55 to 4.40) | <0.001 |  |  | 2.48 (1.12 to 5.47) | | 0.025 |  |  | 1.39 (0.38 to 5.03) | | 0.620 |  |  |  |
| Psy | 2.65 (1.48 to 4.75) | 0.001 |  |  | 2.25 (0.92 to 5.48) | 0.076 |  |  | 1.59 (0.27 to 9.54) | | 0.609 |  |  |  | |  |  |  |  |
| Cog |  |  |  |  |  |  |  |  | 6.72 (2.90 to 15.56) | | <0.001 |  |  |  | |  |  |  |  |
| Phy + Psy | 3.81 (2.53 to 5.73) | <0.001 |  |  | 5.95 (3.50 to 10.12) | <0.001 |  |  | 2.59 (0.49 to 13.66) | | 0.261 |  |  | 2.22 (0.52 to 9.50) | | 0.282 |  |  |  |
| Phy + Cog | 2.05 (0.62 to 6.75) | 0.236 |  |  | 2.40 (0.62 to 9.26) | 0.202 |  |  |  | |  |  |  | 1.71 (0.28 to 10.50) | | 0.562 |  |  |  |
| Psy + Cog | 3.56 (0.60 to 21.24) | 0.164 |  |  |  |  |  |  | 3.46 (1.01 to 11.83) | | 0.048 |  |  |  | |  |  |  |  |
| Phy + Psy + Cog | 4.15 (2.05 to 8.40) | <0.001 |  |  | 9.52 (3.73 to 24.28) | <0.001 |  |  | 0.98 (0.96 to 1.01) | | 0.137 |  |  | 0.59 (0.05 to 6.99) | | 0.672 |  |  |  |

Table K. The association between multimorbidity and inpatient by country (continued).

| Multimorbidity | Finland | | | | Latvia | | | | Romania | | | | | Slovakia | | | | |
| --- | --- | --- | --- | --- | --- | --- | --- | --- | --- | --- | --- | --- | --- | --- | --- | --- | --- | --- |
|  | Odds ratio (95% CI) | P | Sigma | Rho | Odds ratio (95% CI) | P | Sigma | Rho | Odds ratio (95% CI) | P | Sigma | Rho | Odds ratio (95% CI) | | P | Sigma | Rho |  |
| None (Ref) |  |  | 0.01 | 0.00 |  |  | 0.03 | 0.00 |  |  | 0.47 | 0.06 |  | |  | 2.80 | 0.70 |  |
| Phy | 1.72 (0.92 to 3.20) | 0.087 |  |  | 1.93 (0.01 to 287.18) | 0.797 |  |  | 2.68 (0.06 to 127.45) | 0.617 |  |  | 6.13 (0.20 to 192.16) | | 0.302 |  |  |  |
| Psy | 0.83 (0.22 to 3.07) | 0.782 |  |  | 3.15 (0.00 to 3005.31) | 0.743 |  |  | 0.73 (0.13 to 4.20) | 0.724 |  |  | 3.49 (0.21 to 58.21) | | 0.384 |  |  |  |
| Cog |  |  |  |  | 2.73 (0.00 to 2.52e+07) | 0.902 |  |  | 5.72 (0.00 to 15493.72) | 0.666 |  |  |  | |  |  |  |  |
| Phy + Psy | 2.30 (1.19 to 4.43) | 0.013 |  |  | 3.10 (0.00 to 22349.38) | 0.803 |  |  | 6.49 (0.00 to 36707.45) | 0.671 |  |  | 67.04 (0.03 to 128966.04) | | 0.276 |  |  |  |
| Phy + Cog |  |  |  |  | 0.74 (0.06 to 9.57) | 0.818 |  |  | 4.73 (0.00 to 10052.78) | 0.691 |  |  | 13.84 (0.04 to 4314.39) | | 0.370 |  |  |  |
| Psy + Cog | 1.59 (0.16 to 16.25) | 0.696 |  |  |  |  |  |  | 2.21 (0.31 to 15.85) | 0.431 |  |  |  | |  |  |  |  |
| Phy + Psy + Cog | 1.03 (1.00 to 1.05) | 0.068 |  |  | 3.34 (0.00 to 300746.51) | 0.836 |  |  | 6.98 (0.00 to 81346.43) | 0.684 |  |  | 27.62 (0.05 to 14014.43) | | 0.296 |  |  |  |
| *Note:* Cog: cognitive disorder; Psy: psychological disorder; Phy: physical condition; Psy + Cog: psychological-cognitive multimorbidity; Phy + Cog: physical-cognitive multimorbidity; Phy + Psy: physical- psychological multimorbidity; Phy + Psy + Cog: physical-psychological-cognitive multimorbidity. Sigma (σᵤ) denotes the standard deviation of the individual-level random intercept, reflecting unobserved heterogeneity between individuals. Rho (ρ) represents the intra-class correlation coefficient, indicating the proportion of total variance attributable to stable between-individual differences. | | | | | | | | | | | | | | | | | |  |

Table L. The association between multimorbidity and number of inpatient admissions by country.

| Multimorbidity | United States | | | China | | | Japan | | | South Korea | | |
| --- | --- | --- | --- | --- | --- | --- | --- | --- | --- | --- | --- | --- |
|  | Incident rate ratio (95% CI) | P | r | Incident rate ratio (95% CI) | P | r | Incident rate ratio (95% CI) | P | r | Incident rate ratio (95% CI) | P | r |
| None (Ref) |  |  | 5.00 (4.71 to 5.31) |  |  | 4.41e+07 (3.92e+07 to 4.96e+07) |  |  | 260856.97 (0.00 to 9.4e+104) |  |  | 12.26 (10.59 to 14.18) |
| Phy | 3.22 (2.91 to 3.57) | <0.001 | 5.00 (4.71 to 5.31) | 2.35 (1.93 to 2.85) | <0.001 | 4.41e+07 (3.92e+07 to 4.96e+07) | 2.14 (1.70 to 2.71) | <0.001 | 260856.97 (0.00 to 9.4e+104) | 2.25 (1.93 to 2.63) | <0.001 | 12.26 (10.59 to 14.18) |
| Psy | 1.68 (1.30 to 2.17) | <0.001 | 5.00 (4.71 to 5.31) | 1.82 (1.51 to 2.19) | <0.001 | 4.41e+07 (3.92e+07 to 4.96e+07) | 1.42 (0.89 to 2.26) | 0.144 | 260856.97 (0.00 to 9.4e+104) | 1.11 (0.96 to 1.30) | 0.164 | 12.26 (10.59 to 14.18) |
| Cog | 1.23 (0.44 to 3.49) | 0.691 | 5.00 (4.71 to 5.31) | 0.95 (0.72 to 1.26) | 0.738 | 4.41e+07 (3.92e+07 to 4.96e+07) | 1.37 (0.51 to 3.73) | 0.531 | 260856.97 (0.00 to 9.4e+104) | 1.07 (0.76 to 1.50) | 0.713 | 12.26 (10.59 to 14.18) |
| Phy + Psy | 4.63 (4.14 to 5.17) | <0.001 | 5.00 (4.71 to 5.31) | 3.54 (2.92 to 4.30) | <0.001 | 4.41e+07 (3.92e+07 to 4.96e+07) | 4.07 (3.02 to 5.48) | <0.001 | 260856.97 (0.00 to 9.4e+104) | 3.01 (2.61 to 3.46) | <0.001 | 12.26 (10.59 to 14.18) |
| Phy + Cog | 3.32 (2.71 to 4.06) | <0.001 | 5.00 (4.71 to 5.31) | 2.37 (1.68 to 3.34) | <0.001 | 4.41e+07 (3.92e+07 to 4.96e+07) | 3.42 (1.66 to 7.04) | 0.001 | 260856.97 (0.00 to 9.4e+104) | 2.29 (1.86 to 2.82) | <0.001 | 12.26 (10.59 to 14.18) |
| Psy + Cog | 0.00 (0.00 to .) | 0.997 | 5.00 (4.71 to 5.31) | 2.17 (1.72 to 2.74) | <0.001 | 4.41e+07 (3.92e+07 to 4.96e+07) | 0.00 (0.00 to .) | 1.000 | 260856.97 (0.00 to 9.4e+104) | 1.41 (1.15 to 1.71) | 0.001 | 12.26 (10.59 to 14.18) |
| Phy + Psy + Cog | 3.94 (3.01 to 5.18) | <0.001 | 5.00 (4.71 to 5.31) | 4.55 (3.52 to 5.90) | <0.001 | 4.41e+07 (3.92e+07 to 4.96e+07) | 4.01 (1.62 to 9.90) | 0.003 | 260856.97 (0.00 to 9.4e+104) | 3.33 (2.87 to 3.87) | <0.001 | 12.26 (10.59 to 14.18) |

Table L. The association between multimorbidity and number of inpatient admissions by country (continued).

| Multimorbidity | Austria | | | Germany | | | Sweden | | | Netherlands | | |
| --- | --- | --- | --- | --- | --- | --- | --- | --- | --- | --- | --- | --- |
|  | Incident rate ratio (95% CI) | P | r | Incident rate ratio (95% CI) | P | r | Incident rate ratio (95% CI) | P | r | Incident rate ratio (95% CI) | P | r |
| None (Ref) |  |  | 12.26 (10.59 to 14.18) |  |  | 3.09e+07 (2.86e+07 to 3.34e+07) |  |  | 9.70e+07 (8.92e+07 to 1.05e+08) |  |  | 433682.95 (0.00 to 4.7e+110) |
| Phy | 2.25 (1.93 to 2.63) | <0.001 | 12.26 (10.59 to 14.18) | 1.91 (1.69 to 2.15) | <0.001 | 3.09e+07 (2.86e+07 to 3.34e+07) | 2.25 (1.93 to 2.63) | <0.001 | 9.70e+07 (8.92e+07 to 1.05e+08) | 2.29 (1.89 to 2.78) | <0.001 | 433682.95 (0.00 to 4.7e+110) |
| Psy | 1.11 (0.96 to 1.30) | 0.164 | 12.26 (10.59 to 14.18) | 2.00 (1.60 to 2.49) | <0.001 | 3.09e+07 (2.86e+07 to 3.34e+07) | 1.56 (1.17 to 2.09) | 0.003 | 9.70e+07 (8.92e+07 to 1.05e+08) | 2.12 (1.50 to 2.99) | <0.001 | 433682.95 (0.00 to 4.7e+110) |
| Cog | 1.07 (0.76 to 1.50) | 0.713 | 12.26 (10.59 to 14.18) | 0.59 (0.19 to 1.82) | 0.357 | 3.09e+07 (2.86e+07 to 3.34e+07) | 1.35 (0.43 to 4.23) | 0.601 | 9.70e+07 (8.92e+07 to 1.05e+08) | 1.55 (0.38 to 6.27) | 0.539 | 433682.95 (0.00 to 4.7e+110) |
| Phy + Psy | 3.01 (2.61 to 3.46) | <0.001 | 12.26 (10.59 to 14.18) | 3.06 (2.67 to 3.50) | <0.001 | 3.09e+07 (2.86e+07 to 3.34e+07) | 3.86 (3.27 to 4.55) | <0.001 | 9.70e+07 (8.92e+07 to 1.05e+08) | 4.02 (3.24 to 4.97) | <0.001 | 433682.95 (0.00 to 4.7e+110) |
| Phy + Cog | 2.29 (1.86 to 2.82) | <0.001 | 12.26 (10.59 to 14.18) | 1.67 (1.04 to 2.68) | 0.032 | 3.09e+07 (2.86e+07 to 3.34e+07) | 3.39 (2.32 to 4.98) | <0.001 | 9.70e+07 (8.92e+07 to 1.05e+08) | 2.72 (1.47 to 5.04) | 0.001 | 433682.95 (0.00 to 4.7e+110) |
| Psy + Cog | 1.41 (1.15 to 1.71) | 0.001 | 12.26 (10.59 to 14.18) | 1.75 (0.79 to 3.88) | 0.168 | 3.09e+07 (2.86e+07 to 3.34e+07) | 3.51 (1.38 to 8.96) | 0.008 | 9.70e+07 (8.92e+07 to 1.05e+08) | 0.00 (0.00 to .) | 1.000 | 433682.95 (0.00 to 4.7e+110) |
| Phy + Psy + Cog | 3.33 (2.87 to 3.87) | <0.001 | 12.26 (10.59 to 14.18) | 2.84 (2.03 to 3.99) | <0.001 | 3.09e+07 (2.86e+07 to 3.34e+07) | 3.12 (2.09 to 4.64) | <0.001 | 9.70e+07 (8.92e+07 to 1.05e+08) | 3.88 (2.32 to 6.47) | <0.001 | 433682.95 (0.00 to 4.7e+110) |

Table L. The association between multimorbidity and number of inpatient admissions by country (continued).

| Multimorbidity | Spain | | | Italy | | | France | | | Denmark | | |
| --- | --- | --- | --- | --- | --- | --- | --- | --- | --- | --- | --- | --- |
|  | Incident rate ratio (95% CI) | P | r | Incident rate ratio (95% CI) | P | r | Incident rate ratio (95% CI) | P | r | Incident rate ratio (95% CI) | P | r |
| None (Ref) |  |  | 726588.28 (0.00 to 1.2e+115) |  |  | 2.25e+07 (2.03e+07 to 2.48e+07) |  |  | 2.25e+06 (0.00 to 6.1e+123) |  |  | 5.07e+07 (4.64e+07 to 5.53e+07) |
| Phy | 2.87 (2.32 to 3.55) | <0.001 | 726588.28 (0.00 to 1.2e+115) | 2.44 (1.98 to 3.00) | <0.001 | 2.25e+07 (2.03e+07 to 2.48e+07) | 2.54 (2.03 to 3.17) | <0.001 | 2.25e+06 (0.00 to 6.1e+123) | 2.50 (2.09 to 2.99) | <0.001 | 5.07e+07 (4.64e+07 to 5.53e+07) |
| Psy | 2.02 (1.36 to 2.99) | <0.001 | 726588.28 (0.00 to 1.2e+115) | 1.34 (0.88 to 2.04) | 0.171 | 2.25e+07 (2.03e+07 to 2.48e+07) | 1.97 (1.37 to 2.83) | <0.001 | 2.25e+06 (0.00 to 6.1e+123) | 1.92 (1.47 to 2.52) | <0.001 | 5.07e+07 (4.64e+07 to 5.53e+07) |
| Cog | 0.90 (0.13 to 6.44) | 0.916 | 726588.28 (0.00 to 1.2e+115) | 1.51 (0.74 to 3.10) | 0.257 | 2.25e+07 (2.03e+07 to 2.48e+07) | 0.85 (0.27 to 2.67) | 0.776 | 2.25e+06 (0.00 to 6.1e+123) | 2.63 (1.43 to 4.85) | 0.002 | 5.07e+07 (4.64e+07 to 5.53e+07) |
| Phy + Psy | 5.03 (3.98 to 6.37) | <0.001 | 726588.28 (0.00 to 1.2e+115) | 4.57 (3.68 to 5.67) | <0.001 | 2.25e+07 (2.03e+07 to 2.48e+07) | 5.20 (4.15 to 6.51) | <0.001 | 2.25e+06 (0.00 to 6.1e+123) | 4.00 (3.33 to 4.82) | <0.001 | 5.07e+07 (4.64e+07 to 5.53e+07) |
| Phy + Cog | 4.11 (2.35 to 7.20) | <0.001 | 726588.28 (0.00 to 1.2e+115) | 2.99 (2.13 to 4.19) | <0.001 | 2.25e+07 (2.03e+07 to 2.48e+07) | 3.38 (2.34 to 4.88) | <0.001 | 2.25e+06 (0.00 to 6.1e+123) | 2.92 (1.99 to 4.31) | <0.001 | 5.07e+07 (4.64e+07 to 5.53e+07) |
| Psy + Cog | 0.00 (0.00 to .) | 1.000 | 726588.28 (0.00 to 1.2e+115) | 1.34 (0.55 to 3.29) | 0.520 | 2.25e+07 (2.03e+07 to 2.48e+07) | 3.73 (2.05 to 6.81) | <0.001 | 2.25e+06 (0.00 to 6.1e+123) | 3.41 (1.80 to 6.48) | <0.001 | 5.07e+07 (4.64e+07 to 5.53e+07) |
| Phy + Psy + Cog | 6.22 (3.60 to 10.73) | <0.001 | 726588.28 (0.00 to 1.2e+115) | 5.72 (4.42 to 7.41) | <0.001 | 2.25e+07 (2.03e+07 to 2.48e+07) | 5.61 (4.24 to 7.41) | <0.001 | 2.25e+06 (0.00 to 6.1e+123) | 5.47 (4.06 to 7.36) | <0.001 | 5.07e+07 (4.64e+07 to 5.53e+07) |

Table L. The association between multimorbidity and number of inpatient admissions by country (continued).

| Multimorbidity | Greece | | | Switzerland | | | Belgium | | | Israel | | |
| --- | --- | --- | --- | --- | --- | --- | --- | --- | --- | --- | --- | --- |
|  | Incident rate ratio (95% CI) | P | r | Incident rate ratio (95% CI) | P | r | Incident rate ratio (95% CI) | P | r | Incident rate ratio (95% CI) | P | r |
| None (Ref) |  |  | 6.30e+06 (5.61e+06 to 7.07e+06) |  |  | 7.00e+06 (5.92e+06 to 8.27e+06) |  |  | 522118.69 (0.00 to 1.8e+212) |  |  | 5.49e+06 (5.06e+06 to 5.95e+06) |
| Phy | 2.38 (1.93 to 2.94) | <0.001 | 6.30e+06 (5.61e+06 to 7.07e+06) | 2.51 (1.78 to 3.55) | <0.001 | 7.00e+06 (5.92e+06 to 8.27e+06) | 2.25 (1.92 to 2.64) | <0.001 | 522118.69 (0.00 to 1.8e+212) | 1.88 (1.63 to 2.16) | <0.001 | 5.49e+06 (5.06e+06 to 5.95e+06) |
| Psy | 3.11 (2.16 to 4.47) | <0.001 | 6.30e+06 (5.61e+06 to 7.07e+06) | 1.29 (0.70 to 2.36) | 0.416 | 7.00e+06 (5.92e+06 to 8.27e+06) | 1.25 (0.87 to 1.79) | 0.237 | 522118.69 (0.00 to 1.8e+212) | 1.97 (1.58 to 2.46) | <0.001 | 5.49e+06 (5.06e+06 to 5.95e+06) |
| Cog | 0.00 (0.00 to .) | 1.000 | 6.30e+06 (5.61e+06 to 7.07e+06) | 1.44 (0.45 to 4.66) | 0.540 | 7.00e+06 (5.92e+06 to 8.27e+06) | 0.55 (0.08 to 3.90) | 0.546 | 522118.69 (0.00 to 1.8e+212) | 1.52 (0.72 to 3.20) | 0.274 | 5.49e+06 (5.06e+06 to 5.95e+06) |
| Phy + Psy | 4.56 (3.62 to 5.74) | <0.001 | 6.30e+06 (5.61e+06 to 7.07e+06) | 5.09 (3.56 to 7.28) | <0.001 | 7.00e+06 (5.92e+06 to 8.27e+06) | 3.50 (2.92 to 4.20) | <0.001 | 522118.69 (0.00 to 1.8e+212) | 2.78 (2.39 to 3.22) | <0.001 | 5.49e+06 (5.06e+06 to 5.95e+06) |
| Phy + Cog | 4.80 (2.80 to 8.23) | <0.001 | 6.30e+06 (5.61e+06 to 7.07e+06) | 2.70 (1.48 to 4.93) | 0.001 | 7.00e+06 (5.92e+06 to 8.27e+06) | 1.90 (0.79 to 4.62) | 0.154 | 522118.69 (0.00 to 1.8e+212) | 1.42 (0.90 to 2.25) | 0.132 | 5.49e+06 (5.06e+06 to 5.95e+06) |
| Psy + Cog | 7.08 (1.83 to 27.43) | 0.005 | 6.30e+06 (5.61e+06 to 7.07e+06) | 0.78 (0.11 to 5.72) | 0.811 | 7.00e+06 (5.92e+06 to 8.27e+06) | 4.48 (0.65 to 30.59) | 0.126 | 522118.69 (0.00 to 1.8e+212) | 2.11 (0.88 to 5.05) | 0.095 | 5.49e+06 (5.06e+06 to 5.95e+06) |
| Phy + Psy + Cog | 3.99 (1.86 to 8.56) | <0.001 | 6.30e+06 (5.61e+06 to 7.07e+06) | 6.26 (4.03 to 9.72) | <0.001 | 7.00e+06 (5.92e+06 to 8.27e+06) | 1.45 (0.59 to 3.55) | 0.415 | 522118.69 (0.00 to 1.8e+212) | 3.28 (2.38 to 4.53) | <0.001 | 5.49e+06 (5.06e+06 to 5.95e+06) |

Table L. The association between multimorbidity and number of inpatient admissions by country (continued).

| Multimorbidity | Czech Republic | | | Poland | | | Luxembourg | | | Hungary | | |
| --- | --- | --- | --- | --- | --- | --- | --- | --- | --- | --- | --- | --- |
|  | Incident rate ratio (95% CI) | P | r | Incident rate ratio (95% CI) | P | r | Incident rate ratio (95% CI) | P | r | Incident rate ratio (95% CI) | P | r |
| None (Ref) |  |  | 6.80e+06 (5.65e+06 to 8.18e+06) |  |  | 1.39e+07 (1.28e+07 to 1.50e+07) |  |  | 680009.77 (0.00 to 4.9e+228) |  |  | 680009.77 (0.00 to 4.9e+228) |
| Phy | 3.28 (2.12 to 5.06) | <0.001 | 6.80e+06 (5.65e+06 to 8.18e+06) | 2.37 (2.02 to 2.79) | <0.001 | 1.39e+07 (1.28e+07 to 1.50e+07) | 2.77 (1.99 to 3.85) | <0.001 | 680009.77 (0.00 to 4.9e+228) | 2.77 (1.99 to 3.85) | <0.001 | 680009.77 (0.00 to 4.9e+228) |
| Psy | 2.79 (1.39 to 5.60) | 0.004 | 6.80e+06 (5.65e+06 to 8.18e+06) | 2.43 (1.84 to 3.21) | <0.001 | 1.39e+07 (1.28e+07 to 1.50e+07) | 2.01 (1.22 to 3.33) | 0.007 | 680009.77 (0.00 to 4.9e+228) | 2.01 (1.22 to 3.33) | 0.007 | 680009.77 (0.00 to 4.9e+228) |
| Cog | 0.00 (0.00 to .) | 1.000 | 6.80e+06 (5.65e+06 to 8.18e+06) | 1.22 (0.30 to 4.91) | 0.777 | 1.39e+07 (1.28e+07 to 1.50e+07) | 1.52 (0.37 to 6.29) | 0.564 | 680009.77 (0.00 to 4.9e+228) | 1.52 (0.37 to 6.29) | 0.564 | 680009.77 (0.00 to 4.9e+228) |
| Phy + Psy | 6.09 (3.87 to 9.58) | <0.001 | 6.80e+06 (5.65e+06 to 8.18e+06) | 3.82 (3.22 to 4.54) | <0.001 | 1.39e+07 (1.28e+07 to 1.50e+07) | 3.74 (2.68 to 5.22) | <0.001 | 680009.77 (0.00 to 4.9e+228) | 3.74 (2.68 to 5.22) | <0.001 | 680009.77 (0.00 to 4.9e+228) |
| Phy + Cog | 2.82 (1.20 to 6.63) | 0.018 | 6.80e+06 (5.65e+06 to 8.18e+06) | 2.68 (1.80 to 4.00) | <0.001 | 1.39e+07 (1.28e+07 to 1.50e+07) | 2.48 (1.42 to 4.35) | 0.001 | 680009.77 (0.00 to 4.9e+228) | 2.48 (1.42 to 4.35) | 0.001 | 680009.77 (0.00 to 4.9e+228) |
| Psy + Cog | 0.00 (0.00 to .) | 1.000 | 6.80e+06 (5.65e+06 to 8.18e+06) | 0.59 (0.08 to 4.11) | 0.594 | 1.39e+07 (1.28e+07 to 1.50e+07) | 0.00 (0.00 to .) | 1.000 | 680009.77 (0.00 to 4.9e+228) | 0.00 (0.00 to .) | 1.000 | 680009.77 (0.00 to 4.9e+228) |
| Phy + Psy + Cog | 5.83 (3.16 to 10.77) | <0.001 | 6.80e+06 (5.65e+06 to 8.18e+06) | 5.59 (4.16 to 7.51) | <0.001 | 1.39e+07 (1.28e+07 to 1.50e+07) | 3.35 (2.15 to 5.22) | <0.001 | 680009.77 (0.00 to 4.9e+228) | 3.35 (2.15 to 5.22) | <0.001 | 680009.77 (0.00 to 4.9e+228) |

Table L. The association between multimorbidity and number of inpatient admissions by country (continued).

| Multimorbidity | Portugal | | | Slovenia | | | Estonia | | | Croatia | | |
| --- | --- | --- | --- | --- | --- | --- | --- | --- | --- | --- | --- | --- |
|  | Incident rate ratio (95% CI) | P | r | Incident rate ratio (95% CI) | P | r | Incident rate ratio (95% CI) | P | r | Incident rate ratio (95% CI) | P | r |
| None (Ref) |  |  | 431192.02 (0.00 to 2.5e+235) |  |  | 7.06e+06 (6.04e+06 to 8.24e+06) |  |  | 2.92e+06 (0.00 to 8.6e+172) |  |  | 5.23e+06 (4.70e+06 to 5.81e+06) |
| Phy | 2.28 (1.67 to 3.11) | <0.001 | 431192.02 (0.00 to 2.5e+235) | 2.63 (1.85 to 3.73) | <0.001 | 7.06e+06 (6.04e+06 to 8.24e+06) | 2.12 (1.30 to 3.46) | 0.003 | 2.92e+06 (0.00 to 8.6e+172) | 2.28 (1.91 to 2.72) | <0.001 | 5.23e+06 (4.70e+06 to 5.81e+06) |
| Psy | 1.74 (1.00 to 3.02) | 0.048 | 431192.02 (0.00 to 2.5e+235) | 2.38 (1.36 to 4.14) | 0.002 | 7.06e+06 (6.04e+06 to 8.24e+06) | 0.83 (0.34 to 2.07) | 0.694 | 2.92e+06 (0.00 to 8.6e+172) | 2.10 (1.58 to 2.80) | <0.001 | 5.23e+06 (4.70e+06 to 5.81e+06) |
| Cog | 2.05 (0.64 to 6.60) | 0.228 | 431192.02 (0.00 to 2.5e+235) | 0.94 (0.13 to 6.85) | 0.948 | 7.06e+06 (6.04e+06 to 8.24e+06) | 0.44 (0.06 to 3.31) | 0.427 | 2.92e+06 (0.00 to 8.6e+172) | 0.35 (0.05 to 2.49) | 0.293 | 5.23e+06 (4.70e+06 to 5.81e+06) |
| Phy + Psy | 3.52 (2.54 to 4.87) | <0.001 | 431192.02 (0.00 to 2.5e+235) | 4.41 (3.09 to 6.28) | <0.001 | 7.06e+06 (6.04e+06 to 8.24e+06) | 3.28 (2.00 to 5.39) | <0.001 | 2.92e+06 (0.00 to 8.6e+172) | 3.22 (2.65 to 3.92) | <0.001 | 5.23e+06 (4.70e+06 to 5.81e+06) |
| Phy + Cog | 2.82 (1.39 to 5.73) | 0.004 | 431192.02 (0.00 to 2.5e+235) | 2.63 (1.17 to 5.91) | 0.019 | 7.06e+06 (6.04e+06 to 8.24e+06) | 1.65 (0.61 to 4.42) | 0.321 | 2.92e+06 (0.00 to 8.6e+172) | 1.79 (1.01 to 3.16) | 0.046 | 5.23e+06 (4.70e+06 to 5.81e+06) |
| Psy + Cog | 1.36 (0.19 to 9.90) | 0.759 | 431192.02 (0.00 to 2.5e+235) | 1.81 (0.43 to 7.60) | 0.415 | 7.06e+06 (6.04e+06 to 8.24e+06) | 0.96 (0.13 to 7.26) | 0.972 | 2.92e+06 (0.00 to 8.6e+172) | 3.26 (1.21 to 8.78) | 0.020 | 5.23e+06 (4.70e+06 to 5.81e+06) |
| Phy + Psy + Cog | 3.54 (1.85 to 6.78) | <0.001 | 431192.02 (0.00 to 2.5e+235) | 3.37 (2.01 to 5.65) | <0.001 | 7.06e+06 (6.04e+06 to 8.24e+06) | 4.14 (2.21 to 7.73) | <0.001 | 2.92e+06 (0.00 to 8.6e+172) | 2.53 (1.61 to 3.98) | <0.001 | 5.23e+06 (4.70e+06 to 5.81e+06) |

Table L. The association between multimorbidity and number of inpatient admissions by country (continued).

| Multimorbidity | Lithuania | | | Bulgaria | | | Cyprus | | | Finland | | |
| --- | --- | --- | --- | --- | --- | --- | --- | --- | --- | --- | --- | --- |
|  | Incident rate ratio (95% CI) | P | r | Incident rate ratio (95% CI) | P | r | Incident rate ratio (95% CI) | P | r | Incident rate ratio (95% CI) | P | r |
| None (Ref) |  |  | 9.14e+06 (0.00 to 6.9e+139) |  |  | 899260.44 (749770.21 to 1.08e+06) |  |  | 590880.91 (0.00 to 3.1e+185) |  |  | 2.37e+07 (1.71e+07 to 3.30e+07) |
| Phy | 2.35 (1.97 to 2.81) | <0.001 | 9.14e+06 (0.00 to 6.9e+139) | 2.26 (1.62 to 3.16) | <0.001 | 899260.44 (749770.21 to 1.08e+06) | 2.48 (1.51 to 4.08) | <0.001 | 590880.91 (0.00 to 3.1e+185) | 2.41 (1.13 to 5.15) | 0.023 | 2.37e+07 (1.71e+07 to 3.30e+07) |
| Psy | 1.74 (1.30 to 2.31) | <0.001 | 9.14e+06 (0.00 to 6.9e+139) | 2.60 (1.56 to 4.35) | <0.001 | 899260.44 (749770.21 to 1.08e+06) | 2.15 (0.94 to 4.94) | 0.070 | 590880.91 (0.00 to 3.1e+185) | 1.68 (0.34 to 8.18) | 0.522 | 2.37e+07 (1.71e+07 to 3.30e+07) |
| Cog | 0.96 (0.24 to 3.86) | 0.951 | 9.14e+06 (0.00 to 6.9e+139) | 0.00 (0.00 to .) | 1.000 | 899260.44 (749770.21 to 1.08e+06) | 0.00 (0.00 to .) | 1.000 | 590880.91 (0.00 to 3.1e+185) | 5.83 (2.63 to 12.94) | <0.001 | 2.37e+07 (1.71e+07 to 3.30e+07) |
| Phy + Psy | 3.79 (3.16 to 4.54) | <0.001 | 9.14e+06 (0.00 to 6.9e+139) | 3.43 (2.42 to 4.88) | <0.001 | 899260.44 (749770.21 to 1.08e+06) | 4.99 (3.04 to 8.18) | <0.001 | 590880.91 (0.00 to 3.1e+185) | 2.44 (0.51 to 11.72) | 0.267 | 2.37e+07 (1.71e+07 to 3.30e+07) |
| Phy + Cog | 2.80 (1.77 to 4.43) | <0.001 | 9.14e+06 (0.00 to 6.9e+139) | 2.07 (0.74 to 5.80) | 0.166 | 899260.44 (749770.21 to 1.08e+06) | 2.35 (0.69 to 8.00) | 0.170 | 590880.91 (0.00 to 3.1e+185) | 0.00 (0.00 to .) | 1.000 | 2.37e+07 (1.71e+07 to 3.30e+07) |
| Psy + Cog | 2.76 (1.14 to 6.72) | 0.025 | 9.14e+06 (0.00 to 6.9e+139) | 3.60 (0.88 to 14.74) | 0.074 | 899260.44 (749770.21 to 1.08e+06) | 0.00 (0.00 to .) | 1.000 | 590880.91 (0.00 to 3.1e+185) | 3.30 (1.08 to 10.14) | 0.037 | 2.37e+07 (1.71e+07 to 3.30e+07) |
| Phy + Psy + Cog | 4.10 (3.06 to 5.49) | <0.001 | 9.14e+06 (0.00 to 6.9e+139) | 3.69 (2.09 to 6.50) | <0.001 | 899260.44 (749770.21 to 1.08e+06) | 7.36 (3.43 to 15.78) | <0.001 | 590880.91 (0.00 to 3.1e+185) | 0.98 (0.96 to 1.01) | 0.139 | 2.37e+07 (1.71e+07 to 3.30e+07) |

Table L. The association between multimorbidity and number of inpatient admissions by country (continued).

| Multimorbidity | Latvia | | | Romania | | | Slovakia | | |
| --- | --- | --- | --- | --- | --- | --- | --- | --- | --- |
|  | Incident rate ratio (95% CI) | P | r | Incident rate ratio (95% CI) | P | r | Incident rate ratio (95% CI) | P | r |
| None (Ref) |  |  | 274962.53 (0.00 to 5.6e+278) |  |  | 490149.34 (0.00 to 1.7e+230) |  |  | 1.02e+06 (0.00 to .) |
| Phy | 1.38 (0.39 to 4.94) | 0.618 | 274962.53 (0.00 to 5.6e+278) | 1.68 (0.94 to 3.03) | 0.082 | 490149.34 (0.00 to 1.7e+230) | 1.87 (0.87 to 4.02) | 0.109 | 1.02e+06 (0.00 to .) |
| Psy | 0.00 (0.00 to .) | 1.000 | 274962.53 (0.00 to 5.6e+278) | 0.89 (0.25 to 3.10) | 0.852 | 490149.34 (0.00 to 1.7e+230) | 2.97 (0.89 to 9.96) | 0.078 | 1.02e+06 (0.00 to .) |
| Cog | 0.00 (0.00 to .) | 1.000 | 274962.53 (0.00 to 5.6e+278) | 4.93 (0.62 to 39.21) | 0.131 | 490149.34 (0.00 to 1.7e+230) | 2.24 (0.27 to 18.75) | 0.456 | 1.02e+06 (0.00 to .) |
| Phy + Psy | 2.27 (0.57 to 9.11) | 0.247 | 274962.53 (0.00 to 5.6e+278) | 2.27 (1.22 to 4.26) | 0.010 | 490149.34 (0.00 to 1.7e+230) | 2.75 (1.24 to 6.09) | 0.013 | 1.02e+06 (0.00 to .) |
| Phy + Cog | 1.62 (0.30 to 8.64) | 0.573 | 274962.53 (0.00 to 5.6e+278) | 0.00 (0.00 to .) | 1.000 | 490149.34 (0.00 to 1.7e+230) | 0.77 (0.09 to 6.28) | 0.804 | 1.02e+06 (0.00 to .) |
| Psy + Cog | 0.00 (0.00 to .) | 1.000 | 274962.53 (0.00 to 5.6e+278) | 2.35 (0.33 to 16.64) | 0.393 | 490149.34 (0.00 to 1.7e+230) | 0.00 (0.00 to .) | 1.000 | 1.02e+06 (0.00 to .) |
| Phy + Psy + Cog | 0.60 (0.06 to 6.41) | 0.671 | 274962.53 (0.00 to 5.6e+278) | 1.03 (1.00 to 1.05) | 0.030 | 490149.34 (0.00 to 1.7e+230) | 3.25 (1.01 to 10.41) | 0.047 | 1.02e+06 (0.00 to .) |
| *Note:* Cog: cognitive disorder; Psy: psychological disorder; Phy: physical condition; Psy + Cog: psychological-cognitive multimorbidity; Phy + Cog: physical-cognitive multimorbidity; Phy + Psy: physical- psychological multimorbidity; Phy + Psy + Cog: physical-psychological-cognitive multimorbidity. These are estimated by random effect negative binomial regression model. The dispersion parameter (r) from the negative binomial model was significantly greater than zero across countries, indicating overdispersion and justifying the use of negative binomial rather than Poisson models. | | | | | | | | | |

Table M. The meta-analysis details.

| Outcome | Multimorbidity | OR/IRR | Tau | H^2^ | I^2^ |
| --- | --- | --- | --- | --- | --- |
| Outpatient | Phy | 5.15 (4.46 to 5.85) | 2.89 | 9.89 | 89.88 |
|  | Psy | 1.58 (1.40 to 1.77) | 0.13 | 3.08 | 67.49 |
|  | Cog | 0.53 (0.39 to 0.67) | 0.06 | 2.41 | 58.42 |
|  | Phy + Psy | 7.80 (6.62 to 8.97) | 6.53 | 8.82 | 88.66 |
|  | Phy + Cog | 2.49 (1.93 to 3.05) | 0.79 | 2.09 | 52.09 |
|  | Psy + Cog | 0.90 (0.54 to 1.25) | 0.31 | 4.14 | 75.85 |
|  | Phy + Psy + Cog | 3.08 (2.22 to 3.94) | 2.28 | 188.67 | 99.47 |
| Inpatient | Phy | 2.54 (2.37 to 2.71) | 0.09 | 1.85 | 45.84 |
|  | Psy | 1.77 (1.57 to 1.97) | 0.12 | 1.97 | 49.28 |
|  | Cog | 0.96 (0.77 to 1.14) | 0.00 | 1.00 | 0.00 |
|  | Phy + Psy | 4.42 (4.04 to 4.79) | 0.52 | 2.42 | 58.72 |
|  | Phy + Cog | 2.63 (2.28 to 2.97) | 0.13 | 1.19 | 16.08 |
|  | Psy + Cog | 1.80 (1.27 to 2.34) | 0.36 | 1.58 | 36.69 |
|  | Phy + Psy + Cog | 4.17 (3.32 to 5.02) | 3.38 | 624.09 | 99.84 |
| Number of outpatient visits | Phy | 2.02 (1.87 to 2.17) | 0.16 | 41.61 | 97.60 |
|  | Psy | 1.40 (1.33 to 1.46) | 0.02 | 5.04 | 80.17 |
|  | Cog | 0.89 (0.75 to 1.04) | 0.12 | 5.83 | 82.84 |
|  | Phy + Psy | 2.55 (2.38 to 2.72) | 0.20 | 26.96 | 96.29 |
|  | Phy + Cog | 1.89 (1.75 to 2.03) | 0.09 | 4.16 | 75.98 |
|  | Psy + Cog | 1.39 (1.19 to 1.59) | 0.14 | 3.34 | 70.08 |
|  | Phy + Psy + Cog | 2.21 (1.99 to 2.43) | 0.30 | 595.38 | 99.83 |
| Number of inpatient admissions | Phy | 2.35 (2.21 to 2.49) | 0.07 | 2.03 | 50.62 |
|  | Psy | 1.66 (1.42 to 1.91) | 0.31 | 9.10 | 89.01 |
|  | Cog | 0.67 (0.40 to 0.94) | 0.28 | - | 100.00 |
|  | Phy + Psy | 3.79 (3.51 to 4.07) | 0.30 | 2.66 | 62.38 |
|  | Phy + Cog | 2.28 (1.84 to 2.72) | 0.94 | - | 100.00 |
|  | Psy + Cog | 0.88 (0.47 to 1.29) | 0.80 | - | 100.00 |
|  | Phy + Psy + Cog | 3.65 (3.04 to 4.26) | 1.97 | 481.06 | 99.79 |

*Note:* Cog: cognitive disorder; Psy: psychological disorder; Phy: physical condition; Psy + Cog: psychological-cognitive multimorbidity; Phy + Cog: physical-cognitive multimorbidity; Phy + Psy: physical- psychological multimorbidity; Phy + Psy + Cog: physical-psychological-cognitive multimorbidity. Between-study heterogeneity was estimated using the algorithm of the restricted maximum likelihood and was assessed using I^2^ and H^2^ statistics. I^2^ is the percentage of variability in the effect size that is caused by between-study heterogeneity, rather than by sampling error. The H^2^ statistic describes the ratio of the observed variation and the expected variance due to sampling error. For outpatient and inpatient, the estimated coefficients are OR. For Number of outpatient visits and Number of inpatient admissions, the estimated coefficients are IRR.

Table N. The statistical test results of multimorbidity*health insurance.

| Outcome | Interaction term | | Measure | Value | P |
| --- | --- | --- | --- | --- | --- |
| Outpatient | Health Insurance * | Phy | OR | 0.69 | <0.001 |
|  |  | Psy |  | 0.72 | <0.001 |
|  |  | Cog |  | 1.12 | 0.230 |
|  |  | Phy + Psy |  | 0.44 | <0.001 |
|  |  | Phy + Cog |  | 0.81 | 0.025 |
|  |  | Psy + Cog |  | 0.75 | 0.007 |
|  |  | Phy + Psy + Cog |  | 0.66 | <0.001 |
| Inpatient |  | Phy |  | 1.13 | 0.010 |
|  |  | Psy |  | 0.70 | <0.001 |
|  |  | Cog |  | 0.68 | 0.016 |
|  |  | Phy + Psy |  | 0.90 | 0.039 |
|  |  | Phy + Cog |  | 0.95 | 0.584 |
|  |  | Psy + Cog |  | 0.76 | 0.036 |
|  |  | Phy + Psy + Cog |  | 0.78 | <0.001 |
| Number of outpatient visits |  | Phy | IRR | 1.20 | <0.001 |
|  |  | Psy |  | 0.66 | <0.001 |
|  |  | Cog |  | 1.11 | 0.026 |
|  |  | Phy + Psy |  | 0.94 | <0.001 |
|  |  | Phy + Cog |  | 1.13 | <0.001 |
|  |  | Psy + Cog |  | 0.72 | <0.001 |
|  |  | Phy + Psy + Cog |  | 1.02 | 0.531 |
| Number of inpatient admissions |  | Phy |  | 1.14 | 0.013 |
|  |  | Psy |  | 0.80 | 0.001 |
|  |  | Cog |  | 0.64 | 0.008 |
|  |  | Phy + Psy |  | 0.92 | 0.155 |
|  |  | Phy + Cog |  | 0.92 | 0.462 |
|  |  | Psy + Cog |  | 0.75 | 0.055 |
|  |  | Phy + Psy + Cog |  | 0.78 | 0.001 |
| *Note:* Cog: cognitive disorder; Psy: psychological disorder; Phy: physical condition; Psy + Cog: psychological-cognitive multimorbidity; Phy + Cog: physical-cognitive multimorbidity; Phy + Psy: physical- psychological multimorbidity; Phy + Psy + Cog: physical-psychological-cognitive multimorbidity. | | | | | |

Table O. Association between each disorder domain and outpatient by country.

| Country | Physical | Psycho | Cognitive |
| --- | --- | --- | --- |
| United States | 6[5.27, 6.84] | 1.33[1.16, 1.52] | 0.39[0.28, 0.54] |
| China | 2.03[1.75, 2.36] | 2.15[1.89, 2.45] | 0.99[0.85, 1.15] |
| Japan | 6.9[4.59, 10.36] | 1.85[1.37, 2.5] | 0.95[0.54, 1.66] |
| South Korea | 2.6[2.42, 2.8] | 0.83[0.77, 0.89] | 1.11[1.02, 1.21] |
| Mexico | 3.73[3.17, 4.4] | 1.43[1.23, 1.66] | 0.75[0.63, 0.9] |
| Austria | 3.16[2.61, 3.83] | 2[1.57, 2.56] | 0.51[0.28, 0.92] |
| Germany | 7.26[5.34, 9.88] | 2.14[1.57, 2.93] | 0.33[0.16, 0.7] |
| Sweden | 4.89[4.09, 5.84] | 1.85[1.5, 2.27] | 0.72[0.36, 1.41] |
| Netherlands | 5.77[4.72, 7.04] | 2.4[1.86, 3.09] | 1.98[0.9, 4.37] |
| Spain | 5.28[4.33, 6.43] | 2.48[1.99, 3.1] | 0.9[0.66, 1.22] |
| Italy | 3.86[3.23, 4.6] | 1.92[1.62, 2.28] | 0.89[0.67, 1.18] |
| France | 9.07[6.97, 11.82] | 1.92[1.49, 2.47] | 0.54[0.29, 1] |
| Denmark | 5.47[4.48, 6.68] | 2.71[2.05, 3.58] | 0.76[0.26, 2.24] |
| Greece | 5.78[4.55, 7.34] | 1.28[1.03, 1.6] | 1.06[0.73, 1.54] |
| Switzerland | 5.12[4.17, 6.28] | 2.68[2.06, 3.48] | 1.4[0.6, 3.23] |
| Belgium | 6.43[5.03, 8.22] | 2.21[1.71, 2.86] | 0.7[0.34, 1.45] |
| Israel | 3.98[2.6, 6.11] | 2.49[1.52, 4.05] | 0.47[0.2, 1.07] |
| Czech Republic | 7.15[5.68, 9] | 1.26[0.99, 1.59] | 0.94[0.48, 1.86] |
| Poland | 11.14[7.64, 16.23] | 1.72[1.3, 2.28] | 0.82[0.49, 1.37] |
| Luxembourg | 5.74[3.11, 10.59] | 3.13[1.63, 6.01] | 0.59[0.17, 2.05] |
| Hungary | 11.02[6.38, 19.03] | 2.55[1.78, 3.65] | 1.25[0.62, 2.49] |
| Portugal | 2.71[1.97, 3.73] | 2.01[0, 903.54] | 1.19[0.02, 67.2] |
| Slovenia | 6.34[5.18, 7.76] | 1.63[1.32, 2.02] | 0.47[0.28, 0.8] |
| Estonia | 6.7[5.68, 7.89] | 1.91[1.66, 2.2] | 0.66[0.47, 0.94] |
| Croatia | 5.98[4.41, 8.11] | 2.18[1.6, 2.98] | 0.66[0.34, 1.29] |
| Lithuania | 10.71[5.65, 20.32] | 2.21[1, 4.87] | 0.52[0.25, 1.09] |
| Bulgaria | 6.67[3.95, 11.28] | 2.15[1.08, 4.28] | 2.13[0.61, 7.37] |
| Cyprus | 2.16[0.69, 6.78] | 1.8[0.46, 7.05] | 0.23[0.04, 1.18] |
| Finland | 3.46[1.83, 6.54] | 2.48[1.23, 5.03] | 1.38[0.06, 33.97] |
| Latvia | 27.62[8.92, 85.52] | 1.34[0.53, 3.42] | 0.54[0, 2498.95] |
| Romania | 6.57[0.08, 528.39] | 1.55[1.13, 2.12] | 0.62[0.39, 0.97] |
| Slovakia | 3.47[1.82, 6.6] | 2.46[1.19, 5.09] | 0.56[0.14, 2.17] |
| **Overall effects** | **5.20[4.51, 5.90]** | **1.87[1.68, 2.06]** | **0.71[0.60, 0.82]** |

Table P. Association between each disorder domain and inpatient by country.

| Country | Physical | Psycho | Cognitive |
| --- | --- | --- | --- |
| United States | 3.68[0.21, 3.3] | 1.63[1.51, 1.76] | 0.91[0.74, 1.12] |
| China | 2.77[0.25, 2.31] | 2.2[1.87, 2.6] | 1.18[0.98, 1.41] |
| Japan | 2.83[0.48, 2.02] | 2.22[1.57, 3.12] | 1.53[0.77, 3.04] |
| South Korea | 2.94[0.14, 2.67] | 1.35[1.25, 1.46] | 1.17[1.07, 1.27] |
| Mexico | 2.53[0.33, 1.96] | 1.92[1.55, 2.36] | 1.07[0.84, 1.37] |
| Austria | 2.09[0.14, 1.83] | 1.98[1.74, 2.25] | 0.92[0.62, 1.37] |
| Germany | 2.65[0.22, 2.24] | 2.01[1.78, 2.27] | 1.3[0.9, 1.88] |
| Sweden | 2.33[0.22, 1.93] | 2.04[1.75, 2.37] | 1.25[0.73, 2.14] |
| Netherlands | 3.22[0.35, 2.59] | 2.07[1.73, 2.48] | 1.46[0.89, 2.41] |
| Spain | 3.16[0.33, 2.57] | 2.25[1.95, 2.59] | 1.53[1.26, 1.86] |
| Italy | 2.98[0.3, 2.44] | 2.44[2.13, 2.79] | 1.35[1.11, 1.65] |
| France | 2.55[0.21, 2.17] | 1.84[1.64, 2.06] | 1.53[1.17, 1.99] |
| Denmark | 2.37[0.25, 1.93] | 2.34[1.98, 2.78] | 2.04[1.11, 3.75] |
| Greece | 3.34[0.56, 2.41] | 2.43[1.92, 3.06] | 1.51[1.07, 2.13] |
| Switzerland | 2.66[0.24, 2.23] | 1.76[1.5, 2.06] | 0.68[0.34, 1.35] |
| Belgium | 1.92[0.14, 1.66] | 1.78[1.59, 2] | 1.13[0.81, 1.57] |
| Israel | 3.43[0.73, 2.26] | 2.22[1.69, 2.93] | 0.98[0.58, 1.67] |
| Czech Republic | 2.49[0.2, 2.13] | 1.93[1.73, 2.16] | 1.55[1.14, 2.1] |
| Poland | 2.84[0.43, 2.11] | 1.54[1.29, 1.84] | 0.91[0.64, 1.28] |
| Luxembourg | 2.5[0.36, 1.88] | 1.75[1.39, 2.19] | 1.23[0.71, 2.14] |
| Hungary | 3.03[0.49, 2.2] | 2.05[1.64, 2.56] | 0.85[0.54, 1.35] |
| Portugal | 4.17[5.78, 0.27] | 2.22[1.4, 3.53] | 1.23[0.45, 3.34] |
| Slovenia | 2.27[0.2, 1.91] | 1.72[1.49, 1.98] | 0.85[0.57, 1.28] |
| Estonia | 2.64[0.22, 2.23] | 1.86[1.67, 2.07] | 1.29[0.99, 1.7] |
| Croatia | 2.28[0.36, 1.68] | 1.93[1.51, 2.47] | 1.06[0.64, 1.76] |
| Lithuania | 3.17[0.71, 2.04] | 2.55[1.92, 3.4] | 1.23[0.66, 2.3] |
| Bulgaria | 3[1.06, 1.5] | 2.49[1.62, 3.82] | 0.57[0.09, 3.48] |
| Cyprus | 2.09[1.34, 0.59] | 1.27[0.57, 2.81] | 0.62[0.19, 2.01] |
| Finland | 1.79[0.49, 1.04] | 1.32[0.9, 1.93] | 0.79[0.19, 3.33] |
| Latvia | 1.8[3.25, 0.05] | 1.82[0.02, 199.89] | 0.9[0.04, 22.47] |
| Romania | 3.06[3.19, 0.4] | 2.27[1.64, 3.13] | 1.69[1.07, 2.67] |
| Slovakia | 4969.15[0, 4969.15] | 6.86[3.47, 13.58] | 1.16[0.18, 7.25] |
| **Overall effects** | **2.62[2.47, 2.83]** | **1.93[1.82, 2.04]** | **1.15[1.05, 1.25]** |

Table Q. Association between each disorder domain and number of outpatient visits by country.

| Country | Physical | Psycho | Cognitive |
| --- | --- | --- | --- |
| United States | 1.89[1.82, 1.95] | 1.18[1.15, 1.22] | 0.76[0.69, 0.83] |
| China | 1.75[1.58, 1.94] | 1.92[1.74, 2.11] | 0.97[0.87, 1.09] |
| Japan | 3.33[3.07, 3.62] | 1.34[1.22, 1.48] | 1.11[0.89, 1.38] |
| South Korea | 1.9[1.85, 1.95] | 0.94[0.92, 0.96] | 1.03[1.01, 1.06] |
| Austria | 1.57[1.5, 1.65] | 1.35[1.29, 1.4] | 0.9[0.79, 1.03] |
| Germany | 1.72[1.64, 1.81] | 1.31[1.27, 1.36] | 1.06[0.93, 1.19] |
| Sweden | 1.82[1.72, 1.93] | 1.41[1.34, 1.48] | 0.88[0.72, 1.07] |
| Netherlands | 1.97[1.87, 2.08] | 1.46[1.38, 1.54] | 1.19[1.01, 1.4] |
| Spain | 1.85[1.77, 1.93] | 1.37[1.32, 1.42] | 1.01[0.96, 1.07] |
| Italy | 1.79[1.71, 1.87] | 1.32[1.28, 1.37] | 1.03[0.97, 1.1] |
| France | 1.77[1.7, 1.84] | 1.27[1.23, 1.3] | 1.04[0.97, 1.12] |
| Denmark | 1.93[1.83, 2.05] | 1.48[1.41, 1.56] | 1.19[0.96, 1.48] |
| Greece | 2.11[1.98, 2.25] | 1.12[1.06, 1.18] | 0.98[0.9, 1.07] |
| Switzerland | 1.82[1.73, 1.92] | 1.42[1.35, 1.49] | 1.44[1.18, 1.75] |
| Belgium | 1.73[1.66, 1.8] | 1.3[1.26, 1.35] | 1.12[1.02, 1.23] |
| Israel | 1.76[1.61, 1.93] | 1.44[1.33, 1.55] | 0.85[0.71, 1.01] |
| Czech Republic | 1.94[1.85, 2.02] | 1.25[1.21, 1.29] | 1.02[0.93, 1.13] |
| Poland | 2.8[2.57, 3.05] | 1.28[1.22, 1.35] | 0.89[0.8, 0.99] |
| Luxembourg | 1.63[1.5, 1.77] | 1.32[1.23, 1.41] | 0.93[0.76, 1.13] |
| Hungary | 2.51[2.3, 2.74] | 1.45[1.36, 1.54] | 1.13[0.99, 1.29] |
| Portugal | 1.65[1.49, 1.82] | 1.54[1.41, 1.69] | 1.07[0.93, 1.23] |
| Slovenia | 1.92[1.82, 2.02] | 1.24[1.19, 1.3] | 0.9[0.79, 1.03] |
| Estonia | 2.21[2.11, 2.32] | 1.32[1.28, 1.36] | 0.95[0.86, 1.04] |
| Croatia | 2.39[2.19, 2.6] | 1.43[1.34, 1.54] | 0.86[0.71, 1.03] |
| Lithuania | 2.31[2.01, 2.65] | 1.38[1.26, 1.52] | 0.9[0.7, 1.16] |
| Bulgaria | 2.39[1.97, 2.9] | 1.3[1.14, 1.49] | 0.94[0.72, 1.22] |
| Cyprus | 1.7[1.33, 2.17] | 1.32[1.06, 1.64] | 0.85[0.63, 1.14] |
| Finland | 1.69[1.44, 1.98] | 1.45[1.27, 1.66] | 0.84[0.43, 1.66] |
| Latvia | 2.04[1.71, 2.44] | 1.27[1.12, 1.45] | 0.82[0.6, 1.12] |
| Romania | 3.24[2.74, 3.83] | 1.42[1.25, 1.6] | 0.88[0.71, 1.09] |
| Slovakia | 1.89[1.65, 2.16] | 1.75[1.53, 2] | 0.93[0.68, 1.28] |
| **Overall effects** | **2.01[1.87, 2.15]** | **1.35[1.30, 1.41]** | **0.98[0.94, 1.02]** |

Table R. Association between each disorder domain and number of inpatient admissions by country.

| Country | Physical | Psycho | Cognitive |
| --- | --- | --- | --- |
| United States | 3.19[2.9, 3.51] | 1.48[1.4, 1.56] | 0.99[0.85, 1.14] |
| China | 2.28[2.02, 2.58] | 1.94[1.72, 2.2] | 1.15[1, 1.32] |
| Japan | 2.37[1.92, 2.91] | 1.87[1.5, 2.35] | 1.4[0.86, 2.27] |
| South Korea | 2.62[2.42, 2.83] | 1.36[1.27, 1.45] | 1.16[1.08, 1.24] |
| Austria | 1.89[1.7, 2.1] | 1.75[1.61, 1.92] | 0.94[0.73, 1.2] |
| Germany | 2.37[2.07, 2.71] | 1.76[1.61, 1.93] | 1.24[0.97, 1.59] |
| Sweden | 2.21[1.87, 2.61] | 1.83[1.62, 2.06] | 1.18[0.82, 1.7] |
| Netherlands | 2.93[2.44, 3.53] | 1.88[1.63, 2.17] | 1.36[0.95, 1.97] |
| Spain | 2.81[2.36, 3.35] | 1.98[1.77, 2.21] | 1.41[1.22, 1.63] |
| Italy | 2.78[2.33, 3.31] | 2.2[1.97, 2.46] | 1.3[1.11, 1.52] |
| France | 2.32[2.02, 2.67] | 1.73[1.58, 1.89] | 1.39[1.15, 1.67] |
| Denmark | 2.22[1.85, 2.65] | 2.08[1.83, 2.38] | 1.51[1.01, 2.27] |
| Greece | 3.04[2.28, 4.06] | 2.06[1.72, 2.48] | 1.32[1.02, 1.71] |
| Switzerland | 2.39[2.06, 2.77] | 1.63[1.44, 1.85] | 0.71[0.4, 1.25] |
| Belgium | 1.78[1.59, 2] | 1.63[1.49, 1.77] | 1.06[0.84, 1.33] |
| Israel | 2.97[2.08, 4.25] | 1.99[1.62, 2.43] | 1.04[0.7, 1.53] |
| Czech Republic | 2.26[1.97, 2.59] | 1.76[1.62, 1.92] | 1.4[1.14, 1.73] |
| Poland | 2.58[1.99, 3.35] | 1.45[1.26, 1.67] | 0.93[0.71, 1.2] |
| Luxembourg | 2.31[1.78, 3] | 1.65[1.38, 1.98] | 1.17[0.78, 1.77] |
| Hungary | 2.63[2, 3.47] | 1.85[1.57, 2.19] | 0.89[0.63, 1.25] |
| Portugal | 2.92[1.96, 4.35] | 1.73[1.29, 2.31] | 1.11[0.74, 1.67] |
| Slovenia | 2.1[1.81, 2.44] | 1.57[1.4, 1.77] | 0.85[0.62, 1.17] |
| Estonia | 2.43[2.1, 2.81] | 1.72[1.58, 1.87] | 1.23[1, 1.51] |
| Croatia | 2.13[1.62, 2.79] | 1.78[1.46, 2.17] | 1.09[0.72, 1.65] |
| Lithuania | 2.9[1.92, 4.37] | 2.25[1.76, 2.87] | 1.23[0.72, 2.1] |
| Bulgaria | 2.86[1.46, 5.6] | 2.29[1.57, 3.34] | 0.81[0.39, 1.7] |
| Cyprus | 2.07[0.6, 7.1] | 1.32[0.6, 2.91] | 0.6[0.2, 1.78] |
| Finland | 1.74[1.04, 2.93] | 1.36[0.96, 1.93] | 0.93[0.23, 3.75] |
| Latvia | 1.6[0.88, 2.91] | 1.68[1.13, 2.52] | 0.93[0.43, 2.04] |
| Romania | 2.87[1.92, 4.3] | 2.16[1.64, 2.85] | 1.54[1.06, 2.23] |
| Slovakia | 4.52[2.33, 8.75] | 3.68[2.39, 5.67] | 1.14[0.49, 2.67] |
| **Overall effects** | **2.41[2.26, 2.55]** | **1.77[1.68, 1.85]** | **1.14[1.06, 1.21]** |

Table S. Variance components and ICC from mixed models.

| Model | Outcome | Variance | ICC |
| --- | --- | --- | --- |
| Country-level random intercept model | Outpatient | 0.523 | 0.137 |
|  | Inpatient | 0.142 | 0.041 |
|  | Number of outpatient visits | 0.066 | 0.071 |
|  | Number of inpatient admissions | 0.112 | 0.127 |
| Country-level random slope model | Outpatient | 0.167 | 0.119 |
|  | Inpatient | 0.710 | 0.056 |
|  | Number of outpatient visits | 0.215 | 0.177 |
|  | Number of inpatient admissions | 0.175 | 0.149 |

Table T. Changes in between-country heterogeneity (I²) (outpatient).

| Multimorbidity | Macro indicator | I²_baseline | I²_post | Δ I² |
| --- | --- | --- | --- | --- |
| Phy | Gini index | 89.88 | 88.18 | -1.70 |
|  | GDP per capita (USD) | 89.88 | 89.76 | -0.12 |
|  | Health expenditure per capita (USD) | 89.88 | 89.71 | -0.17 |
|  | Universal health coverage index | 89.88 | 89.98 | 0.10 |
| Psy | GDP per capita (USD) | 67.49 | 55.68 | -11.81 |
|  | Universal health coverage index | 67.49 | 30.84 | -36.65 |
|  | Gini index | 67.49 | 63.59 | -3.90 |
|  | Health expenditure per capita (USD) | 67.49 | 53.02 | -14.47 |
| Cog | Gini index | 58.42 | 58.58 | 0.16 |
|  | GDP per capita (USD) | 58.42 | 60.14 | 1.72 |
|  | Health expenditure per capita (USD) | 58.42 | 60.58 | 2.16 |
|  | Universal health coverage index | 58.42 | 59.64 | 1.22 |
| Phy + Psy | GDP per capita (USD) | 88.66 | 85.95 | -2.71 |
|  | Health expenditure per capita (USD) | 88.66 | 86.57 | -2.09 |
|  | Universal health coverage index | 88.66 | 77.51 | -11.15 |
|  | Gini index | 88.66 | 84.10 | -4.56 |
| Phy + Cog | Universal health coverage index | 52.09 | 11.59 | -40.50 |
|  | Health expenditure per capita (USD) | 52.09 | 49.79 | -2.30 |
|  | GDP per capita (USD) | 52.09 | 50.28 | -1.81 |
|  | Gini index | 52.09 | 47.25 | -4.84 |
| Psy + Cog | Health expenditure per capita (USD) | 75.85 | 66.85 | -9.00 |
|  | Universal health coverage index | 75.85 | 59.11 | -16.74 |
|  | GDP per capita (USD) | 75.85 | 68.85 | -7.00 |
|  | Gini index | 75.85 | 73.19 | -2.66 |
| Phy + Psy + Cog | Gini index | 99.47 | 90.24 | -9.23 |
|  | GDP per capita (USD) | 99.47 | 89.06 | -10.41 |
|  | Universal health coverage index | 99.47 | 91.14 | -8.33 |
|  | Health expenditure per capita (USD) | 99.47 | 89.43 | -10.04 |

Table U. Changes in between-country heterogeneity (I²) (inpatient).

| Multimorbidity | Macro indicator | I²_baseline | I²_post | Δ I² |
| --- | --- | --- | --- | --- |
| Phy | Health expenditure per capita (USD) | 45.84 | 11.80 | -34.04 |
|  | GDP per capita (USD) | 45.84 | 12.94 | -32.90 |
|  | Gini index | 45.84 | 12.37 | -33.47 |
|  | Universal health coverage index | 45.84 | 10.75 | -35.09 |
| Psy | Health expenditure per capita (USD) | 49.28 | 51.59 | 2.31 |
|  | Universal health coverage index | 49.28 | 43.53 | -5.75 |
|  | Gini index | 49.28 | 50.38 | 1.10 |
|  | GDP per capita (USD) | 49.28 | 51.91 | 2.63 |
| Cog | GDP per capita (USD) | 0.00 | 0.00 | 0.00 |
|  | Gini index | 0.00 | 1.24 | 1.24 |
|  | Universal health coverage index | - | - | - |
|  | Health expenditure per capita (USD) | - | - | - |
| Phy + Psy | Universal health coverage index | 58.72 | 50.20 | -8.52 |
|  | Gini index | 58.72 | 46.02 | -12.70 |
|  | Health expenditure per capita (USD) | 58.72 | 51.97 | -6.75 |
|  | GDP per capita (USD) | 58.72 | 51.91 | -6.81 |
| Phy + Cog | Gini index | 16.08 | 0.00 | -16.08 |
|  | Universal health coverage index | 16.08 | 13.23 | -2.85 |
|  | GDP per capita (USD) | 16.08 | 6.92 | -9.16 |
|  | Health expenditure per capita (USD) | 16.08 | 6.09 | -9.99 |
| Psy + Cog | Health expenditure per capita (USD) | 36.69 | 38.00 | 1.31 |
|  | Universal health coverage index | 36.69 | 32.12 | -4.57 |
|  | GDP per capita (USD) | 36.69 | 35.63 | -1.06 |
|  | Gini index | 36.69 | 0.00 | -36.69 |
| Phy + Psy + Cog | GDP per capita (USD) | 99.84 | 86.97 | -12.87 |
|  | Gini index | 99.84 | 85.99 | -13.85 |
|  | Universal health coverage index | 99.84 | 85.91 | -13.93 |
|  | Health expenditure per capita (USD) | 99.84 | 87.62 | -12.22 |

Table V. Changes in between-country heterogeneity (I²) (number of outpatient visits).

| Multimorbidity | Macro indicator | I²_baseline | I²_post | Δ I² |
| --- | --- | --- | --- | --- |
| Phy | Health expenditure per capita (USD) | 97.60 | 97.16 | -0.44 |
|  | Universal health coverage index | 97.60 | 97.04 | -0.56 |
|  | Gini index | 97.60 | 96.12 | -1.48 |
|  | GDP per capita (USD) | 97.60 | 97.12 | -0.48 |
| Psy | Health expenditure per capita (USD) | 80.17 | 77.45 | -2.72 |
|  | Universal health coverage index | 80.17 | 67.79 | -12.38 |
|  | GDP per capita (USD) | 80.17 | 78.74 | -1.43 |
|  | Gini index | 80.17 | 79.81 | -0.36 |
| Cog | Universal health coverage index | 82.84 | 84.35 | 1.51 |
|  | Gini index | 82.84 | 85.81 | 2.97 |
|  | Health expenditure per capita (USD) | 82.84 | 85.39 | 2.55 |
|  | GDP per capita (USD) | 82.84 | 85.43 | 2.59 |
| Phy + Psy | Health expenditure per capita (USD) | 96.29 | 95.57 | -0.72 |
|  | GDP per capita (USD) | 96.29 | 95.44 | -0.85 |
|  | Universal health coverage index | 96.29 | 95.76 | -0.53 |
|  | Gini index | 96.29 | 95.36 | -0.93 |
| Phy + Cog | Universal health coverage index | 75.98 | 55.11 | -20.87 |
|  | Gini index | 75.98 | 37.24 | -38.74 |
|  | GDP per capita (USD) | 75.98 | 73.87 | -2.11 |
|  | Health expenditure per capita (USD) | 75.98 | 73.94 | -2.04 |
| Psy + Cog | Gini index | 70.08 | 52.09 | -17.99 |
|  | Health expenditure per capita (USD) | 70.08 | 65.25 | -4.83 |
|  | GDP per capita (USD) | 70.08 | 65.79 | -4.29 |
|  | Universal health coverage index | 70.08 | 55.66 | -14.42 |
| Phy + Psy + Cog | Gini index | 99.83 | 95.36 | -4.47 |
|  | Health expenditure per capita (USD) | 99.83 | 95.66 | -4.17 |
|  | GDP per capita (USD) | 99.83 | 95.71 | -4.12 |
|  | Universal health coverage index | 99.83 | 96.16 | -3.67 |

Table W. Changes in between-country heterogeneity (I²) (number of inpatient admissions).

| Multimorbidity | Macro indicator | I²_baseline | I²_post | Δ I² |
| --- | --- | --- | --- | --- |
| Phy | GDP per capita (USD) | 50.62 | 23.47 | -27.15 |
|  | Health expenditure per capita (USD) | 50.62 | 22.40 | -28.22 |
|  | Universal health coverage index | 50.62 | 22.75 | -27.87 |
|  | Gini index | 50.62 | 24.78 | -25.84 |
| Psy | Gini index | 89.01 | 88.37 | -0.64 |
|  | Universal health coverage index | 89.01 | 89.20 | 0.19 |
|  | GDP per capita (USD) | 89.01 | 88.95 | -0.06 |
|  | Health expenditure per capita (USD) | 89.01 | 88.27 | -0.74 |
| Cog | Health expenditure per capita (USD) | - | - | - |
|  | Universal health coverage index | - | - | - |
|  | Gini index | - | - | - |
|  | GDP per capita (USD) | - | - | - |
| Phy + Psy | Gini index | 62.38 | 56.36 | -6.02 |
|  | Health expenditure per capita (USD) | 62.38 | 58.51 | -3.87 |
|  | GDP per capita (USD) | 62.38 | 58.59 | -3.79 |
|  | Universal health coverage index | 62.38 | 57.42 | -4.96 |
| Phy + Cog | Health expenditure per capita (USD) | 100.00 | 83.68 | -16.32 |
|  | Gini index | 100.00 | 81.89 | -18.11 |
|  | GDP per capita (USD) | 100.00 | 82.98 | -17.02 |
|  | Universal health coverage index | 100.00 | 82.18 | -17.82 |
| Psy + Cog | Health expenditure per capita (USD) | - | - | - |
|  | GDP per capita (USD) | - | - | - |
|  | Gini index | - | - | - |
|  | Universal health coverage index | - | - | - |
| Phy + Psy + Cog | Gini index | 99.79 | 87.82 | -11.97 |
|  | Universal health coverage index | 99.79 | 88.20 | -11.59 |
|  | Health expenditure per capita (USD) | 99.79 | 89.52 | -10.27 |
|  | GDP per capita (USD) | 99.79 | 89.04 | -10.75 |

Table X. Model fit comparison across models.

| Model | Outcome | Average AIC | Average BIC |
| --- | --- | --- | --- |
| Hurdle | Number of inpatient admissions | 15483.33 | 15791.61 |
| Hurdle | Number of outpatient visits | 60435.74 | 60748.18 |
| Negative binomial | Number of inpatient admissions | 12427.79 | 12599.92 |
| Negative binomial | Number of outpatient visits | 48382.98 | 48556.83 |
| Poisson | Number of inpatient admissions | 13974.46 | 14142.22 |
| Poisson | Number of outpatient visits | 57084.25 | 57251.78 |

Table Y. Predictors of attrition (logistic regression models).

| Multimorbidity (ref: None) | OR, 95%CI | P |
| --- | --- | --- |
| Phy | 0.211 [0.01, 4.519] | 0.32 |
| Psy | 1 |  |
| Cog | 1 |  |
| Phy + Psy | 1 |  |
| Phy + Cog | 1 |  |
| Psy + Cog | 1 |  |
| Phy + Psy + Cog | 1 |  |
| Household wealth (ref: Q1) |  |  |
| Q2 | 1 |  |
| Q3 | 0.264 [0.025, 2.747] | 0.265 |
| Q4 | 0.233 [0.021, 2.631] | 0.239 |
| Age | 1.016 [0.809, 1.276] | 0.892 |
| Gender (ref: Male) | 3.71 [0.411, 33.513] | 0.243 |
| Health insurance (ref: No) | 7.521 [0.721, 78.418] | 0.092 |

**Appendix Figures**

**
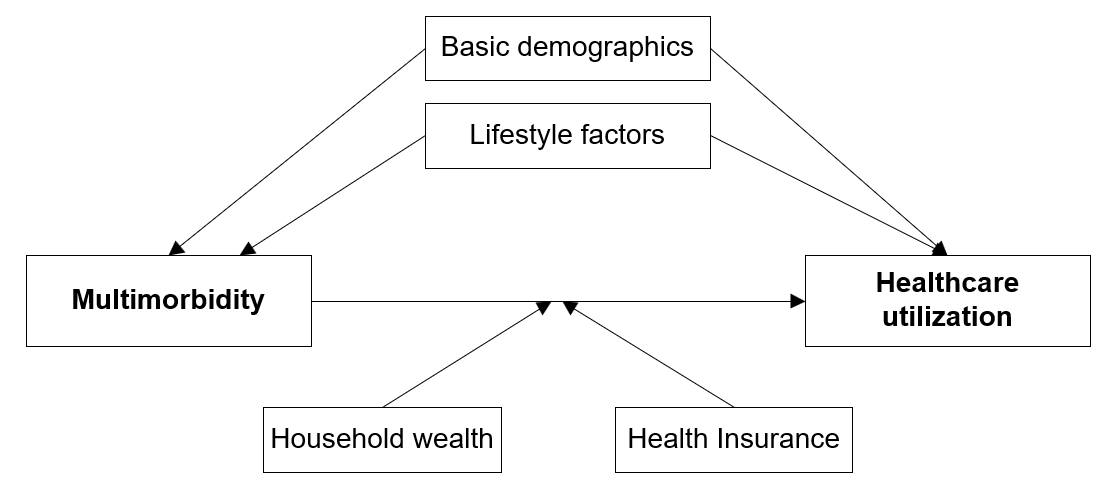
**

Figure A. Directed acyclic graph


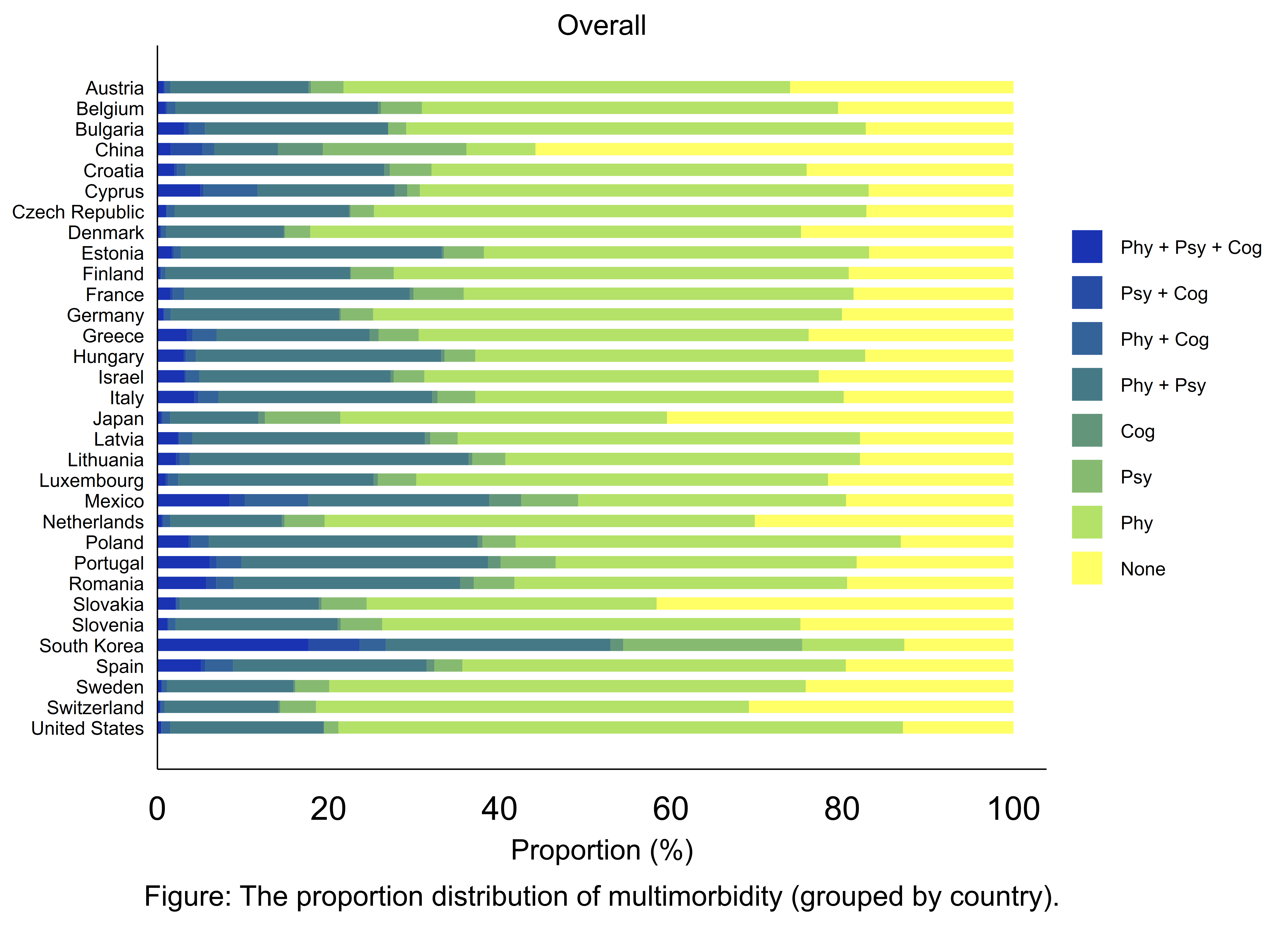


Figure B. Prevalence of physical, psychological, and cognitive multimorbidity by country.


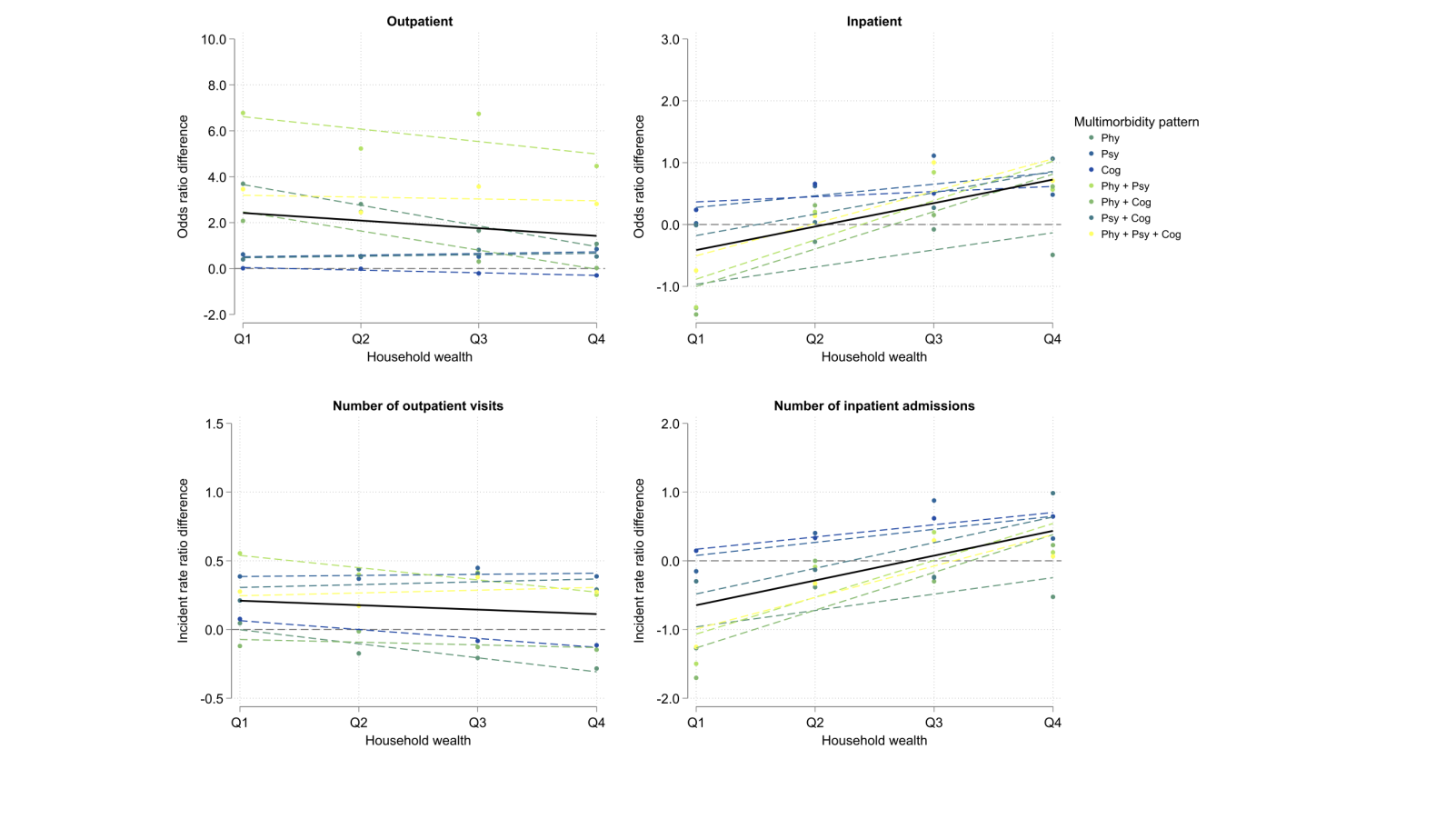


Figure C. The OR and IRR differences between individuals with and without health insurance (grouped by multimorbidity and SES).


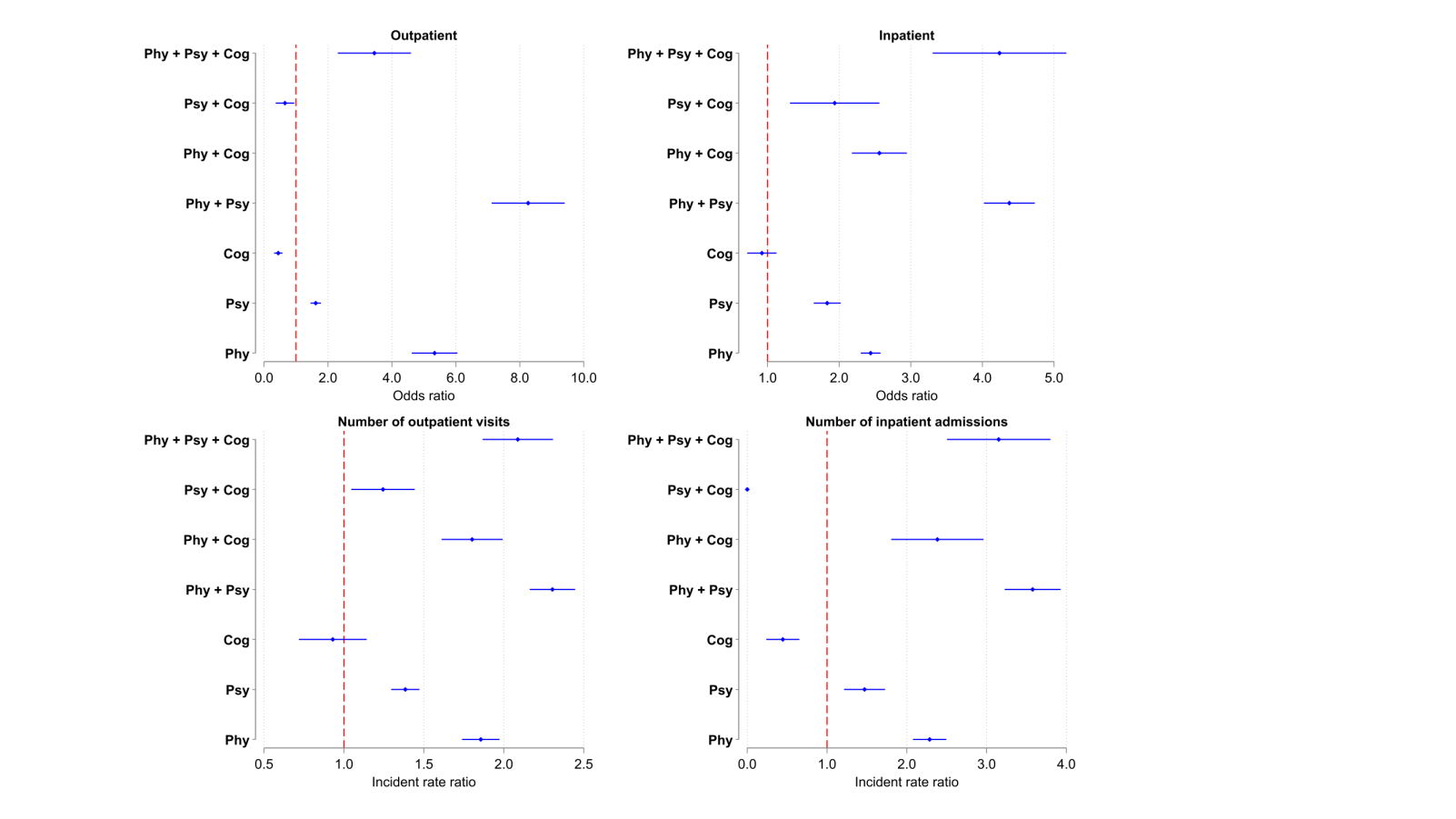


Figure D. The association between healthcare utilisation and multimorbidity (specific cohorts and poisson model analyses).

Note: Panel (A) is the result excluded HRS, CHARLS and KLoSA; Panel (B) is the result excluded HRS and KLoSA; Panel (C) and Panel (D) are results using random-effects poisson regression models.


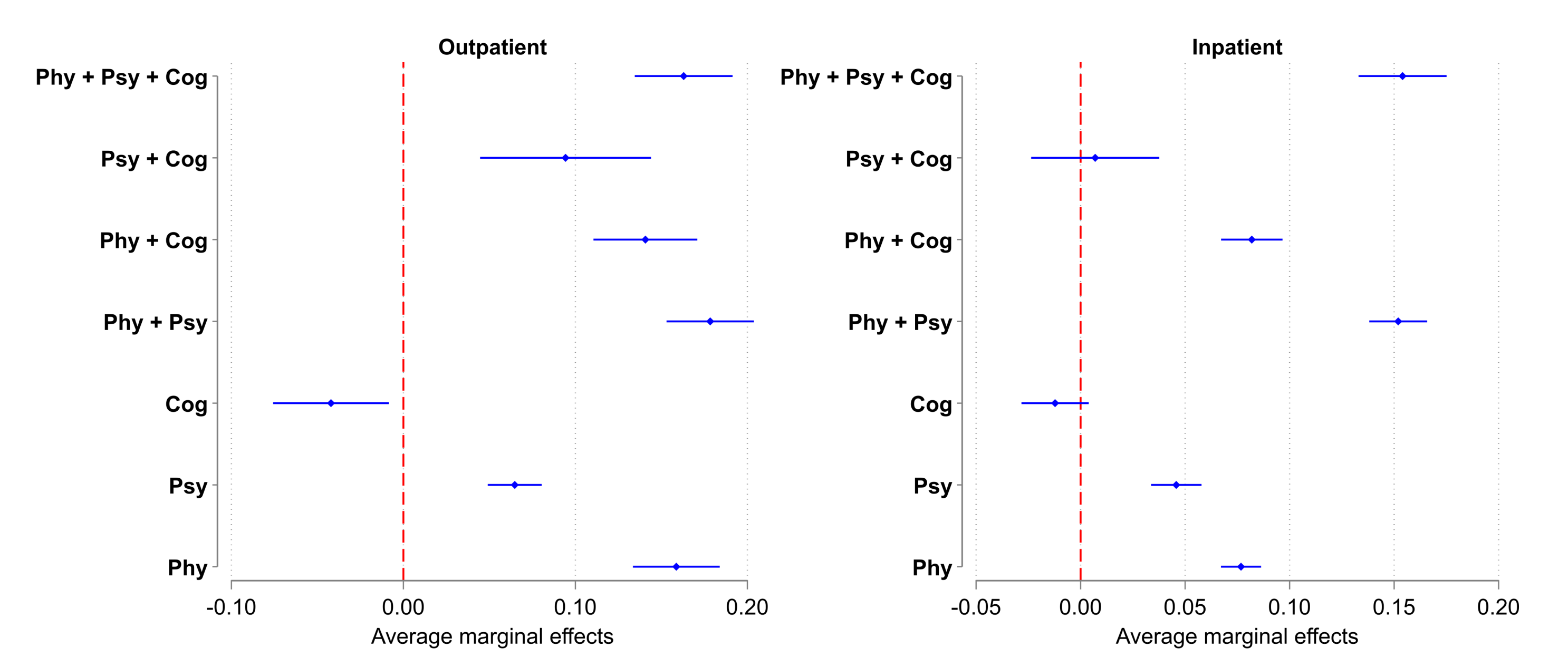


Figure E. The association between binary healthcare utilisation and multimorbidity (marginal effects derived from probit model).


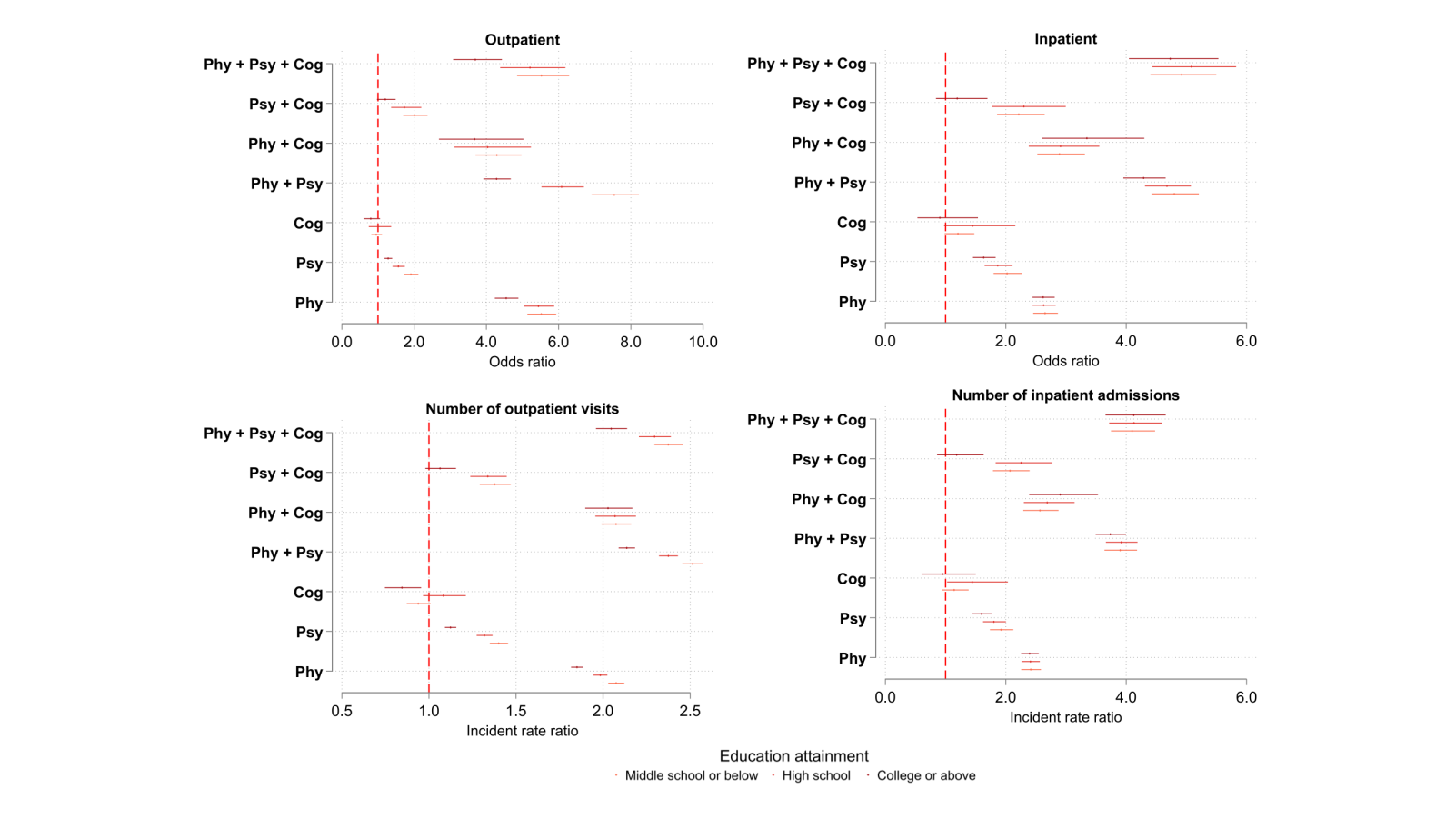


Figure F. The association between healthcare utilisation and multimorbidity (grouped by education attainment).


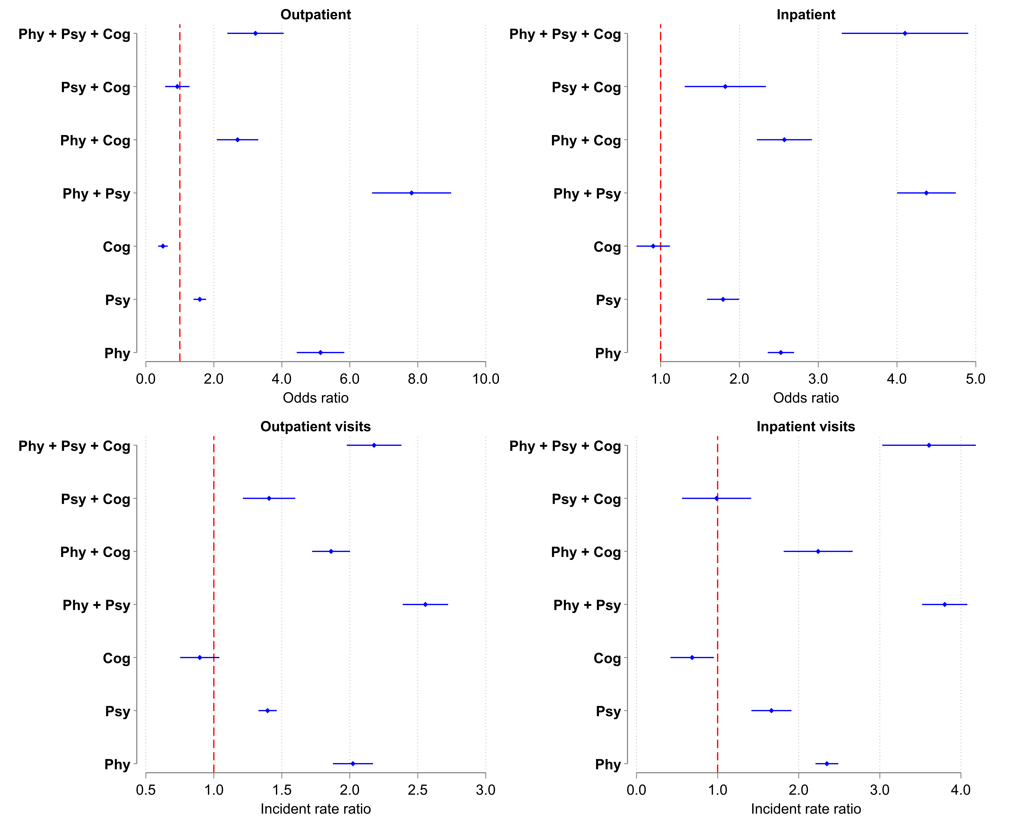


Figure G. The association between healthcare utilisation and multimorbidity (treating SES as an ordinal variable).


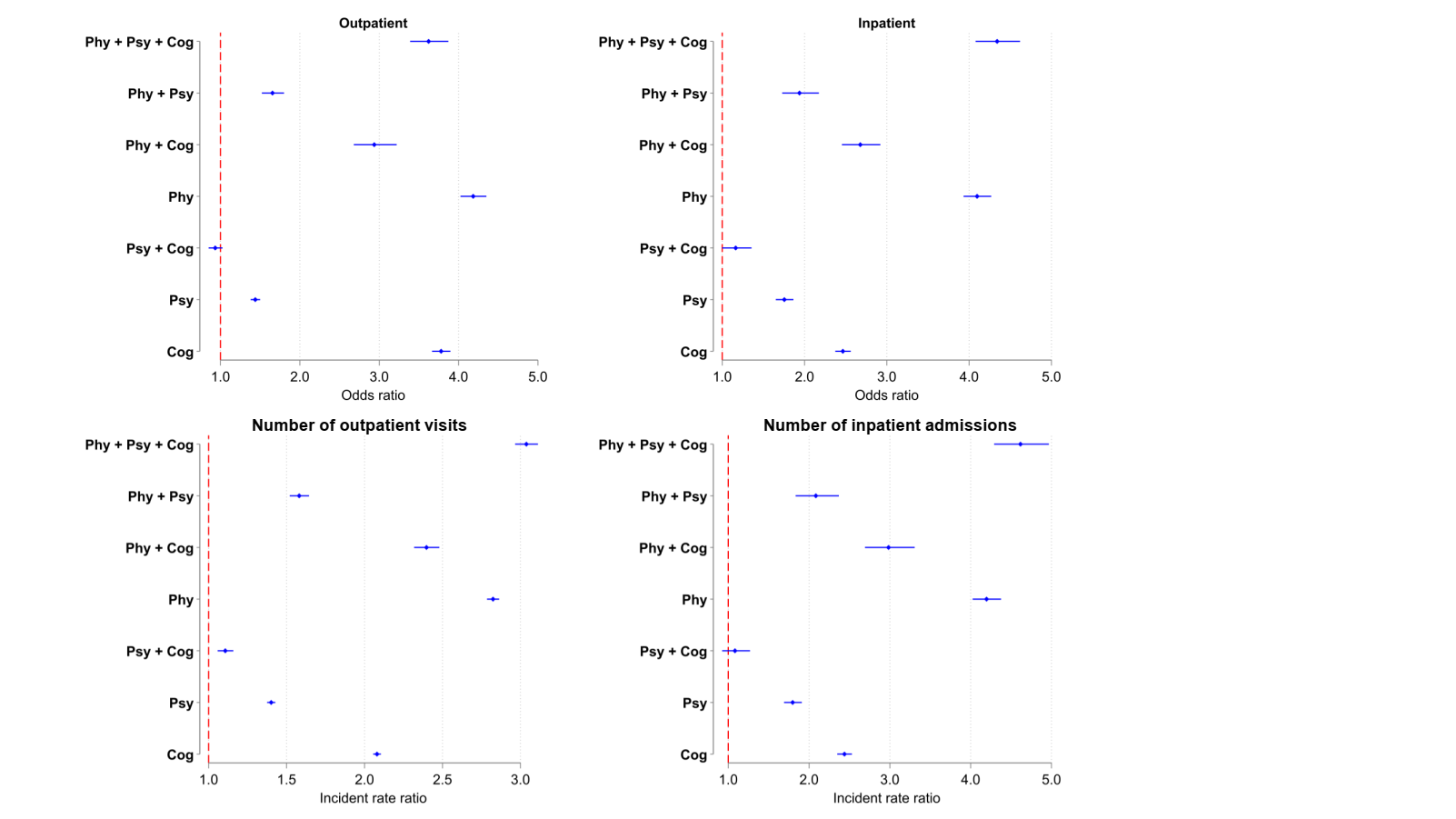


Figure H. The association between healthcare utilisation and multimorbidity (modelled by random intercepts model).


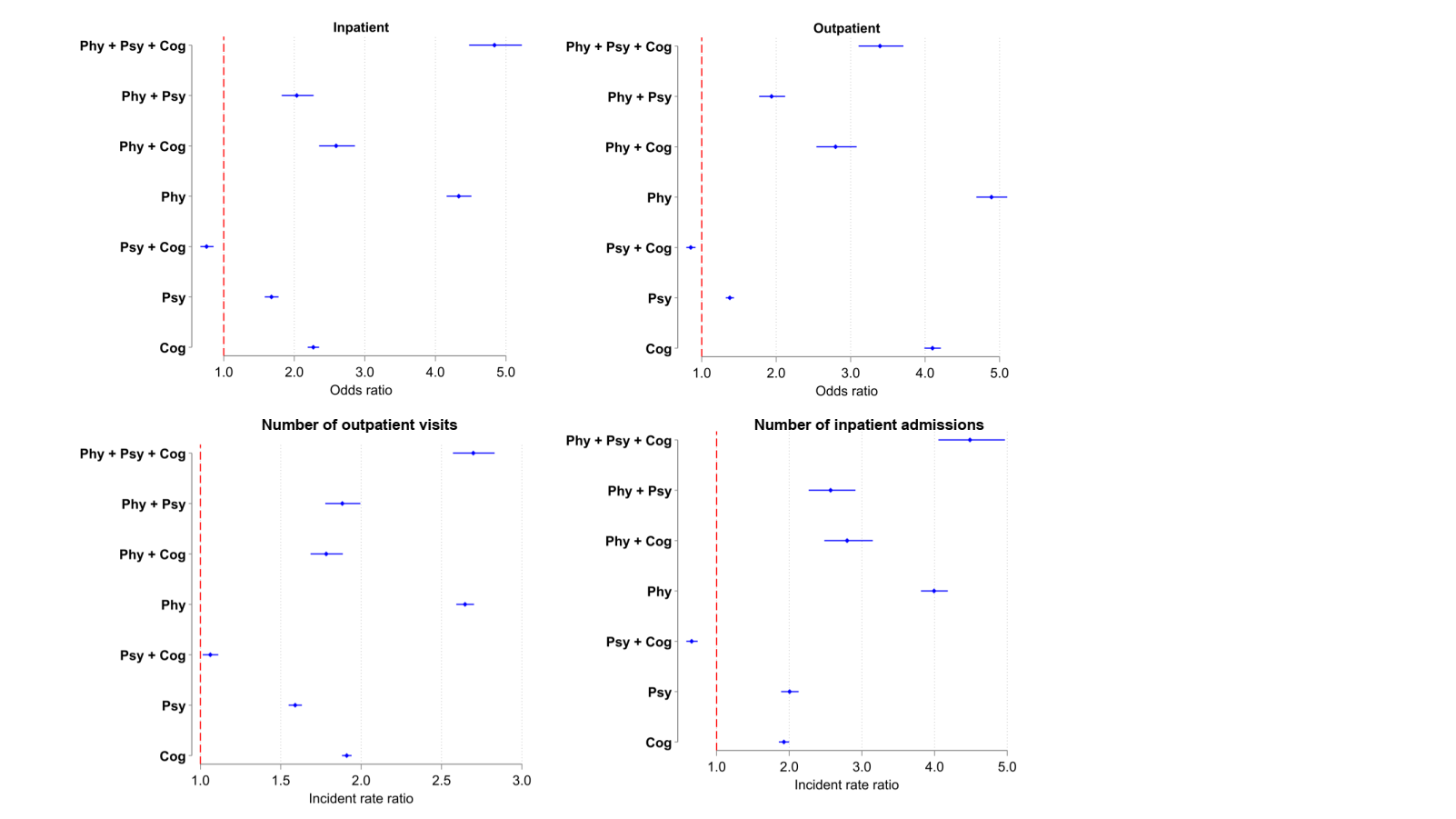


Figure I. The association between healthcare utilisation and multimorbidity (modelled by weighted multilevel model).


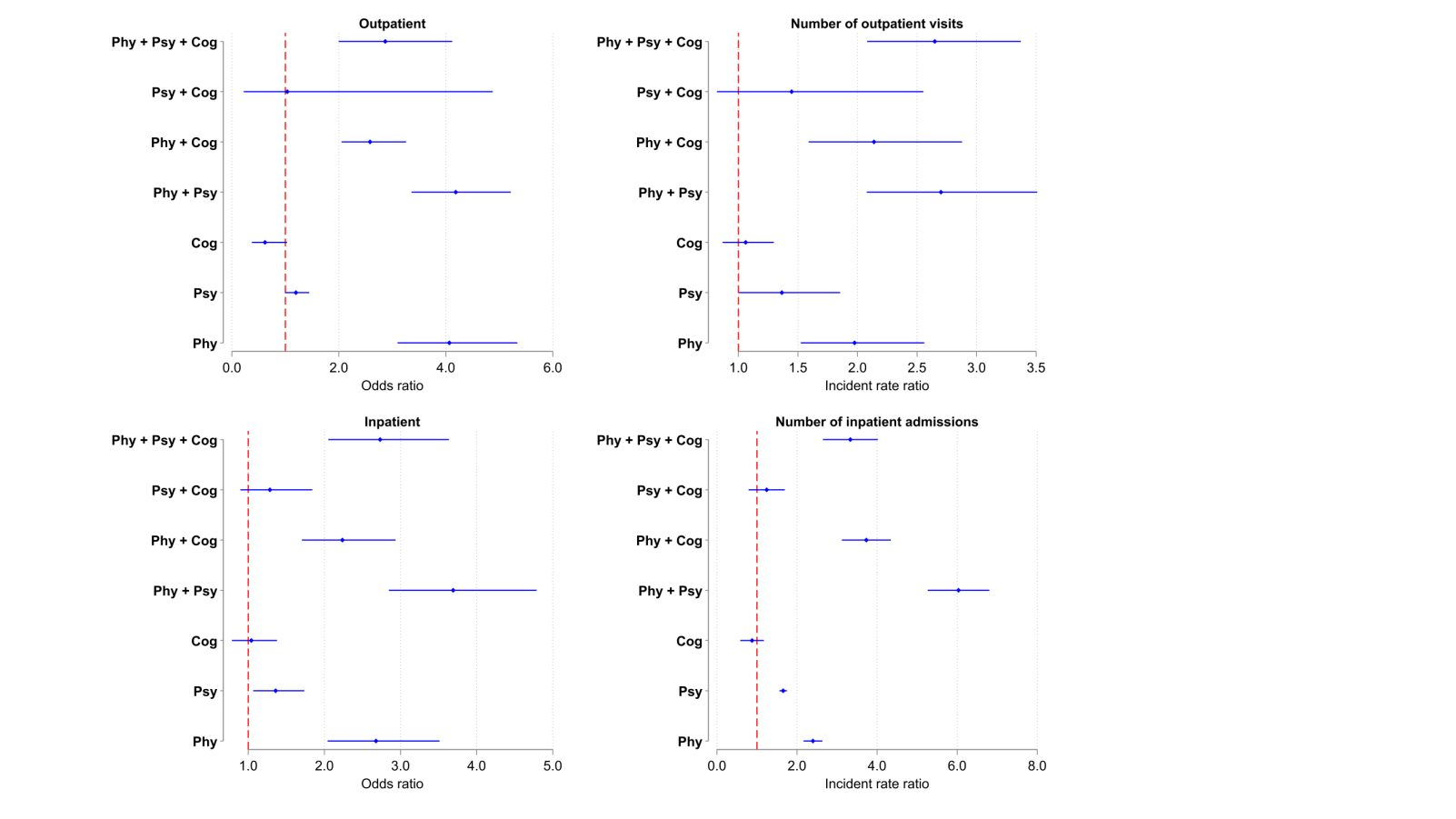


Figure J. The association between healthcare utilisation and multimorbidity (modelled by random slopes model).


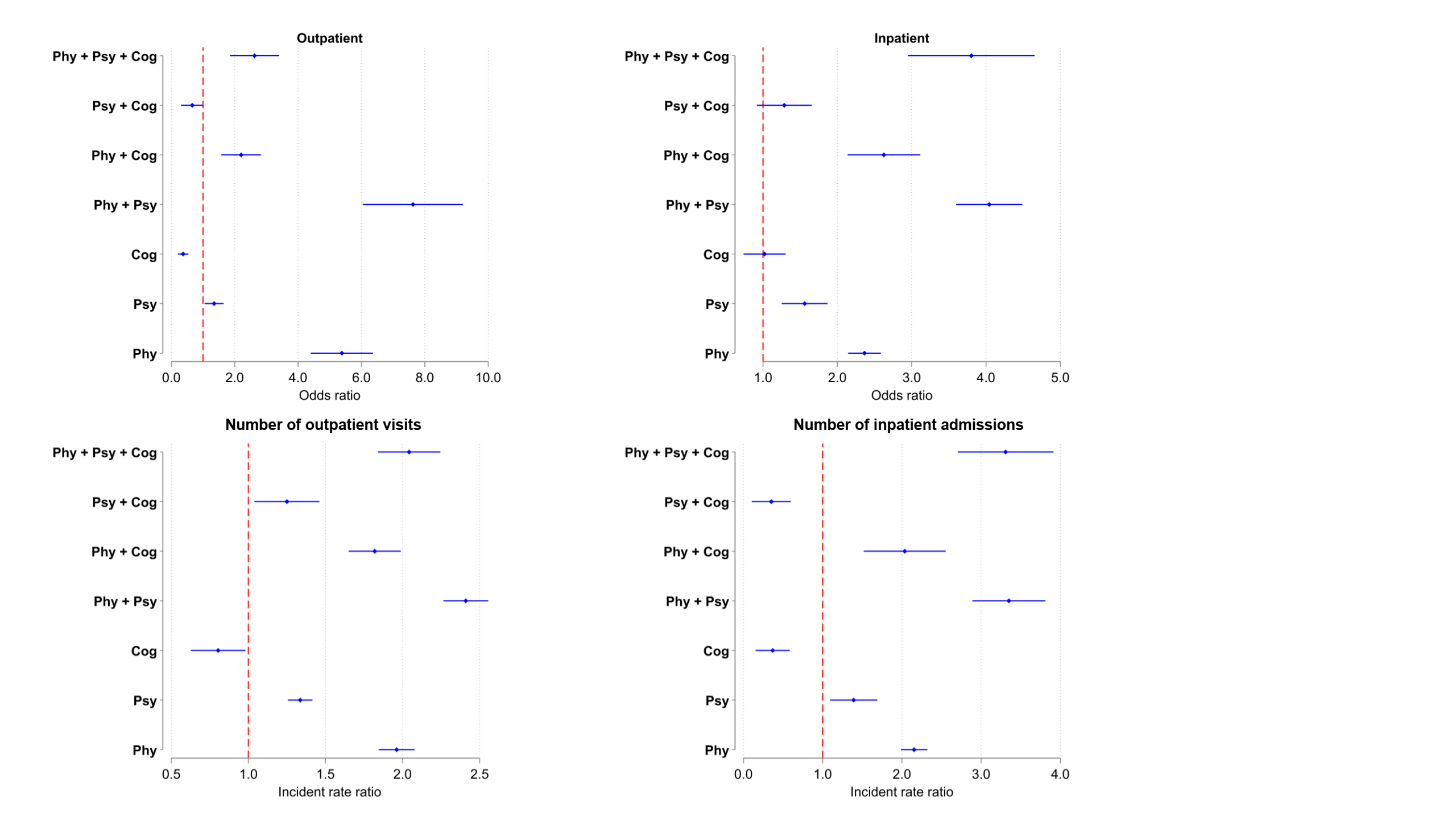


Figure K. The association between healthcare utilisation and multimorbidity (aged 65 and above).


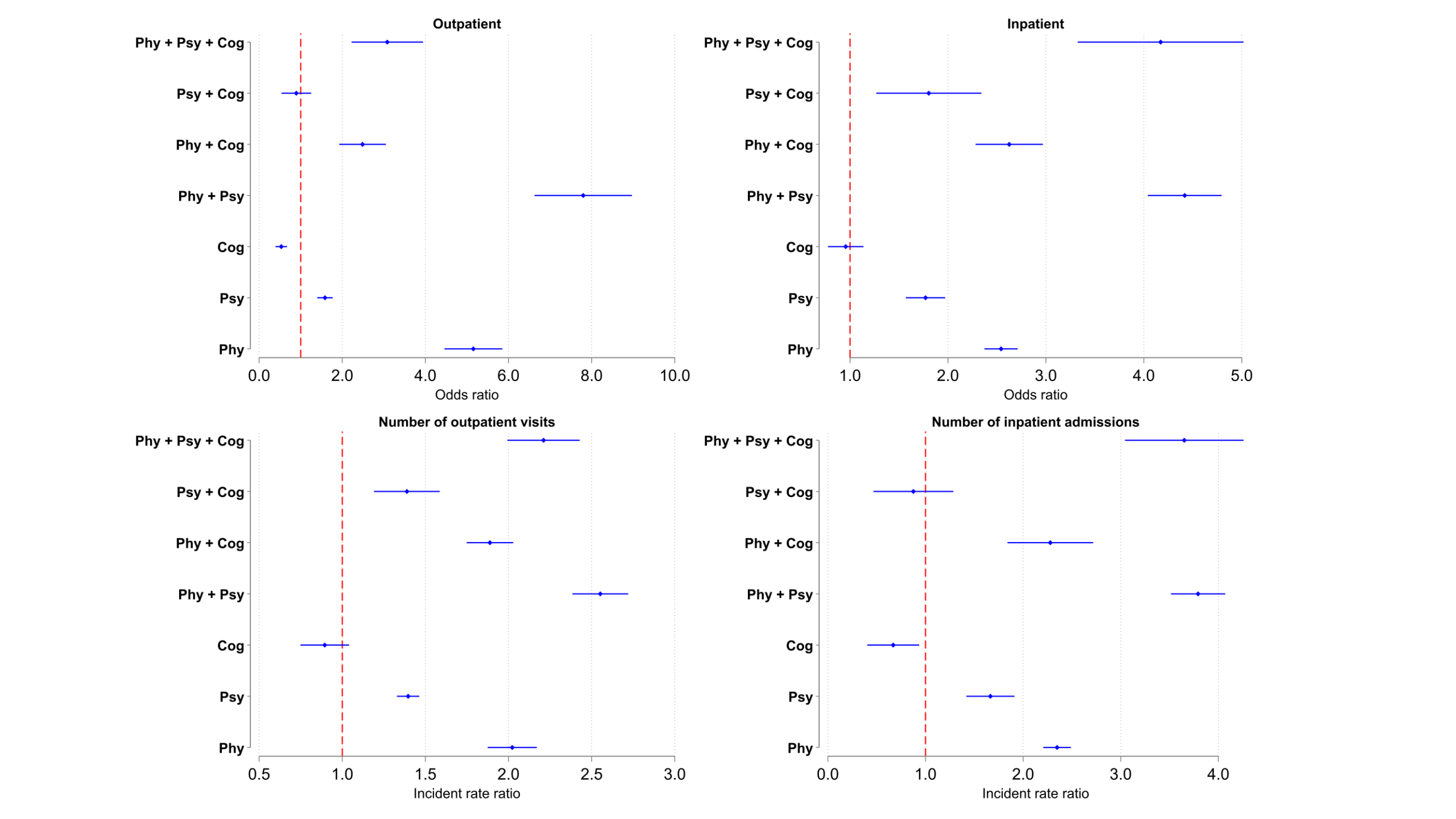


Figure L. The association between healthcare utilisation and multimorbidity (complete-case).


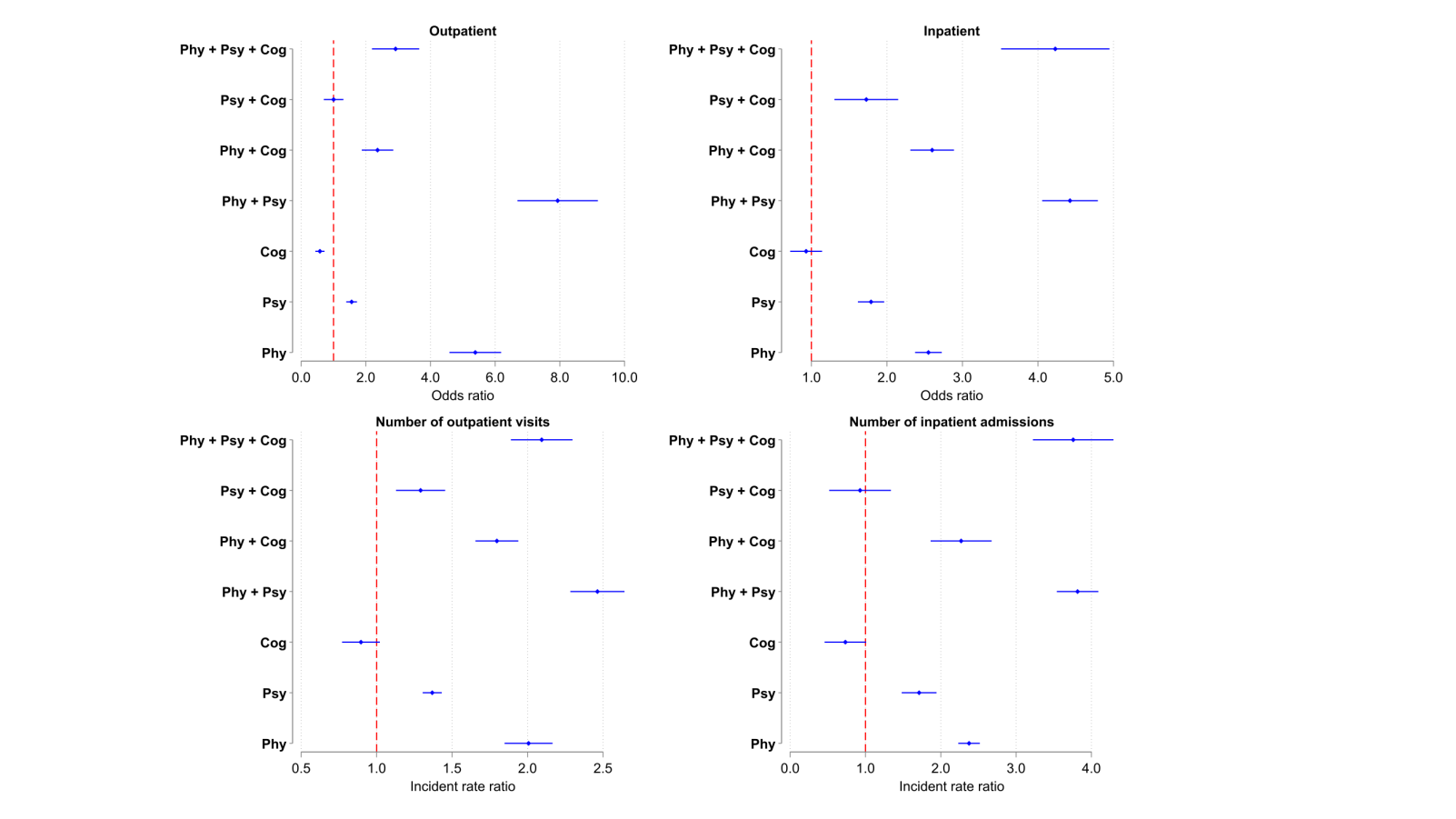


Figure M. The association between healthcare utilisation and multimorbidity (only control for baseline covariates).


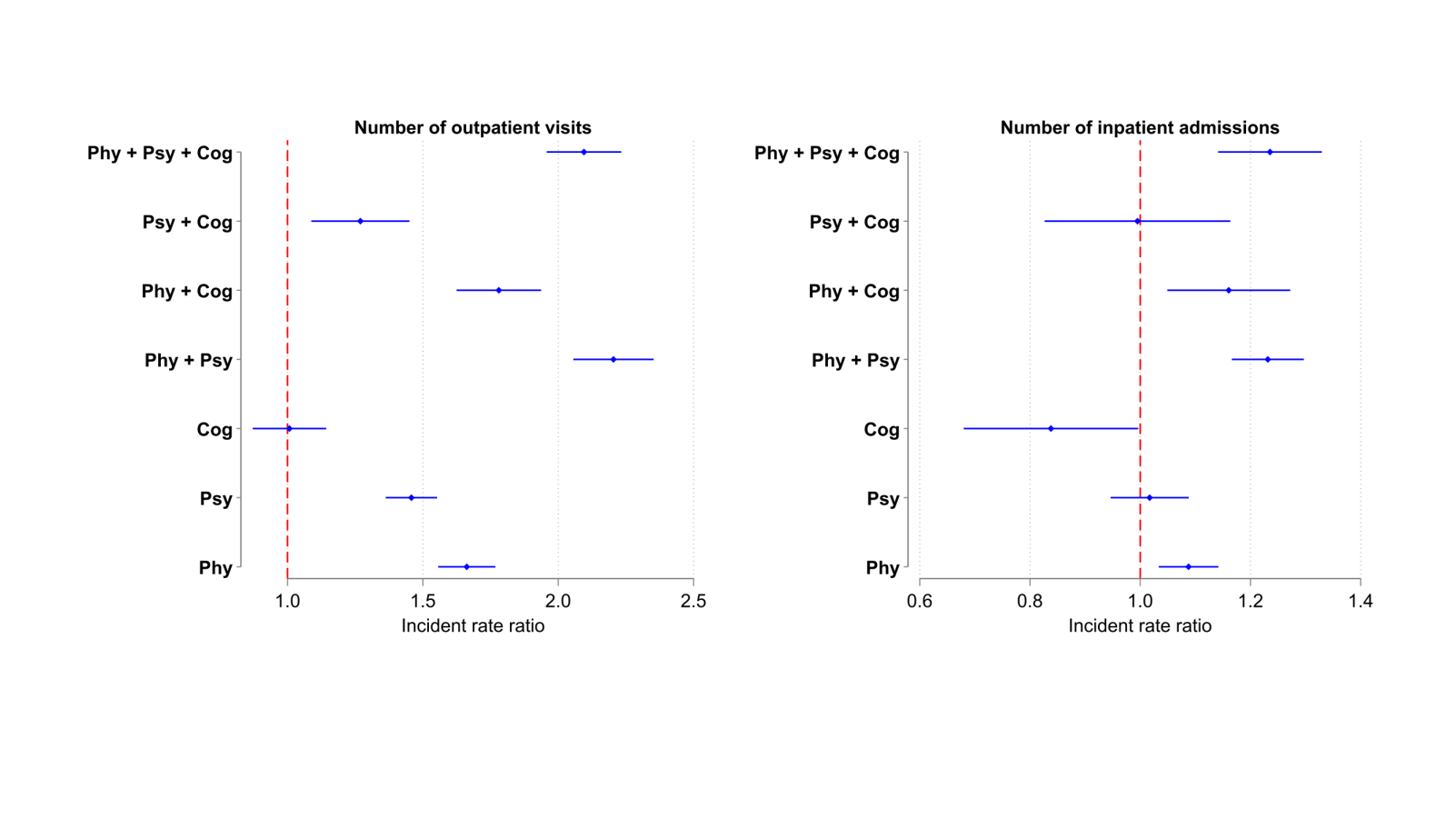


Figure N. The association between healthcare utilisation and multimorbidity (hurdle model).


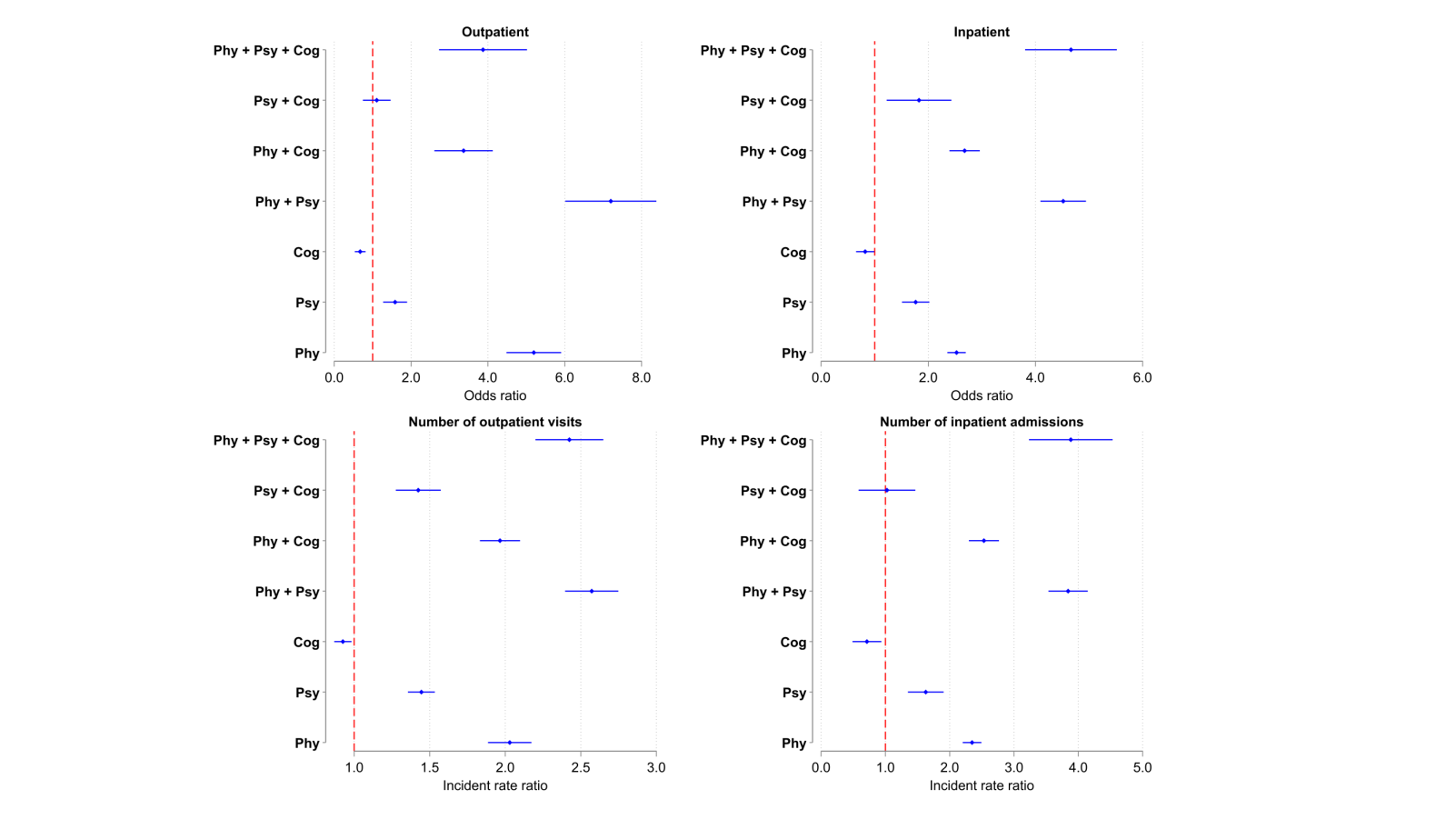


Figure O. The association between healthcare utilisation and multimorbidity (stricter screening cut-offs).

Note: In this definition, higher screening thresholds were applied to reduce potential false-positive classification. For SHARE and MHAS, cognitive disorder was defined as performance ≥1.5 SD below the cohort-specific mean (vs. 1 SD in the primary definition). For other cohorts, lower absolute score cut-offs were used. Psychological disorder required a higher symptom threshold, excluding borderline scores.


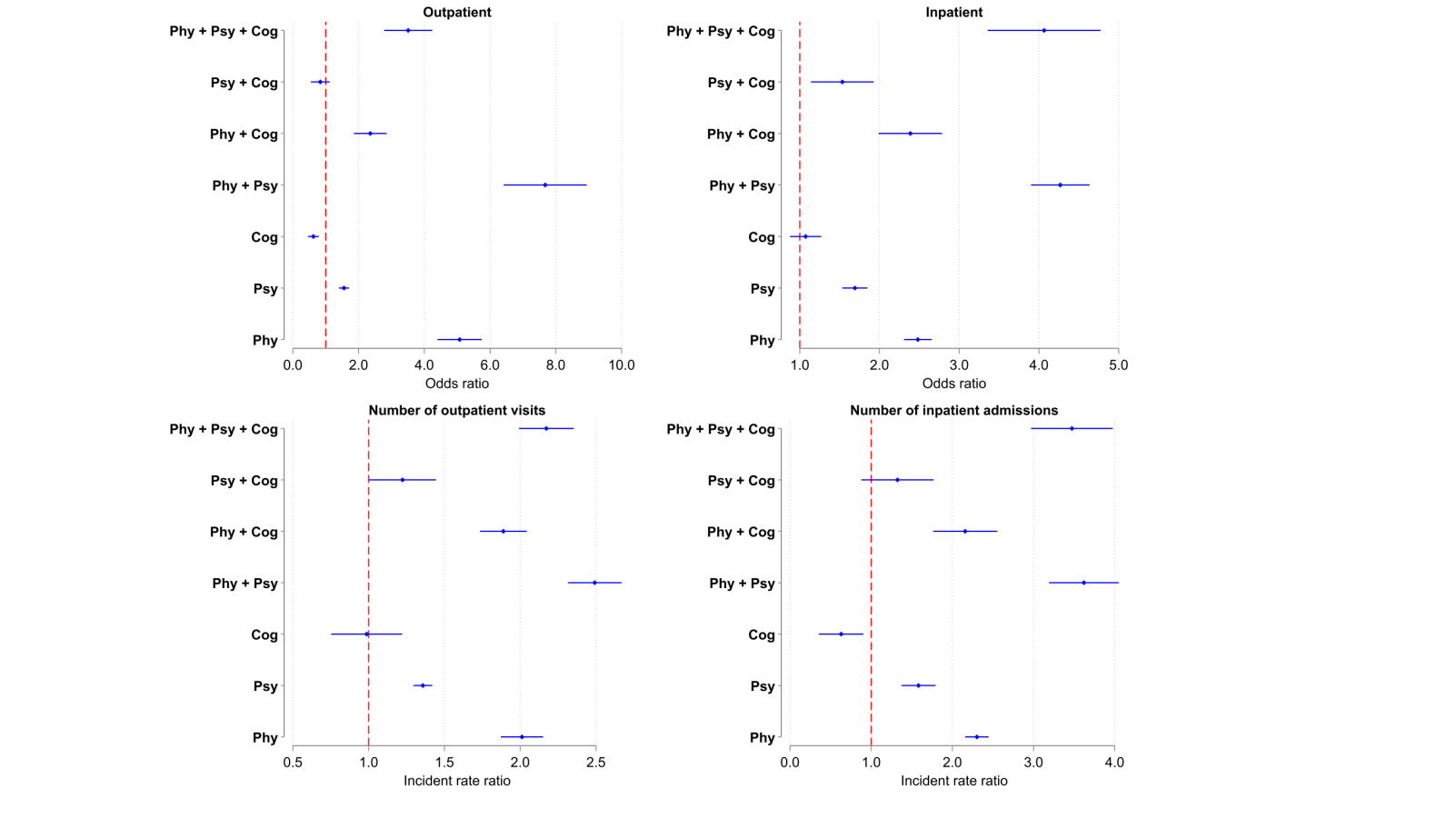


Figure P. The association between healthcare utilisation and multimorbidity (more lenient screening cut-offs).

Note: In this definition, lower screening thresholds were applied to include milder cases. For SHARE and MHAS, cognitive disorder was defined as performance ≥0.5 SD below the cohort-specific mean. For other cohorts, higher absolute score cut-offs were used. Psychological disorder was defined using a lower symptom threshold.

**Reference**

1. Kozlov, E., Dong, X., Kelley, A. S. & Ankuda, C. K. The Epidemiology of Depressive Symptoms in the Last Year of Life. *Journal of the American Geriatrics Society* **68**, 321–328 (2020).

2. Conde-Sala, J. L., Garre-Olmo, J., Calvó-Perxas, L., Turró-Garriga, O. & Vilalta-Franch, J. Course of depressive symptoms and associated factors in people aged 65+ in Europe: A two-year follow-up. *Journal of Affective Disorders* **245**, 440–450 (2019).

3. Han, C. H., Chung, J. H. & Lee, S. Depression, chronic obstructive pulmonary disease, and healthcare utilisation: Results from the Korean Longitudinal Study of Aging (KLoSA). *The Clinical Respiratory Journal* **15**, 937–943 (2021).

4. Yang, X. *et al.* Prospective associations between depressive symptoms and cognitive functions in middle-aged and elderly Chinese adults. *Journal of Affective Disorders* **263**, 692–697 (2020).

5. Zhou, L., Ma, X. & Wang, W. Relationship between Cognitive Performance and Depressive Symptoms in Chinese Older Adults: The China Health and Retirement Longitudinal Study (CHARLS). *Journal of Affective Disorders* **281**, 454–458 (2021).

6. Castellanos-Perilla, N. *et al.* Factors associated with functional loss among community-dwelling Mexican older adults. *Biomédica* **40**, 546–556 (2020).

7. Richardson, R. A., Keyes, K. M., Medina, J. T. & Calvo, E. Sociodemographic inequalities in depression among older adults: cross-sectional evidence from 18 countries. *The Lancet Psychiatry* **7**, 673–681 (2020).

8. Langa, K. M. *et al.* Trends in the prevalence and mortality of cognitive impairment in the United States: Is there evidence of a compression of cognitive morbidity? *Alzheimer’s & Dementia* **4**, 134–144 (2008).

9. Sutin, A. R., Luchetti, M., Stephan, Y. & Terracciano, A. Meaning in life and risk of cognitive impairment: A 9-Year prospective study in 14 countries. *Archives of Gerontology and Geriatrics* **88**, 104033 (2020).

10. Kim, J.-H. & Chon, D. Association between Cognitive Impairment, Vascular Disease and All-Cause Mortality. *The Journal of nutrition, health and aging* **22**, 790–795 (2018).

11. Zhou, S., Song, S., Jin, Y. & Zheng, Z.-J. Prospective association between social engagement and cognitive impairment among middle-aged and older adults: evidence from the China Health and Retirement Longitudinal Study. *BMJ Open* **10**, e040936 (2020).

12. Oba, H., Kadoya, Y., Matsuoka, T. & Narumoto, J. Cognitive decline reduces household spending among older people. *Psychogeriatrics* **20**, 28–34 (2020).
