# Supplementary material for: Multimorbidity, health service use, and health insurance by socioeconomic groups in 31 countries: A multi-cohort study
Source: PLoS Med. 2026 May 5;23(5):e1005087. doi: 10.1371/journal.pmed.1005087 (PMC13160435; doi:10.1371/journal.pmed.1005087)
Supplement: Attachment — Submitted filename: Response to Reviewers.docx [file pmed.1005087.s004.docx]

Dear Reviewers,

Thank you so much for taking your time and effort to review our manuscript entitled *“Multimorbidity, health service use, and health insurance by socioeconomic groups in 31 countries: a multi-cohort study”*. We appreciate all your valuable comments and suggestions, which are very helpful in improving the quality of our paper. All the comments raised by reviewers have been addressed properly in this revised manuscript.

We have undertaken substantial revisions to address all concerns raised by the reviewers. Please find our itemized responses below and revised manuscript.

------------------------------------
***Reviewer #1***

Q1: The manuscript employs random-effects logistic regression for binary utilisation outcomes and random-effects negative binomial models for counts. It is unclear whether random effects were specified at the individual, wave, or country level, or simultaneously at multiple levels. Given the multinational design, further justification is needed for using random rather than fixed effects for countries, particularly since unobserved country-specific heterogeneity could bias results.

**Response:** Thanks for your point. In our analyses, the random effects were specified at the individual level and survey-wave level. In the pooled multi-country models, we additionally included country fixed effects. We have clarified this in the ***Statistical Analysis*** section.

Q2: Country-specific estimates are aggregated via random-effects meta-analysis after already estimating random-effects models. The rationale for this two-layer randomisation should be explained.

**Response:** Thank you for this comment. The random effects in the country-specific models address within-country individual-level repeated measures, whereas the random-effects meta-analysis accounts for between-country heterogeneity when pooling estimates. These two components therefore capture variation at different hierarchical levels. In addition, to further assess the robustness of our findings, we re-estimated the pooled sample using a multilevel model, and the results remained consistent (see ***Appendix Figure 7***).

Q3: Appendix Table 10 shows extremely high heterogeneity (I² often >90%). This raises doubts about whether pooled effect sizes are meaningful. Alternative approaches, such as meta-regression to account for country-level factors, should be considered or at least discussed.

**Response:** Thank you for raising this. As our study does not focus on explaining country-level determinants, we did not perform a meta-regression. However, to address this concern, we conducted an additional robustness analysis using a multilevel model in the pooled dataset, specifying country as a random intercept to explicitly account for between-country heterogeneity. The results remained consistent with the main findings. We have added this explanation to the ***Sensitivity analyses*** (see ***Appendix Figure 7***).

Q4: There are substantial differences in outcome measurement (e.g., outpatient care measured over 1 month in CHARLS, 12 months in SHARE, 24 months in KLoSA). The harmonization strategy is not clearly described, and residual comparability issues may bias results.

**Response:** Thanks for your point. To address this, we conducted sensitivity analyses using only countries with harmonised measurement windows. Specifically, for outpatient care, we excluded HRS, CHARLS, and KLoSA and re-estimated the models. For inpatient care, we excluded HRS and KLoSA. As shown in ***Appendix Figure 4*** (***Sensitivity Analyses***), the results remained consistent with the main findings.

Q5: Similarly, definitions of psychological and cognitive disorders vary across cohorts, with different screening tools and cut-offs. This may introduce misclassification and heterogeneity. These limitations should be acknowledged more explicitly.

**Response:** Thank you for the comment. We have now made this limitation more explicit in the ***Limitations*** section. In addition, ***Appendix Table 1*** already documents the specific measurement instruments and cut-offs used in each cohort.

Q6: SES is proxied by within-country, within-wave household wealth quartiles. While useful for relative comparisons, this obscures absolute differences across countries. The interpretation of gradient effects should be restricted to within-country SES variation, not cross-national comparisons.

**Response:** Thank you for this helpful comment. We have further clarified this point in the section “***The association between multimorbidity and healthcare utilization***”.

Q7: Health insurance coverage is treated as a binary variable, but definitions differ substantially across datasets, and information is missing entirely for JSTAR. The handling of this missingness and the impact on pooled analyses need to be clarified.

**Response:** Thank you for this comment. In the pooled analyses, we excluded JSTAR from all models involving health insurance because insurance information is not available in this cohort. We also agree that dichotomizing health insurance status may mask important cross-national differences. However, despite variation in health insurance schemes across countries, classifying individuals as insured versus uninsured captures the fundamental distinction between having some degree of financial protection and having none—a contrast that remains meaningful across diverse health system contexts. More detailed harmonization of insurance types was not feasible given the lack of comparable information across cohorts. This limitation has now been made more explicit in the ***Limitations*** section.

Q8: The primary analysis relies on complete cases, while multiple imputation was only used as a sensitivity check. Given the scale of missing data across cohorts, it would be preferable to use imputation in the main analyses. At minimum, the proportion of excluded participants should be reported.

**Response:** Thanks for your comment. ***Appendix Table 5*** reports the proportion of missing data for all variables. We kept the complete-case analysis as the primary approach because of differences in variable availability across cohorts, which make large-scale imputation less reliable in the pooled dataset.

Q9: There is no mention of assessing model assumptions (e.g., overdispersion in negative binomial models, convergence, adequacy of random-effects structure). Reporting on diagnostics is necessary to assess the robustness of the findings.

**Response:** Thank you for this point. To assess the robustness of our model assumptions, we re-estimated the binary outcomes using random-effects probit models instead of logistic regression (***Appendix Figure 5***) and re-estimated the count outcomes using Poisson regression models in place of negative binomial models (***Appendix Figure 4***). The results were consistent with the main analyses.

Q10: Odds ratios may exaggerate associations when outcomes are common (e.g., >70% outpatient utilization in several countries). The use of risk ratios or marginal probabilities would be more interpretable.

**Response:** Thank you for this comment. We re-estimated all binary outcomes using random-effects probit models and reported the corresponding marginal probabilities (***Appendix Figure 5***). The results were consistent with the main analyses.

Q11: For rarer multimorbidity combinations (e.g., psychological-cognitive), sample sizes are very small in some countries, leading to unstable estimates with wide CIs. This should be highlighted more clearly.

**Response:** Thanks for your point. We agree that some multimorbidity combinations have small sample sizes in certain countries, which leads to wider confidence intervals. This is also one of the reasons we employed meta-analysis, which accounts for differences in sample size and uncertainty across countries.

Q12: Multiple subgroup and stratified analyses are conducted without correction for multiple testing. The risk of inflated type I error should be acknowledged.

**Response:** Thanks for your point. To address this, we formally tested the interaction terms (e.g., multimorbidity × SES; multimorbidity × health insurance) (***Appendix Table 7 and 13***) rather than relying solely on separate subgroup estimates. For SES, the purpose of the subgroup analyses was to examine whether the association between multimorbidity and healthcare utilization shows a gradient across socioeconomic groups. For health insurance, the subgroup analyses aimed to highlight potential differences in associations between insured and uninsured individuals.

Q13: Survey/sample weights were not applied due to heterogeneity across studies, but this limits representativeness. The implications for external validity should be emphasized.

**Response:** Thanks for this suggestion. Because the construction of survey weights differs substantially across cohorts, we did not apply weights in the primary analyses. To address this concern, we conducted additional analyses using pooled data fitted with multilevel models and applying standardized weights across countries. These weighted results (***Appendix Figure 8***) were highly consistent with the main unweighted findings.

Q14: The longitudinal nature of the data is underused — the analyses resemble pooled cross-sections rather than examining trajectories of multimorbidity or utilization over time.

**Response:** Thanks for your point. The focus of our study was not to examine individual trajectories of multimorbidity or healthcare utilization over time. Instead, we used the longitudinal structure to account for within-individual correlation by incorporating individual random effects. Our aim was to leverage the repeated measures to improve estimation rather than to model longitudinal trajectories themselves.

Q15: SES and insurance analyses are presented descriptively through stratification, but formal interaction terms would strengthen the claim that these variables modify the association between multimorbidity and healthcare utilization.

**Response:** Thanks for pointing it out. We have now included interaction term analyses for SES and health insurance (e.g., multimorbidity × SES; multimorbidity × insurance), and the results are presented in ***Appendix Table 7 and Appendix Table 13***.

Q16: Variance components (e.g., intraclass correlation coefficients) from the random-effects models are not reported. These would help quantify country-level variation.

**Response:** Thanks for your point. We have now reported the variance components from the random-effects models in ***Appendix Table 8-11***.

Q17: Potential reverse causality (e.g., increased healthcare utilisation leading to more diagnoses and hence multimorbidity classification) is not addressed. This should be acknowledged as a limitation.

**Response:** Thanks for your this suggestion. Our study is observational and aims to describe associations rather than establish causality; therefore, we cannot rule out this possibility. We have now explicitly acknowledged this as a limitation in the manuscript.

Q18: Age and BMI are categorised rather than modelled continuously, which reduces statistical power and may introduce residual confounding.

**Response:** Thanks for your point. Age was modelled as a continuous variable in all analyses. We re-estimated the models treating BMI as a continuous variable and the results remained consistent with the main findings (see the following figure).

***Reviewer #2***

Q1: [Introduction] The scope of physical, psychological, and cognitive multimorbidity referred to in this paper should be defined at the beginning of the Introduction section since they are not consistently or clearly defined in the literature and overlaps may exist.

**Response:** We have revised the second paragraph of the ***Introduction*** to explicitly define the scope of these three domains and our conceptualization of multimorbidity in this study.

Q2: [Methods p14] "In this study, the complexity of multimorbidity was considered as the number of disorder types". It isn't clear whether the authors mean the number of individual conditions, or the number of domains (physical, psychological, cognitive) in which a respondent has at least one condition. From the way multimorbidity patterns are presented, it appears that they are counting domains, not conditions, and then classifying respondents into combinations of domains regardless of how many conditions are present within each. This distinction is important to clarify as it affects how "complexity" is defined and interpreted. Counting domains captures breadth across types of morbidity, whereas counting conditions captures overall disease burden. The choice between these approaches has implications for comparability with other studies and for the interpretation of the findings.

**Response:** Thank you for this comment. We have clarified this in the Measures – (1) ***Multimorbidity section***. In this study, “complexity” refers to the number of morbidity domains—physical, psychological, and cognitive—in which a respondent has at least one condition, rather than the total number of individual conditions.

Q3: [Methods] Regarding the outcome measures, there remain scope differences in how utilisation is defined (e.g., CHARLS includes health worker home visits as outpatient care, while KLoSA counts nursing homes and long-term care facilities as inpatient). These definitional inconsistencies are not resolved by standardising the timeframe. Also, annualising data collected with a 1 month recall period could introduce bias, since shorter recall windows could yield higher reported use due to reduced forgetting; this may reduce comparability with surveys using a 12 or 24-month frame.

**Response:** Thank you for this point. To address this, we conducted sensitivity analyses in which we **excluded countries with non-comparable definitions or recall periods** and re-estimated all models. As shown in the revised ***Appendix Figure 4*** (***Sensitivity Analyses***), the results remained consistent with the primary findings.

Q4: [Methods] The operationalisation of health insurance as a simple yes/no variable seems too coarse given the substantial variation in coverage across countries and schemes (ie. copayments, deductibles, supplementary private insurance, limits on covered services can all strongly influence actual healthcare utilisation even among those technically insured). Treating all insured respondents as equivalent may therefore mask important within-country and cross-country differences.

**Response:** Thank you for this comment. Because the available data do not allow for consistent differentiation of insurance types across cohorts, we operationalised health insurance as a binary variable (insured vs. uninsured) to maintain comparability. This approach captures the fundamental distinction between having any financial protection versus none, which remains meaningful across settings despite structural differences. We now explicitly acknowledge that dichotomisation may mask important country differences. This limitation has been strengthened in the ***Limitations*** section.

Q5: [Methods] For alcohol use, some datasets consider "ever use", others consider "current use", one restricts to the past 12 months and one mixes weekly vs last 7 days depending on the wave. These differences mean that the binary yes/no variable is not measuring the same behaviour across studies. Similarly with tobacco use, "current smoking" vs "ever smoking" or a combination. Physical activities capture very different concepts and frequency thresholds. This has not been discussed in the limitations.

**Response:** Thank you for this comment. We have now acknowledged this limitation explicitly in the revised ***Limitations*** section.

Q6: [Methods] All countries are pooled together with "country-level variations accounted for as control variables". Given the very different health systems and definitions of outcomes, treating country as just a control variable may not be sufficient. Random slopes/hierarchical/multilevel models might better capture cross-country heterogeneity.

**Response:** Thank you for this comment. In response, we re-estimated the pooled analyses using multilevel models with countries specified as random intercepts to better account for between-country variation. The multilevel results were highly consistent with the main findings (***Appendix Figure 7***).

Q7: [Methods/Results/Discussion] The finding that people with complex multimorbidity including cognitive disorders had lower healthcare use than those with one or without conditions is striking and counterintuitive. It deserves more careful exploration and interpretation. Screening tests are not the same as clinical diagnosis. Cognitive disorder here reflects screening-based impairment, which could explain why utilisation patterns may not match clinical expectations. Misclassification could especially bias results if individuals with mild impairment fall above or below the arbitrary cut-off inconsistently across instruments.

**Response:** Thank you for this comment. In the revised **Discussion**, we have expanded our explanation.

Q8: [Discussion] Given the above, the reviewer would advise against suggesting too strongly that the results for the cognitive disorder population demonstrate unmet need, since the authors acknowledge direct evidence is lacking, framing it as a plausible interpretation would be safer.

**Response:** Thank you for this comment. In the revised ***Discussion***, we have softened the wording. We added the alternative explanations—such as misclassification due to screening-based measures.

Q9: [Discussion/Conclusion] The authors stress the importance of person-centred, integrated care for multimorbidity, which is supported in the wider literature. However, this conclusion is not directly demonstrated by their results, which focus on utilisation patterns and the role of insurance. It would be helpful if the authors clarified that their recommendation draws on broader evidence or more explicitly linked their own findings to the case for integrated care.

**Response:** Thank you for this comment. In the revised ***Discussion***, we have softened the wording and clarified that our recommendations draw on broader evidence rather than being directly demonstrated by our analyses.

Q10: [General] The manuscript requires a thorough proofread (e.g., "the complexities multimorbidity beyond " (p21); "those including by cognitive disorders" (p22), mix of American/British spellings, etc)

**Response:** Thank you for highlighting these. We have carefully proofread the entire manuscript.

***Reviewer #3:***

Q1: The major comments are that the results showing that those with the most complex patterns had lower use of healthcare than those with single conditions seems implausible. I think that the analysis should be checked. Could the outcomes be measured in the oldest participants for example, as a sense check, given that they have the highest prevalence of complex multimorbidity? Is the text describing this finding in results contradictory with Figure 2 that shows the probability of healthcare utilisation by different type of multimorbidity and that the more complex forms of multimorbidity have higher probability of health care use? The inclusion of health insurance as a variable is problematic for such a large group of countries especially when what having or not having health insurance in these countries will mean very different things. The interpretation of this finding across such a heterogenous group of countries is not clear.

**Response:** Thank you for raising this point. We have thoroughly reviewed our analyses and clarified the distinction between crude utilisation proportions (Figure 2) and adjusted associations from regression models (Figure 3). Because these figures represent different metrics (unadjusted vs. adjusted), the ranking of multimorbidity groups differs between them; this has now been stated in the revised manuscript.

To further assess the robustness of our results, we conducted an additional sensitivity analysis by restricting the sample to adults aged ≥65 years. The results remained consistent with the primary findings (see ***Appendix Figure 9***).

Q2: I think that the methods section states that six cohort studies were included by only five are listed.

**Response:** Thank you for pointing this out. You are correct that five cohort studies were included. The inconsistency has been corrected in the revised manuscript.

Q3: Management of missing data by using complete case analysis, how many participants were excluded and did this result in introduction of any bias, were these individuals lower socioeconomic status compared to those included for example?

**Response:** Thank you for this comment. The ***Appendix Table 6*** reports the proportion of missing values for each variable and the number of participants excluded in the complete-case analysis. In addition, multiple imputation (20 datasets) was conducted as a sensitivity analysis, and the results were highly consistent with the main findings, further supporting the robustness of our conclusions (***Appendix Table 10***).

Q4: Outcome - what was the duration of follow-up for outpatient or inpatient care use - this is described for the count models but not the logistic regression models. Standardised the utilisation of health care, could the methods for this approach be further explained?

Response: Thank you for this point. Specifically, outpatient care referred to utilisation within the past 12 months for all datasets except CHARLS (1 month) and KLoSA (24 months), and inpatient care referred to utilisation within the past 24 months across all studies. To enable cross-country comparability, we standardised utilisation measures by converting all datasets to a consistent one-year (outpatient) and two-year (inpatient) reference period before analysis. We have now added clarifying text to the ***Methods*** section.

Q5: Health insurance - how was the lack of need for health insurance in some of the included studies, or differences in the coverage and implications of having or not having health insurance across the countries incorporated into the models?

**Response:** Thank you for this comment. In this study, health insurance was harmonised as a binary indicator (covered vs not covered) due to substantial cross-country heterogeneity in insurance schemes. We acknowledge that insurance coverage does not represent an equivalent level of financial protection across countries. To minimise bias arising from these differences, we (1) Country fixed effects in pooled analyses, controlling for unobserved, system-level differences across countries. (2) Multilevel (hierarchical) models as an additional sensitivity analysis, allowing random variation at both the individual and country levels. These results were consistent with our main findings (***Appendix Figures 7–8***). We have now expanded the ***Limitations*** section to explicitly acknowledge that harmonising insurance status as a binary variable may mask important cross-national differences, despite the modelling approaches used to mitigate this issue.

Q6: Covariates - can the data type for household wealth level be described as for the other covariates, was this categorised and if so what were the categories?

**Response:** Thank you for this comment. We have now clarified the treatment of household wealth in the Methods section. Household wealth was used as a proxy for socioeconomic status (SES) and categorised into within-country, within-wave quartiles (Q1–Q4).

Q7: "we considered lifestyle factors … in the association between healthcare utilization and multimorbidity" - can you clarify what this means, were these covariates included in the models? Could you draw DAGs for each of the outcomes to show that these confounders are relevant to the exposure and outcomes? The text does not show the categories for physical exercise, alcohol, and tobacco. Could you either list the categories of all covariates in main text, or none in main text and all in supplementary?

**Response:** Thank you for this comment. We have clarified in the revised manuscript that all lifestyle factors were included as covariates in every regression model. We also unified the variable definitions by moving all categorisation details to ***Appendix Table 4***. To justify the inclusion of these covariates, we added a directed acyclic graph (***Appendix Figure 1***).

Q8: "Socioeconomic groups were categorised into quartiles based on household wealth" - does this mean that household wealth has been adjusted for twice given that it is listed as a standalone covariate as well?

**Response:** Thank you for raising this point. Household wealth was used only once in the models. To avoid double adjustment, we clarified in the revised manuscript (**Covariates**).

Q9: Statistical analysis - could you provide further explanation of the "panel data approach"? "accounting for individual differences as random variations over time" - individual differences in what?

**Response:** Thank you for this comment. The random-effects models account for repeated measurements of the same individuals and capture unobserved, time-invariant individual characteristics.

Q10: Binomial models were used to examine associtions with binomial models - why was this chosen over logistic regression, was the distribution checked?

**Response:** Thank you for this comment. Logistic regression was used in the main analysis for binary outcomes; however,we conducted a sensitivity analysis using random-effects probit models and reported the marginal effects. The probit results were highly consistent with the main findings, supporting the robustness of our conclusions (***Appendix Figure 5***).

Q11: "country-level variations accounted for as control variables" - which country-level variations were accounted for?

**Response:** Thank you for this comment. In the pooled analyses, we controlled for country-level unobserved heterogeneity by including country fixed effects. These fixed effects absorb all time-invariant differences across countries—such as health system structure baseline healthcare accessibility, and cultural patterns of care-seeking.

Q12: Results - it would be helpful to see a Table 1 describing the cohort size and population characteristics from each study. Characteristics from each country could be added to supplementary but is not essential.

**Response:** Thank you for this suggestion. We have now added a table summarising the cohort size and key population characteristics for each study. This information is provided in ***Appendix Table 5***.

Q13: What is the reason for physical- psychological-cognitive multimorbidity having such a wide range in prevalence between Finland and South Korea, is this a coding or ascertainment issue, or different age groups across the studies? This seems too wide a range to reflect variations between the countries.

**Response:** Thank you for this comment. This reflects the data but may also partly arise from methodological differences. In particular, cohorts use different screening tools and cut-offs for psychological and cognitive disorders. We have added this clarification to the ***Limitations*** section.

Q14: The analysis of the relationship between multimorbidity patterns and healthcare utilization is described as "rate" but a proportion/percentage is reported.

***Response:*** Thank you for pointing this out. You are correct that the term “rate” was imprecise. The outcomes in Figure 2 represent **proportions (percentages)** rather than true rates. We have revised the manuscript to replace “rate” with “proportion”.

Q15: Figure 4 - stratified by household wealth. The figure and legend do not state the order of the quintiles, presumably Q4 is the most affluent but it would be helpful to state this.

***Response:*** Thank you for this suggestion. We have now clarified in the Figure 4 note that the household wealth quartiles are ordered from Q1 (lowest wealth) to Q4 (highest wealth).
