# Supplementary material for: Multimorbidity, health service use, and health insurance by socioeconomic groups in 31 countries: A multi-cohort study
Source: PLoS Med. 2026 May 5;23(5):e1005087. doi: 10.1371/journal.pmed.1005087 (PMC13160435; doi:10.1371/journal.pmed.1005087)
Supplement: Attachment — Submitted filename: 2-Response_to_Reviewers_auresp_2.docx [file pmed.1005087.s006.docx]

Dear Reviewers,

We sincerely thank you for your time of our manuscript entitled “*Multimorbidity, health service use, and health insurance by socioeconomic groups in 31 countries: a multi-cohort study*” We greatly appreciate the detailed comments, which have substantially strengthened the clarity, methodological transparency, and interpretability of our work.

In this revision, we have undertaken extensive methodological and editorial revisions to address all concerns raised. These include additional sensitivity and diagnostic analyses, clarification of cohort definitions and variable harmonisation procedures, refinement of statistical modelling strategies, and substantial improvements to terminology, tables, and figures.

We provide detailed, point-by-point responses below and have clearly indicated all corresponding changes in the revised manuscript and Supporting Information.

***Reviewer #1:***

Q1: Despite extensive sensitivity analyses, the manuscript continues to report and interpret pooled effect estimates in the presence of extremely high between-country heterogeneity (I² frequently >90%). While multilevel models are presented as robustness checks, the manuscript does not sufficiently justify the meaningfulness of pooled odds ratios and IRRs under such conditions.

**Response:** Thank for this important issue. We have clarified that pooled meta-analytic estimates are presented as descriptive summaries of the distribution of country-specific associations rather than as homogeneous effect sizes. To further analyze heterogeneity, we conducted exploratory meta-regression analyses using country-level indicators (UHC index, GDP per capita, income inequality, and health expenditure). Across most multimorbidity categories, residual heterogeneity (I²_res) tended to decrease, although substantial heterogeneity remained, suggesting that cross-national variation is only partially attributable to macro-level health system characteristics (Appendix Tables 18-21). In addition, we re-estimated models using multilevel mixed-effects specifications with country-level random slopes (Appendix Figure 9). Findings were materially unchanged.

Q2: The analytical framework does not adequately address temporal ordering and time-varying confounding. It remains unclear whether key covariates (e.g., insurance status, SES, employment) are modelled contemporaneously or lagged, raising concerns about conditioning on post-exposure variables and potential bias.

**Response:** We appreciate this concern. In response, we conducted additional sensitivity analyses in which all covariates were fixed at baseline. Results were consistent with the primary analyses (Appendix Figure 12).

Q3: The manuscript still lacks explicit reporting of model diagnostics, particularly for the random-effects negative binomial models. Sensitivity analyses using alternative model families do not substitute for confirmation that key assumptions (e.g., overdispersion, convergence, adequacy of the random-effects structure) were assessed and met in the primary analyses.

**Response:** Thanks for this point. We have now reported model diagnostics. Overdispersion was assessed via the dispersion parameter (Appendix Tables 9 and 11), which was consistently significantly different from zero across countries, supporting the use of negative binomial over Poisson specifications. We also confirmed model convergence and examined the adequacy of the random-effects structure by comparing alternative specifications (random intercepts and slopes model) (Appendix Figures 7, 8 and 9). Findings were stable across specifications.

Q4: Residual non-comparability of healthcare utilisation definitions across cohorts remains a major limitation. Standardising recall periods does not resolve fundamental differences in what constitutes outpatient or inpatient care across studies. This issue remains under-emphasised in the main Methods and should be more prominently acknowledged as a threat to internal validity.

**Response:** Thanks for pointing this out. We agree that residual non-comparability across cohorts is an inherent limitation of multi-country survey harmonisation. We have strengthened the Methods and Limitations sections. We emphasise that country-specific analyses were conducted prior to meta-analysis to minimise cross-system comparability bias, and pooled estimates are interpreted descriptively rather than as implying equivalence of healthcare constructs across settings.

Q5: The continued use of complete-case analysis as the primary approach, with multiple imputation relegated to sensitivity analyses, remains problematic given the scale and likely non-random nature of missingness across cohorts. The potential for selection bias—particularly by socioeconomic status and health status—has not been sufficiently explored.

**Response:** We thank for this important point. In the revised manuscript, multiple imputation results by chained equations are now used as the primary results (Figure 3). Complete-case analyses are presented as sensitivity analyses (Appendix Figure 11).

Q6: Cognitive and psychological disorders are identified using screening instruments with cohort-specific cut-offs, yet are treated as error-free binary exposures. No quantitative assessment of exposure misclassification (e.g., alternative cut-offs, exclusion of borderline cases) is provided, despite the centrality of cognitive disorders to the main conclusions.

**Response:** Thank you for this comment. To quantitatively assess this, we conducted additional sensitivity analyses using alternative thresholds for cognitive and psychological disorder. For SHARE and MHAS, cognitive disorder was defined using cohort-specific standardised thresholds; the primary definition required performance ≥1 SD below the cohort-specific mean on cognitive tests, while stricter and more lenient definitions used ≥1.5 SD and ≥0.5 SD thresholds, respectively. For other cohorts, instrument-specific absolute score cut-offs were used, and these were shifted in both stricter and more inclusive directions. For psychological disorder, stricter definitions required a higher symptom threshold and excluded borderline scores, whereas lenient definitions included subthreshold cases. Across all alternative specifications, the direction and magnitude of associations between multimorbidity and healthcare utilisation remained unchanged (Appendix Figures 13–14).

Q7: Although variance components are now reported, the manuscript does not discuss whether random slopes for key exposures were considered. Given pronounced cross-national heterogeneity, restricting country-level variation to random intercepts may be overly limiting and could obscure meaningful differences in associations.

**Response:** Thanks for this comment. As an additional robustness check, we fitted multilevel models with country-level random slopes; findings were consistent with the main results (Appendix Figure 9).

Q8: The manuscript does not clearly state whether robust or cluster-adjusted standard errors were used in panel models, particularly given repeated observations within individuals and clustering within countries.

**Response:** Thanks for pointing out this. We have clarified that standard errors were adjusted for clustering at the individual level.

Q9: While interaction terms for SES and health insurance are now included, the implications of multiple testing and limited power for interaction analyses across several outcomes are not sufficiently discussed.

**Response:** Thanks for this point. Specifically, formal interaction tests were conducted for multimorbidity × SES and multimorbidity × health insurance, and these results are reported in Appendix Tables 7 and 13. We acknowledge that interaction analyses are inherently less powered than main-effect analyses, particularly across multiple outcomes and countries. Accordingly, we have added text emphasising that statistically significant interaction findings should be interpreted cautiously. For the analyses examining differences between insured and uninsured individuals across SES strata (Appendix Figure 3), these were intended as descriptive comparisons to illustrate potential gradients rather than formal tests of SES × insurance interaction. We have revised the manuscript to clarify this. The primary conclusions of the study do not rely on these subgroup comparisons.

Q10: The rationale for preferring random-effects logistic regression over marginal models (e.g., GEE), particularly for policy-relevant average effects, is not provided.

**Response:** We thank for this methodological consideration. Our primary objective was to estimate longitudinal associations. Random-effects logistic regression was therefore selected to model subject-specific associations while retaining between-individual information. We acknowledge that marginal models estimate population-average effects that may be more directly aligned with policy interpretation. As a robustness analysis, we estimated marginal models (Appendix Figure 5). These results were consistent with the primary estimates.

Q11: Although marginal effects are presented in supplementary analyses, the continued emphasis on odds ratios for common outcomes (e.g., outpatient utilisation) risks misinterpretation and should be more clearly caveated in the main text.

**Response:** Thanks for pointing out this. We have added clarifying language in the main text and present marginal effects in supplementary analyses.

Q12: The potential impact of survey attrition is not discussed. Differential loss to follow-up by multimorbidity status or SES could bias estimates even when random effects are used.

**Response:** Thanks for this point. We added the follow-up rate in the Appendix Table 5, and we conducted supplementary analyses examining predictors of attrition. Early dropout was rare and was not significantly associated with multimorbidity status, socioeconomic position, age, or gender (Appendix Table 21).

Q13: The manuscript does not report whether collinearity diagnostics were assessed, despite inclusion of correlated socioeconomic variables (wealth, education, employment, insurance) in the same models.

**Response:** Thanks for this point. We assessed multicollinearity among socioeconomic variables using variance inflation factors (VIF) (see the following table). And, it suggests that collinearity did not influence estimates.

Table. The VIF values of socioeconomic variables

|  | Outpatient | Inpatient | Number of outpatient visits | Number of inpatient admissions |
| --- | --- | --- | --- | --- |
| Health Insurance (ref: no) | 4.516618 | 4.520188 | 4.548606 | 4.816867 |
| Educational Attainment (ref: middle school or below) |  |  |  |  |
| High school | 1.600473 | 1.598725 | 1.604204 | 1.593949 |
| College or above | 1.900906 | 1.897999 | 1.907249 | 1.899279 |
| Household wealth (ref: Q1) |  |  |  |  |
| Q2 | 1.55049 | 1.549269 | 1.55215 | 1.55186 |
| Q3 | 1.6219 | 1.620377 | 1.623428 | 1.624661 |
| Q4 | 1.756207 | 1.754519 | 1.757596 | 1.760465 |
| Work status(ref: no) | 1.497235 | 1.498089 | 1.498822 | 1.507804 |

Q14: For count outcomes, the authors do not clarify whether zero-inflation was assessed or whether alternative count models (e.g., hurdle or zero-inflated models) were considered, particularly in countries with high proportions of non-users.

**Response:** Thanks for this point. We estimated hurdle models (logistic model for any use and truncated negative binomial for intensity). Results were consistent with the primary models (Appendix Figure 15).

***Reviewer #2:***

Q1: In Methods and throughout the manuscript, the authors refer to 5 studies being included (China Health and Retirement Longitudinal Study (CHARLS), the Japanese Study of Aging and Retirement (JSTAR), the Korean Longitudinal Study of Aging  (KLoSA), the Mexican Health and Aging Study (MHAS) and the Survey of Health, Ageing  and Retirement in Europe (SHARE)). What are the additional studies (ELSA and HRS) mentioned in various places throughout the manuscript (e.g. sensitivity analyses and Appendix Tables 4 and 5)?  A previous reviewer (#3, comment 2) asked the authors about the same issue.

**Response:** Thank you for pointing this out. The primary analytic cohorts include HRS (USA), CHARLS (China), JSTAR (Japan), KLoSA (Korea), MHAS (Mexico), and SHARE (Europe). We have revised the Methods, Results, figure legends, and all relevant Appendix tables.

Q2: Results and Figures 1-3: how many people had multimorbidity consisting of physical -diseases only or psychological disorders only? There groups seem to have been overlooked in these results. Or, if the groups titled e.g. "psychological disorder" in fact represent people with multiple psychological disorders, how many people had only one psychological, only one physical and only one cognitive disorder?

**Response:** Thanks for this point. Our multimorbidity patterns are defined at the domain level (physical, psychological, cognitive). For each domain, participants are classified as having a disorder if at least one condition within that domain is present, regardless of the number of conditions within that domain. Therefore, “psychological disorder” refers to psychological-domain morbidity only (and not necessarily multiple psychological disorders).

Q3: Were dementias included in the cognitive disorders? If not, why? What were the implications of this to the findings? The authors allude to this in the discussion but it would be worth a more detailed note.

**Response:** Thank you for this question. In our harmonised definition, the cognitive domain was based on cohort-specific cognitive screening tests and cut-offs (e.g., MMSE). Clinical dementia diagnoses were not consistently available and comparable across cohorts, and in several studies dementia ascertainment relies on different reporting mechanisms (self-report vs physician diagnosis vs proxy report), which could introduce additional cross-cohort non-comparability. Therefore, we defined cognitive disorder using standardised cognitive screening thresholds. We have now clarified this in the Methods and expanded the Limitations.

Q4: Methods and throughout the manuscript: the authors will need to specify what outpatient healthcare means in the studies they have used in their analysis. Is this primary or secondary healthcare? Are public healthcare system appointments only included, or do the data include private (and privately paid for) or occupational (paid for by the employer in some European countries) healthcare appointments? What are the implications of including/excluding these data sources?

**Response:** Thank you for this important comment. We agree that outpatient utilisation is not fully comparable across health systems. We specify in Appendix Table 2 the exact survey wording used in each study. Where available, we clarify whether the survey questions refer broadly to “doctor visits” without distinguishing sector, or whether they explicitly include visits in public, private, or occupational healthcare settings.

However, the surveys do not consistently distinguish primary versus specialist care, nor do they uniformly separate public, private, or employer-funded services. As a result, we were unable to harmonise outpatient care by provider type or financing mechanism across all countries. We have therefore strengthened the Limitations. Importantly, because our primary analyses are conducted within-country before meta-analysis, differences in outpatient definitions across countries are less likely to bias country-specific associations, but they may influence the magnitude of between-country heterogeneity.

Q5: How were the associations of multimorbidity with healthcare utilisation examined in data from countries with universal healthcare - where essentially everyone (bar certain marginal groups, e.g. illegal immigrants, who are unlikely to participate in health surveys) are covered by health insurance?

**Response:** Thank you for this important comment. Our health insurance analyses were conducted using pooled data comparing insured versus uninsured individuals across countries. We acknowledge that in countries with near-universal health coverage, variation in insurance status is limited. To address this concern, we have clarified in the revised Methods and Discussion that insurance-related effect modification should be interpreted as reflecting individual-level financial protection status within each country.

Q6: Were ORs and IRRs pooled in meta-analyses? Pooling estimates of cross-sectional estimates (ORs) and incidence estimates (IRRs) does not make sense. Mathematically, we can combine these of course, but they measure different quantities.

**Response:** Thanks for this point. Separate random-effects meta-analyses were conducted for ORs (binary outcomes) and IRRs (count outcomes), and these estimates were never combined.

Q7: Appendix table 6: you are reporting some very small numbers and proportions of participants (fewer than 5 in many cells). Is it ethical to release this information in a publication? Could participants be identified?

**Response:** Thank you for raising this. We have revised Appendix Table 6. Specifically, we have suppressed or aggregated cells with counts <5 (e.g., reporting as “<5” and not providing exact percentages for those cells).

Q8: Figure 3, title: what is "health utilisation"? If the authors mean healthcare utilisation, this should be revised. Similarly, the title of appendix table: what is the "association between multimorbidity and outpatient"?  Again, what are "inpatient number" and "outpatient number" in Appendix table 12 -  numbers of patients or numbers of appointments? A previous reviewer (#2, comment 10) encouraged the authors to check the language throughout the manuscript, which the authors responded they had done. This appears to have been an overstatement.

**Response:** Thanks for pointing out this. We have undertaken a thorough language audit across the manuscript and Supporting Information. “Health utilisation” has been corrected to “healthcare utilisation” throughout. We have also revised “outpatient number” to the number of outpatient visits, and “inpatient number” to the number of inpatient admissions.

Q9: Formatting  of the appendix tables 8-11 needs to be revised for clarity.

**Response:** Thank you. We have reformatted Appendix Tables 8–11. And, we added consistent notes defining all parameters.

Q10: Your responses to a number of comments from the previous reviewers (e.g. language, ways to examine the impact having vs. not having health insurance, and the grouping and /or labelling of multimorbidity categories -  please see my major comment 2)  were incompletely addressed.

**Response:** We appreciate this feedback. In this revision, we have (i) conducted a full language audit and corrected terminology throughout; (ii) clarified the pooled insurance analysis strategy and its limitations in near-universal coverage settings; and (iii) revised the multimorbidity grouping/labels to explicitly reflect domain-based definitions.
