# Supplementary material for: Multimorbidity, health service use, and health insurance by socioeconomic groups in 31 countries: A multi-cohort study
Source: PLoS Med. 2026 May 5;23(5):e1005087. doi: 10.1371/journal.pmed.1005087 (PMC13160435; doi:10.1371/journal.pmed.1005087)
Supplement: Attachment — Submitted filename: 2-Response_to_Reviewers_auresp_3.docx [file pmed.1005087.s007.docx]

Dear Reviewers,

We sincerely thank you for your time of our manuscript entitled “*Multimorbidity, health service use, and health insurance by socioeconomic groups in 31 countries: a multi-cohort study*” We greatly appreciate the detailed comments, which have substantially strengthened the clarity, methodological transparency, and interpretability of our work.

In this revision, we have undertaken methodological and editorial revisions to address all concerns raised. These include additional sensitivity analyses, expanded reporting of model diagnostics, clarification of missing data handling, and improved reporting of contextual analyses..

We provide detailed, point-by-point responses below and have clearly indicated all corresponding changes in the revised manuscript and Supporting Information.

***Reviewer #1:***

Q1: The manuscript would benefit from a clearer articulation of the target estimand in the pooled meta-analytic analyses. While pooled estimates are now framed as descriptive summaries in the presence of substantial heterogeneity, it remains unclear whether the intended interpretation is a population-average effect across countries or merely a summary of country-specific associations.

**Response:** Thank you for this important comment. We have clarified the interpretation of the pooled meta-analytic estimates in the revised **Methods**.

Q2: Although multilevel mixed-effects models are presented, the manuscript does not report key variance components (e.g., random slope variance, intraclass correlation coefficients). Quantifying between-country variance would allow readers to assess the relative contribution of contextual versus individual-level factors and better interpret cross-national heterogeneity.

**Response:** Thank you for this helpful suggestion. We now report variance components from the multilevel mixed-effects models, including the random intercept variance, random slope variance, and ICC **(Appendix Table 19)**.

Q3: Model diagnostics are partially addressed (e.g., overdispersion, convergence), but additional evidence of model adequacy would improve transparency. Reporting comparative fit statistics (e.g., AIC/BIC across model families), marginal and conditional R² for mixed models, and brief summaries of residual diagnostics would enhance confidence in the chosen specifications.

**Response:** Thank for this point. We now report AIC and BIC across alternative models, including Poisson, negative binomial, and hurdle models in the **Appendix Table 24**. These comparisons support the use of the negative binomial models as the primary model.

Q4: The handling of missing data requires further clarification. The manuscript should explicitly report the extent and pattern of missingness across covariates and outcomes, the assumed missing data mechanism, and whether complete-case analysis or multiple imputation was employed. Given the cross-national and multi-cohort nature of the data, sensitivity analyses under alternative missingness assumptions would strengthen robustness.

**Response:** Thank you for raising point. Appendix Tables 5 and 6 now report the patterns of missingness for all covariates and outcomes. In the primary analyses, missing covariate values were handled using multiple imputation. Complete-case analyses were also conducted as sensitivity analyses (**Appendix Figure 11**), and the results were consistent with the primary analyses.

Q5: The issue of multiplicity remains insufficiently addressed. The study includes multiple multimorbidity categories, several utilisation outcomes, country-specific analyses, interaction terms, and meta-analytic summaries. The authors should discuss the risk of type I error inflation and clarify whether any formal or interpretive adjustments were considered.

**Response:** Thank you for this comment. We acknowledge that multiple comparisons. Because these analyses address research questions, we did not apply formal multiple-testing corrections. Instead, we interpret findings cautiously in **Discussion** section.

Q6: For count outcomes, while hurdle models were implemented as sensitivity analyses, the manuscript does not report the proportion of zero observations by country nor provide a clear justification for retaining the primary negative binomial specification over alternative frameworks. A more explicit comparison of model performance would strengthen methodological credibility.

**Response:** Thank you for this suggestion. We have reported the proportion of zero observations (binary outpatient and inpatient with no) by country in **Appendix Tables 5 and 6**. In addition, we compared model performance across Poisson, negative binomial, and hurdle models using AIC and BIC (**Appendix Table 24**).

Q7: The treatment of socioeconomic status (SES) warrants further consideration. If SES is categorised (e.g., wealth quartiles), the manuscript should clarify whether linear trends were tested or whether non-linearity was explored. The choice of functional form may materially influence effect estimates and equity interpretations.

**Response:** Thank you for this suggestion. We conducted sensitivity analyses treating SES as an ordinal variable. The results were consistent with the main analyses and are presented in **Appendix Figure 7**.

Q8: The interpretation of healthcare utilisation as an indicator of economic burden should be treated more cautiously. Visit counts and admission numbers are imperfect proxies for economic impact, particularly in the absence of harmonised cost data across countries with different service intensity and financing structures.

**Response:** Thank you for this comment. We have revised the **Discussion** to clarify this.

Q9: It would improve interpretability if incidence rate ratios were supplemented with marginal predicted counts (e.g., expected additional outpatient visits per year for individuals with multimorbidity). Translating relative measures into absolute differences would increase policy relevance.

**Response:** Thank you for this suggestion. We now report marginal predicted counts (**Appendix Table 7**).

Q10: Given cross-country variation in recall periods for healthcare utilisation, the manuscript should clarify whether utilisation measures were standardised (e.g., annualised) prior to modelling and how differences in survey wording may affect comparability.

**Response:** Thank you for this comment. We have clarified the recall periods used for healthcare utilisation. Differences in survey wording and recall periods across cohorts are summarised in **Appendix Table 2**.

Q11: The manuscript would benefit from a clearer distinction between formal insurance coverage and effective financial protection (e.g., cost-sharing, benefit depth), particularly when interpreting insurance-related effect modification in countries with near-universal coverage.

**Response:** Thank you for this comment. We have clarified this point in the revised **Discussion**.

Q12: Cross-level interaction analyses between multimorbidity and macro-level system indicators (e.g., UHC index, health expenditure per capita) could be presented more formally within the multilevel framework, rather than relying solely on post hoc meta-regression.

**Response:** Thank you for this suggestion. We agree that cross-level interaction analyses could provide additional insights. However, the primary purpose of including country-level indicators in our analyses was to examine potential sources of heterogeneity. Therefore, we used meta-regression to assess how country-level characteristics were associated with variation in country-specific estimates.
